# Baseline characteristics, analysis plan and report on feasibility for the Prevention Of Decline in Cognition After Stroke Trial (PODCAST)

**DOI:** 10.1186/s13063-015-1033-2

**Published:** 2015-11-07

**Authors:** Polly Scutt, Dan Blackburn, Kailash Krishnan, Clive Ballard, Alistair Burns, Gary A. Ford, Jonathan Mant, Peter Passmore, Stuart Pocock, John Reckless, Nikola Sprigg, Rob Stewart, Joanna M. Wardlaw, Philip M. Bath

**Affiliations:** Stroke Trials Unit, Division of Clinical Neuroscience, University of Nottingham, City Hospital campus, Hucknall Road, Nottingham, NG5 1 PB UK; Sheffield Institute for Translational Neuroscience, University of Sheffield, 385A Glossop Road, Sheffield, S10 2HQ UK; Wolfson Center for Age-Related Diseases, Wolfson Wing, Hodgkin Building, King’s College London, Guy’s Campus, London, SE1 1UL UK; Faculty of Medical and Human Sciences, Institute of Brain, Behavior and Mental Health, University of Manchester, Grafton Street, Manchester, M13 9NT UK; Medical Sciences Division, University of Oxford, John Radcliffe Hospital, Headley Way, Oxford, OX3 9DU UK; General Practice & Primary Care Research Unit, University of Cambridge, Addenbrooke’s Hospital, Forvie Site, Cambridge, CB2 0SR UK; Institute of Clinical Sciences, Queens University, Belfast, Royal Victoria Hospital, Block B, Belfast, BT12 6BA UK; Department of Medical Statistics, London School of Hygiene and Tropical Medicine, Keppel Street, London, WC1E 7HT UK; Department of Endocrinology, Royal United Hospital, Combe Park, Bath, BA1 3NG UK; King’s College London (Institute of Psychiatry, Psychology and Neuroscience), Department of Psychological Medicine (Box 63), De Crespigny Park, London, SE5 8AF UK; Centre for Clinical Brain Sciences, Western General Hospital, Crewe Rd, Edinburgh, EH4 2XU UK

## Abstract

**Background:**

A common complication after stroke is development of cognitive impairment and dementia. However, effective strategies for reducing the risk of developing these problems remain undefined. Potential strategies include intensive lowering of blood pressure (BP) and/or lipids. This paper summarises the baseline characteristics, statistical analysis plan and feasibility of a randomised control trial of blood pressure and lipid lowering in patients post-stroke with the primary objective of reducing cognitive impairment and dementia.

**Methods:**

The Prevention Of Decline in Cognition After Stroke Trial (PODCAST) was a multi-centre prospective randomised open-label blinded-endpoint controlled partial-factorial internal pilot trial running in secondary and primary care. Participants without dementia were enrolled 3–7 months post ischaemic stroke or spontaneous intracerebral haemorrhage, and randomised to intensive versus guideline BP lowering (target systolic BP <125 mmHg versus <140 mmHg); patients with ischaemic stroke were also randomised to intensive or guideline lipid lowering (target LDL cholesterol <1.4 mmol/L versus <3 mmol/L). The primary outcome was the Addenbrooke’s Cognitive Examination-Revised; a key secondary outcome was to assess feasibility of performing a large trial of one or both interventions. Data are number (%) or mean (standard deviation). The trial was planned to last for 8 years with follow-up between 1 and 8 years. The plan for reporting the main results is included as Additional file 2.

**Results:**

83 patients (of a planned 600) were recruited from 19 UK sites between 7 October 2010 and 31 January 2014. Delays, due to difficulties in the provision of excess treatment costs and to complexity of follow-up, led to few centres taking part and a much lower recruitment rate than planned. Patient characteristics at baseline were: age 74 (SD 7) years, male 64 (77 %), index stroke ischaemic 77 (93 %), stroke onset to randomisation 4.5 [SD 1.3] months, Addenbrooke’s Cognitive Examination-Revised 86 (of 100, SD 8), Montreal Cognitive Assessment 24 (of 30, SD 3), BP 147/82 (SD 19/11) mmHg, total cholesterol 4.0 (SD 0.8) mmol/L and LDL cholesterol 2.0 (SD 0.7) mmol/L, modified Rankin Scale 1.1 (SD 0.8).

**Conclusion:**

Limited recruitment suggests that a large trial is not feasible using the current protocol. The effects of the interventions on BP, lipids, and cognition will be reported in the main publication.

**Trial registration:**

ISRCTN85562386 registered on 23 September 2009

**Electronic supplementary material:**

The online version of this article (doi:10.1186/s13063-015-1033-2) contains supplementary material, which is available to authorized users.

## Background

Post-stroke cognitive decline and dementia are common, and potentially devastating for both patients and carers. Although lowering blood pressure (BP) and lipids and the use of antithrombotic therapy are known to reduce recurrence after ischaemic stroke [1–6], the effect of these and other interventions on cognition is unclear [7]. The trial designated Prevention Of Decline in Cognition After Stroke Trial (PODCAST) was designed to assess the safety and tolerability of intensive versus guideline BP and lipid lowering in ischaemic stroke and intensive BP versus guideline in haemorrhagic stroke and the feasibility of performing a large trial on whether intensive treatment reduces cognitive decline and dementia post stroke (Protocol, Additional file 1) [8].

### Aims

#### Start-up phase

- To determine the initial safety and the tolerability of intensive versus guideline BP and lipid lowering therapy.
- To determine the feasibility of recruiting and retaining sites and participants in a long-term dementia prevention trial involving patients with a previous stroke.
- To determine the feasibility of reaching and maintaining target BP and lipid levels and identify any barriers to achieving and maintaining BP and lipid targets.
- To determine the feasibility of performing recurrent cognitive assessment in clinic and by telephone.

#### Main phase

- To determine if ‘intensive’ blood pressure lowering therapy and/or ‘intensive’ lipid lowering therapy after stroke reduces cognitive decline and dementia.
- To determine if ‘intensive’ blood pressure lowering therapy and/or ‘intensive’ lipid lowering therapy after stroke reduces poor quality of life, poor function, depression, stroke recurrence, vascular events, and death.

## Methods

PODCAST was a multi-centre prospective randomised open-label blinded-endpoint controlled partial-factorial phase IV trial in secondary and primary care. Participants from 30 UK Stroke Research Network sites who were post ischaemic stroke or intracerebral haemorrhage by 3–7 months were included. All patients gave informed consent.

### Interventions

All patients were randomised (1:1) to intensive versus guideline blood pressure lowering (target systolic <125 mmHg versus <140 mmHg). Patients with an ischaemic stroke were also randomised (1:1) to intensive versus guideline lipid lowering (target low density lipoprotein-cholesterol <1.4 mmol/L versus <3 mmol/L). As a result, patients were randomised to one of six groups:

- Intensive BP lowering and intensive lipid lowering (ischaemic stroke only)
- Intensive BP lowering and guideline lipid lowering (ischaemic stroke only)
- Guideline BP lowering and intensive lipid lowering (ischaemic stroke only)
- Guideline BP lowering and guideline lipid lowering (ischaemic stroke only)
- Intensive BP lowering only (intracerebral haemorrhage only)
- Guideline BP lowering only (intracerebral haemorrhage only)

Intensive interventions were delivered in a secondary care/hospital research clinic; guideline interventions were delivered in primary care according to local practice.

### Primary outcome

Addenbrooke’s Cognitive Examination-Revised. This was assessed at each clinic visit and its analysis and presentation is described in the outline data tables (Additional file 2). It is a brief cognitive screening tool that was selected because it has greater sensitivity in detecting Alzheimer’s disease and is more sensitive than the Mini-Mental State Examination (MMSE) [9] and has been shown to be sensitive at detecting mild cognitive impairment (MCI) in post-stroke populations [10]. It is good at detecting visuospatial, fluency and executive dysfunction [11].

### Secondary outcomes

Feasibility of recruitment and retention of participants, tolerability and safety of the interventions, achieving and maintaining the blood pressure and lipid targets, maintaining differences in systolic blood pressure (>10 mmHg) and low density lipoprotein cholesterol (>1 mmol/L) between the treatment groups, and performing clinic and telephone follow-up of cognition measures. Additional tests of cognition were used:

- Stroop, Trail making. Additional measures of executive function were added since vascular cognitive impairment is known to have a greater effect on these cognitive domains and they better predict development of dementia and mortality [12–15].
- Montreal Cognitive Assessment (MoCA). This is widely used in post-stroke populations with reasonable sensitivity for detecting MCI and dementia [10, 16–18].
- Telephone MMSE (t-MMSE). This has been compared to the MMSE and has shown strong correlation. A score of 16 on the t-MMSE equates to a score of 19 on the MMSE. A score of 26 on the MMSE equates to a score of 23 on the t-MMSE [19].
- Telephone Interview for Cognitive Status (TICS). This has been validated in a post-stroke population [20].

### Blinding

Participants received open-label management. Cognition was assessed both unblinded (in clinic) and blinded (by telephone) to treatment. Adjudication of events (dementia, vascular, serious adverse events) was blinded to management. Recruitment of 600 participants was planned (300/BP group, about 270/statin group) to be sufficient to demonstrate whether sufficient on-treatment differences in BP and lipids can be obtained and maintained, and whether cognition can be assessed satisfactorily. More details of methodology can be found at the published protocol [8].

The study was conducted according to the principles of the Declaration of Helsinki and the International Conference on Harmonisation of Good Clinical Practice. The study was approval by the national research ethics committee (NRES Committee East Midlands – Nottingham 1, approval 09/H0403/71, date 12/11/2009).

### Eligibility

The full inclusion and exclusion criteria are given in the published protocol [8] and were designed to include a population who did not have known dementia but who were at higher risk of developing cognitive impairment and dementia on the basis of recent stroke and age, and who were likely to be able to attend follow-up for 5 years. Adult patients were eligible if they fulfilled all of the following criteria:

- Age >70 years and telephone MMSE (t-MMSE) >16
  - ° or age >60 and t-MMSE 17–20 (that is, indicator of impairment).
- Previously independent (mRS 0–2)
- Index event was ischaemic stroke (IS) or spontaneous intracerebral haemorrhage (ICH)
- Systolic blood pressure between 125–170 mmHg
- Total cholesterol 3–8 mmol/L
- 3–7 months post stroke onset
- Ability to give written informed consent prior to enrolment
- Had two informants who could support them if cognitive impairment developed

### Consent

Informed consent was obtained to undertake a screening cognitive assessment at 8–26 weeks using a telephone version of the MMSE; initially this was done by telephone but was later delivered in clinic. If screened positive, informed consent for the trial was obtained and the clinician used the secure web-based randomisation system to enter a patient into the study between 12 and 30 months post stroke.

### Randomisation

To reduce bias and optimise baseline matching between treatment groups, randomisation incorporated stratification (index event), minimisation (on baseline prognostic factors, as highlighted in Table 3), and simple randomisation (in 5 % of patients). Stratification and minimisation allow for improved matching at baseline, minimisation increases statistical power [21], and simple randomisation reduces predictability. The stratification and minimisation variables will be used for adjustment of the primary and secondary analyses. Following randomisation, the investigator was informed of the patient’s treatment allocation. Data entry during treatment used the same website and similar range and logic checks.

### Trial governance

The trial was supervised by a Trial Steering Committee, and run by a Trial Management Committee (based in Nottingham UK). An independent Data Monitoring Committee met and assessed safety and efficacy on three occasions. Experts, who were blinded to treatment assignment, adjudicated cognitive and dementia outcomes, vascular events, brain scans, and serious adverse events.

### Internet trial

The trial was designed as an electronic trial and was accessible via the following links:

- Trial website: http://www.podcast-trial.org
- Secure website for real-time data entry, validation and randomisation: https://www.nottingham.ac.uk/~nszwww/podcast/podcasttrialdb/podcast_login.php
- Demo website for investigators to practise data entry: https://www.nottingham.ac.uk/~nszwww/podcast/podcasttrialdb-demo/podcast_login.php (log-in: demoinv1, password: nottingham; pin: 8888)
- Resource website for investigators with all trial documents, including all protocol versions: http://www.podcast-trial.org/jevpybki.htm
- Stroop test website: https://www.nottingham.ac.uk/~nszwww/stroop_demo

Data were entered via a secure Internet site. However, some NHS sites could not access certain features, in particular the Stroop application that ran as a Java applet. Problems arose because of hospital firewalls and issues with Java updates on PC and Mac computers.

### Progress with the study and modifications to the design

The trial was designed to have two phases of recruitment and funding:

- Start-up phase (36 months, 2010–2013): Recruitment of 600 patients from 30 sites over 24 months with minimum follow-up of at least 12 months. In reality, 83 patients were recruited with the target reduced to 100 patients.
- Main phase (60 months): New funding to be sought for recruitment of 2,800 patients from 100 sites. Funding was never sought since the trial failed to recruit sufficient sites or patients at a rate that was feasible to support a large study.

Several protocol amendments were made, these covering the following issues:

#### August 2010

1. Addition of twice yearly email reminders to investigators to highlight the need to achieve targets in BP and lipid lowering in patients randomised to intensive treatment. This was done because the difference between intensive and guideline BP and lipid levels was not reaching target (>1 mmol/L LDL cholesterol and >10 mmHg systolic).

#### May 2012

1. Reduction in target lipid level: The level of target lipid concentration for intensive arm was reduced from LDL cholesterol <2.0 mmol/L to <1.3 mmol/L. This amendment was required because 50 % of patients were at or below the original target at baseline of 2.0 mmol/L, in part reflecting the high usage of statin therapy in this post-stroke population.
2. Revision of suggested guideline lipid lowering therapy (to simvastatin 10–40 mg, pravastatin 10–40 mg, fluvastatin 10–80 mg), and intensive group statins (to atorvastatin > 20 mg, any dose of rosuvastatin). This was due to revised NICE guidelines on lipid management (CG 67, 2008).
3. Addition of monitoring of serum glucose and HbA1c because some BP and lipid drugs may reduce, or cause, diabetes mellitus.
4. Introduction of ‘floating’ clinic appointments to follow planned regular research appointments if a patient randomised to one or both intensive arms had BP and/or lipid readings above target levels. These floating appointments allowed earlier/more aggressive treatment escalation.
5. Clarification that a standing blood pressure measurement should be done in clinic to detect postural hypotension.
6. Addition of NYHA levels 3 or 4 as an exclusion criterion
7. Removal of dementia-specific health-related Quality of Life (DEMQOL) as an outcome
8. Addition/inclusion of patients with posterior circulation infarcts (POCI). POCI was an original exclusion criterion because this group was felt unlikely to develop cognitive impairment. However cerebellar and brain stem infarcts are known to cause cognitive impairment.
9. Follow-up visits in clinic increased from once a year to every 6 months (with interval blinded telephone follow-up). Low recruitment meant that long-term follow-up in a large trial was unlikely, and so more and earlier visits were necessary to assess and escalate treatment for blood pressure and/or lipid.

#### June 2013

1. Reduction in sample size for the start-up phase from 600 to 100 patients.
2. Addition of an on-treatment CT scan at or after 1 year of treatment to assess potential changes in white matter disease and cerebral atrophy from pre-baseline.

Key milestones occurred as follows:

- January 2008: Trial planning commenced.
- July 2008: Joint funding application submitted to Alzheimer’s Society and Stroke Association.
- January 2009: Funding awarded.
- April 2009: Confirmation from UK Medicines and Healthcare products Regulatory Agency that trial is not a Clinical Trial of an Investigational Medical Product (CTIMP) and does not fall under the remit of the European Clinical trials Directive.
- November 2009: Approval by UK National Research Ethics Service (Nottingham Research Ethics Committee 1).
- September 2010: Commencement of funding.
- October 2010: Recruitment of first patient.
- September 2012: One year no-cost extension sought and obtained from funders because of low recruitment.
- November 2013: Protocol published [8].
- January 2014: Recruitment of last patient (that is, recruitment over 39 months).
- October 2014: Final follow-up of last patient. End of funding.

## Results

**Fig. 1:**
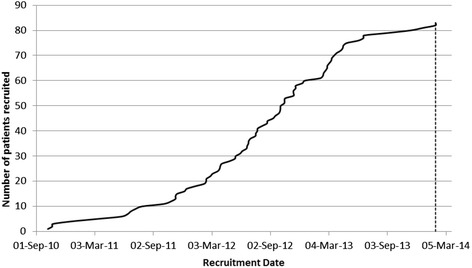
Recruitment throughout the trial

Only 83 participants were recruited (Fig. 1). The start-up phase did not recruit sufficient numbers to go to the full trial (planned recruitment of 600). A no-cost extension was requested with the aim of recruiting 100 participants. Funding for the main phase of the trial was never sought since the trial failed to recruit at a rate that was feasible to support a large study. Multiple reasons explain poor recruitment of both hospital sites and patients (Table 1). A key problem was the cost of intensive treatment, largely related to atorvastatin, which was proposed in the protocol for use in the intensive lipid control arm [8]. In many cases, the then primary care trusts (now clinical commissioning groups) refused to pay for atorvastatin. Nevertheless, none of these impediments damaged data integrity or validity.

**Table 1:** Recruitment issues with the trial

Issue	Explanation	Result/response
Insufficient sites recruited.	Primary Care Trusts refused to fund excess treatments costs, perceiving that some drugs, especially atorvastatin, would be too expensive. This was in spite of atorvastatin becoming generic midway through the trial.	Failure to recruit 30 hospital sites in start-up phase.
Insufficient patients recruited	Insufficient sites recruited.	Failure to recruit 600 patients in start-up phase, this precluding moving onto the main phase. The patient target was reduced to 100.
	Some sites only identified screen-negative patients (Table 2).	Protocol amended to allow inclusion of posterior circulation strokes.
	Exclusion criteria limited some recruitment; see screen failures (Table 2).	
	Follow-up of patients every 6 months meant that sites reached steady state of follow-up and, to maintain recruitment in other trials, ceased recruiting further patients into PODCAST.	

This table follows the format used for the MRC ENOS trial

### Baseline characteristics

106 patients failed screening with some patients having multiple reasons for screen failure (Table 2). Between 1 October 2010 and 31 January 2014, 83 patients were recruited from 19 sites in the UK; a further 5 sites screened patients but did not recruit a patient.

**Table 2:** Summary of screen failures and reasons

Reason for ineligibility	Number
Age 60–70 and t-MMSE > 20	45
Average systolic BP readings <125 mmHg	20
Vascular territory unknown	10
Clinical need for high dose statin	8
Too late: screening should be done before 26 weeks	8
Average systolic BP readings >170 mmHg	7
Too early: screening should be done after 8 weeks	6
Fasting total cholesterol <3 mmol/L	5
Age > 70 and t-MMSE < 17	5
Brain scan >10 days after index event (so precluding differentiation of ischaemic and haemorrhagic stroke)	4
Chronic renal failure/eGFR < 45	4
Modified Rankin Scale > 2	3
Clinical need for intensive BP	3
Consent could not be given	2
No informant available	1
Age 60–70 and t-MMSE < 17	1
Total	132

Patient characteristics at baseline are shown in Table 3; key baseline data included, given as number (%) or mean (standard deviation): age 74.0 (6.8) years, male 64 (77.1 %), index stroke ischaemic 77 (92.8 %), stroke onset to randomisation 4.5 [1.3] months, Addenbrooke’s Cognitive Examination-Revised 86.1 (7.7, maximum score 100), Montreal Cognitive Assessment 24.0 (2.6, maximum score 30), modified Rankin Scale 1.1 (0.8), blood pressure 147.1/82.1 (18.6/11.1) mmHg, total cholesterol 4.0 (0.8) mmol/L and LDL cholesterol 2.0 (0.7) mmol/L. All but one participant had one informant and a majority had two informants (Table 4).

**Table 3:** Baseline clinical characteristics. Data are number (%), median [interquartile range] or mean (standard deviation)

	With valid data	All/BP	IS/lipids	ICH
Number	83	83	77	6
Age (years) +	83	74.0 (6.8)	74.3 (6.6)	70.8 (8.4)
>75 (%)	83	42 (50.6)	39 (50.6)	3 (50.0)
Sex, male (%) +	83	64 (77.1)	61 (79.2)	3 (50)
Time to randomisation [months] +	83	4.5 [1.3]	4.5 [1.2]	3.8 [1.7]
<4 (%)	83	21 (25.3)	17 (22.1)	4 (66.7)
Medical history (%)				
Memory problem	77	34 (44.2)	30 (42.3)	4 (66.7)
If yes, length (months)	34	4.8 (2.7)	4.9 (2.9)	4.3 (1.5)
Depression, recent	83	4 (4.8)	4 (5.2)	0 (0.0)
Hypertension, treated				
Pre-index stroke	83	51 (61.4)	47 (61.0)	4 (66.7)
Post-index stroke	83	18 (21.7)	16 (20.8)	2 (33.3)
Neither	83	14 (16.9)	14 (18.2)	0 (0.0)
Hypertension treated with				
Life style measures only	69	0 (0.0)	0 (0.0)	0 (0.0)
Medications only	69	36 (52.2)	34 (54.0)	2 (33.3)
Both	69	30 (43.5)	26 (41.3)	4 (66.7)
Neither	69	3 (4.3)	3 (4.8)	0 (0.0)
Hyperlipidaemia	83	73 (88.0)	68 (88.3)	5 (83.3)
Life style measures only	73	1 (1.4)	0 (0.0)	1 (20.0)
Medications only	73	46 (63.0)	44 (64.7)	2 (40.0)
Both	73	24 (32.9)	22 (32.4)	2 (40.0)
Neither	73	2 (2.7)	2 (2.9)	0 (0.0)
Diabetes mellitus	83	17 (20.5)	16 (20.8)	1 (16.7)
Atrial fibrillation	83	15 (18.1)	15 (19.5)	0 (0.0)
Stroke	83	8 (9.6)	8 (10.4)	0 (0.0)
Transient ischaemic attack	83	16 (19.3)	15 (19.5)	1 (16.7)
Ischaemic heart disease	83	20 (24.1)	19 (24.7)	1 (16.7)
Myocardial infarction	83	11 (13.3)	11 (14.3)	0 (0.0)
Angina	83	18 (21.7)	17 (22.1)	1 (16.7)
Peripheral artery disease	83	5 (6.0)	5 (6.5)	0 (0.0)
Young stroke in first degree	83	10 (12.0)	10 (13.0)	0 (0.0)
Relative smoking (%)				
Current	83	5 (6.0)	5 (6.5)	0 (0.0)
Past	83	51 (61.4)	48 (62.3)	3 (50.0)
Never	83	27 (32.5)	24 (31.2)	3 (50.0)
Alcohol (%)				
>21	83	3 (3.6)	3 (3.9)	0 (0.0)
<21	83	56 (67.5)	52 (67.5)	4 (66.7)
None	83	24 (28.9)	22 (28.6)	2 (33.3)
Median [upw]	83	3 [0, 10]	4 [0, 10]	1 [0, 1]
BP tablets (%) +				
0	83	14 (16.9)	14 (18.2)	0 (0.0)
1	83	28 (33.7)	25 (32.5)	3 (50.0)
2	83	29 (34.9)	28 (36.4)	1 (16.7)
3	83	11 (13.3)	9 (11.7)	2 (33.3)
> = 4	83	1 (1.2)	1 (1.3)	0 (0.0)
Median [IQR]	83	1 [1, 2]	1 [1, 2]	1.5 [1, 3]
Lipid tablets (%)				
Any statin +	83	79 (95.2)	75 (97.4)	4 (66.7)
Atorvastatin	79	21 (26.6)	19 (25.3)	2 (50.0)
Fluvastatin	79	1 (1.3)	1 (1.3)	0 (0.0)
Rosuvastatin	79	2 (2.5)	2 (2.7)	0 (0.0)
Simvastatin	79	55 (69.6)	53 (70.7)	2 (50.0)
Pre-morbid mRS (%) + mRS 0	83	20 (24.1)	18 (23.4)	2 (33.3)
mRS 1	83	40 (48.2)	37 (48.1)	3 (50.0)
mRS 2	83	19 (22.9)	18 (23.4)	1 (16.7)
mRS > 2 ^	83	4 (4.8)	4 (5.2)	0 (0.0)
Mean (SD)	83	1.08 (0.8)	1.10 (0.8)	0.83 (0.8)
Hand dominance (%) Right	83	76 (91.6)	70 (90.9)	6 (100.0)
Left	83	6 (7.2)	6 (7.8)	0 (0.0)
Both	83	1 (1.2)	1 (1.3)	0 (0.0)
Side of weakness, left (%)	63	32 (50.8)	30 (50.0)	2 (66.7)
NIHSS (%) Mean (SD) +	83	0.77 (1.1)	0.78 (1.1)	0.67 (0.8)
Median [IQR]	83	0 [0,1]	0 [0,1]	0.5 [0,1]
OCSP classification (%) Total anterior +	83	6 (7.2)	6 (7.8)	0 (0.0)
Partial anterior	83	36 (43.4)	32 (41.6)	4 (66.7)
Lacunar	83	34 (41.0)	32 (41.6)	2 (33.3)
Posterior	83	7 (8.4)	7 (9.1)	0 (0.0)
Addenbrooke's Cognitive Exam-R +	83	86.1 (7.7)	86.0 (7.6)	87.5 (9.9)
MMSE	83	27.9 (2.0)	27.9 (1.9)	27.7 (2.7)
Telephone MMSE	83	20.4 (1.8)	20.4 (1.7)	20.3 (2.7)
Montreal Cognitive Assessment	83	24.0 (2.6)	24.1 (2.6)	22.7 (2.5)
TICS-M	83	24.1 (4.2)	24.3 (4.2)	22.2 (2.6)
Stroop: Part 1 time (sec)	83	54.7	54.6 (20.5)	55.5 (23.6)
Stroop: Part 1 score	83	(20.6) 0.97 (0.0)	0.97 (0.0)	0.99 (0.0)
Stroop: Part 2 time (sec)	83	46.1	46.0 (19.9)	46.6 (31.5)
Stroop: Part 2 score	83	(20.7) 0.97 (0.0)	0.97 (0.0)	0.96 (0.1)
Stroop: Part 3 time (sec)	83	66.9	67.3 (34.8)	62.2 (32.9)
Stroop: Part 3 score	83	(34.5) 0.83 (0.2)	0.83 (0.2)	0.79 (0.3)
Stroop: Interference time (Parts 3-2) (sec)	83	20.9	21.3 (22.3)	15.6 (21.2)
Stroop - Interference score (Parts 32)	83	(22.1) -0.14 (0.2)	-0.14 (0.2)	-0.17 (0.3)
Trail making A time (sec)	83	57.1	58.1 (30.7)	43.7 (12.0)
Trail making A score	83	(30.0) 24.6 (2.8)	24.6 (2.9)	25.0 (0.0)
Trail making B time (sec)	83	155.5	151.5	207.2
Trail making B score	83	(90.8) 22.1 (5.5)	(78.9) 21.9 (5.7)	(192.6) 24.8 (0.4)
IQCODE (from informant)	83	3.0 (0.5)	3.0 (0.5)	3.2 (0.2)
Systolic BP (mmHg) +	83	147.1	147.7	139.8
<140 (%)	83	(18.6) 32 (38.6)	(18.6) 30 (39.0)	(18.3) 2 (33.3)
<125 (%)	83	9 (10.8)	8 (10.4)	1 (16.7)
Diastolic BP (mmHg)	83	82.1 (11.1)	82.3 (11.1)	80.1 (13.1)
Heart rate (bpm)	83	71.5 (14.2)	71.7 (14.1)	69.5 (16.7)
Lipids (mmol/L)				
Total cholesterol +	83	4.0 (0.8)	4.0 (0.8)	4.3 (1.2)
Triglycerides	82	1.3 (0.6)	1.3 (0.6)	1.6 (0.6)
HDL cholesterol	81	1.4 (0.5)	1.4 (0.5)	1.5 (0.5)
LDL cholesterol	80	2.0 (0.7)	2.0 (0.7)	1.9 (1)
Non-HDL cholesterol	81	2.6 (0.8)	2.6 (0.8)	2.8 (0.8)
Estimated GFR (ml/min)	79	67.5 (11.6)	68.0 (11.3)	60.8 (13.7)
Glucose (mmol/L)	77	5.6 (1.6)	5.6 (1.6)	5.7 (2.0)

Percentages are calculated from the number of patients with data for that particular variable

+ Minimisation variable - from June 2013 limited to age, ACE-R, systolic blood pressure and total cholesterol because of low recruitment ^ Protocol violation

BP: blood pressure; bpm: beats per minute; HDL: high density lipoprotein; IS: ischaemic stroke; ICH: intracerebral haemorrhage; LDL: low density lipoprotein; MMSE: Mini-Mental State Examination; MoCA: Montreal Cognitive Assessment; NIHSS: National Institutes of Health Stroke Scale; OCSP: Oxfordshire Community Stroke Project; TICS-M: Telephone Interview for Cognitive Status-modified

Memory problem: As reported by patient or informant in response to direct questioning, and information from hospital records

MoCA: Estimated from ACE-R with addition of missing questions for attention (reading list of digits forwards and backwards, reading list of letters and tap with hand at each letter A) and abstraction (similarity between banana and orange, similarity between train and bicycle) and use of the ACE-R, not MoCA, picture of an animal. Non-HDL cholesterol = total cholesterol – HDL cholesterol

**Table 4:** Identity of first and second informants in 83 participants

Informant	1	2
Wife/partner	52	3
Husband/partner	14	0
Daughter/step-daughter	11	16
Son/step-son	4	12
Friend/neighbour	2	5
Sibling	0	2
Other	0	4
No informant	0	41

### Reporting of results by allocated treatment

The database was locked and the trial unblinded and an interim analysis performed in November 2014, as per the SAP. Interim main results were reported at the UK Stroke Forum in early December 2014. Full results (presented in Additional file 2) will be submitted for publication once the final analysis has been performed. Summary and individual patient data from PODCAST will be shared with relevant Cochrane and other systematic reviews.

## Discussion

Having recruited 83 patients, PODCAST did not achieve its adjusted recruitment target of 100 patients. As a result, the aim of assessing the feasibility of delivering the main phase was not met. Other aims, including the ability to obtain and then maintain differences in BP and lipid levels between intensive and guideline groups, ability to collect cognition scores during regular clinic and telephone follow-up, and incidence rates of cognitive impairment and dementia, will be reported in the main results.

## Conclusions

Future trials of dementia prevention in post-stroke populations are required because 20–30 % of stroke survivors will have dementia [22], but >50 % of patients will have cognitive impairment [13, 14]. Cognitive impairment, especially executive dysfunction, results in impaired function, distress to patients and carers and is associated with worse prognosis [23–25]. However, the post-stroke population is very heterogeneous. Also rates of dementia at or before 1 year post-stroke range from 41.3 % in hospital series without excluding prior dementia to 7.4 % in community series of first ever stroke where prior dementia was excluded [23]. Importantly, participants appear to be keen to take part in this type of study, as post-stroke cognitive impairment is one of the silent unmet needs in >40 % of stroke survivors at 1 year [26].

## Additional files

Additional file 1:
**Trial protocol.** (PDF 2304 kb) Additional file 2:
**Plan for reporting the main results.** (DOCX 117 kb)

## Abbreviations

ACE-R: Addenbrooke’s Cognitive Examination-Revised
BP: blood pressure
BPM: beats per minute
EQ-5D: EuroQoL-5 dimensions
EQ-VAS: EuroQoL-Visual Analogue Scale
HUS: Health Utility Status (derived from EQ-5D)
ICH: intracerebral haemorrhage
MMSE: Mini-Mental State Examination
MoCA: Montreal Cognitive Assessment
mRS: Modified Rankin Scale
NIHSS: National Institutes of Health Stroke Scale
PODCAST: Prevention Of Decline in Cognition After Stroke Trial
SAP: statistical analysis plan
SBP: systolic BP
TICS: Telephone Interview Cognition Scale
TOAST: Trial of ORG 10172 in Acute Stroke Treatment
ZDS: Zung Depression Scale
