# Supplementary material for: Baseline characteristics, analysis plan and report on feasibility for the Prevention Of Decline in Cognition After Stroke Trial (PODCAST)
Source: Trials. 2015 Nov 7;16:509. doi: 10.1186/s13063-015-1033-2 (PMC4636808; doi:10.1186/s13063-015-1033-2)
Supplement: Additional file 1: — Trial protocol. (PDF 2304 kb) [file 13063_2015_1033_MOESM1_ESM.pdf]

# Prevention Of Decline in Cognition After Stroke Trial (PODCAST): A factorial randomised controlled trial of intensive versus guideline lowering of blood pressure and lipids

**Version 1.5, 28 February 2012**

| | |
|---|---|
| Short title: | Prevention Of Decline in Cognition After Stroke Trial |
| Acronym: | PODCAST |
| Trial Registration: | ISRCTN85562386 |
| EUDRACT: | None – No Clinical Trials Authorisation required † |
| Ethics Reference: | 09/H0403/71 |
| Sponsor Reference: | 09012 |
| Trial Sponsor: | University of Nottingham |
| Funding Source: | The Stroke Association UK, Alzheimer's Society UK |
| Website: | www.podcast-trial.org/ |

† MHRA has confirmed that the trial is not within the scope of the Clinical Trials Directive.

This protocol is confidential and the property of the University of Nottingham. No part of it may be transmitted, reproduced, published, or used by others persons without prior written authorisation from the University of Nottingham

# TRIAL PERSONNEL AND CONTACT DETAILS

| | |
|---|---|
| Sponsor: | The University of Nottingham |
| Contact name | Mr Paul Cartledge<br>Head of Research Grants and Contracts<br>Research Innovation Services<br>King's Meadow Campus<br>Lenton Lane<br>Nottingham NG7 2NR |
| Chief investigator:<br>(Medical expert) | Professor Philip Bath<br>The Stroke Association Professor of Stroke Medicine<br>BSc MBBS MD FRCPath FRCP FESC<br>Phone: 0115 8231765<br>Fax: 0115 8231767<br>Email: Philip.Bath@nottingham.ac.uk |
| Trial Coordinating Centre: | Division of Stroke<br>University of Nottingham<br>Clinical Sciences Building<br>City Hospital<br>Hucknall Road Nottingham NG5 1PB<br>Phone: 0115 8231671<br>Fax: 0115 8230273<br>Email: podcast@nottingham.ac.uk |

This protocol is confidential and the property of the University of Nottingham. No part of it may be transmitted, reproduced, published, or used by others persons without prior written authorisation from the University of Nottingham

## SYNOPSIS

| | |
|---|---|
| Title | Prevention of decline in cognition after stroke trial: a factorial randomised controlled trial of blood pressure and lipid lowering |
| Short title | **P**revention **O**f **D**ecline in **C**ognition **A**fter **S**troke **T**rial (PODCAST) |
| Acronym | PODCAST |
| Chief Investigator | Professor Philip Bath |
| Objectives | Primary: To determine if 'intensive' blood pressure lowering therapy, and/or 'intensive' lipid lowering therapy, after stroke reduces cognitive decline and dementia.<br>Secondary: To determine if 'intensive' blood pressure lowering therapy, and/or 'intensive' lipid lowering therapy, after stroke reduces poor quality of life, poor function, depression, stroke recurrence, vascular events, and death. |
| Trial Configuration | Prospective, randomised, open-label, blinded end-point, controlled, partial factorial, phase IV trial |
| Setting | Secondary care |
| Sample size estimate | Assuming overall significance $\alpha$=5%, power 1-$\beta$=90%, rate of cognitive decline in 'guideline' BP group = 25% and 'intensive' BP group = 20% (absolute risk reduction 5%, relative risk reduction 20%) at 5 years, we estimate a sample size of 3,400 participants for the whole trial (start-up and main phase). The lipid factor will assume the same relative risk reduction (20 %) but will have a lower statistical power (~86 %), as it will only involve participants with ischaemic stroke (~3,060) |
| Number of participants | 3,400 participants (1,700 per BP group, ~1,530 per lipid group), comprising a:<br>Start-up phase: 600 participants (300 per BP group, ~270 per lipid group)<br>Main phase: 2,800 participants (1,400 per BP group, ~1,260 per lipid group) |
| Eligibility criteria | Ischaemic stroke or primary intracerebral haemorrhage<br>3-7 months post stroke event<br>Age>70 and normal cognition ("telephone-MMSE" >16), or<br>Age 60-70 with "telephone-MMSE" 17-20/22 |
| Description of interventions | BP lowering strategy:<br>'Intensive' group – target SBP <125 mmHg<br>'Guideline' group – target SBP <140 mmHg |

This protocol is confidential and the property of the University of Nottingham. No part of it may be transmitted, reproduced, published, or used by others persons without prior written authorisation from the University of Nottingham

| | |
|---|---|
| | Treatments will use licensed BP-lowering interventions (including life style modifications and drugs)<br><br>2. Lipid lowering strategy:<br>'Intensive' group – target LDL-cholesterol <1.4 mmol/l (or total cholesterol <3.1 mmol/l if LDL-cholesterol cannot be calculated)<br>'Guideline' group –target LDL-cholesterol <3.0 mmol/l (or total cholesterol <5.0 mmol/l if LDL-cholesterol cannot be calculated)<br>Treatments will use licensed lipid-lowering interventions (including life-style modification and drugs) |
| Duration of trial | 8 years. The proposed start date is September 2010<br>Start-up phase: 3 years<br>Main phase: 5 years |
| Randomisation and blinding | Randomisation over a secure internet site<br>The trial is open-label with blinded end point |
| Outcome measures | Primary: Comparison of cognition (Addenbrooke's Cognitive Examination-Revised extended to include death) between 'intensive' and 'guideline' BP/lipid lowering groups<br>Secondary: Other cognitive assessments; Quality of life; Vascular events; Functional outcome; Depression; Death |
| Statistical methods | Outcomes will be analysed by multiple regression, ordinal logistic regression and binary logistic regression, depending on the measure, with adjustment for baseline stratification and minimisation variables |

This protocol is confidential and the property of the University of Nottingham. No part of it may be transmitted, reproduced, published, or used by others persons without prior written authorisation from the University of Nottingham

## ABBREVIATIONS

| | |
|---|---|
| ABPM | Ambulatory Blood Pressure Monitoring |
| ACEI | Angiotensin Converting Enzyme Inhibitor |
| ACE-R | Addenbrooke's Cognitive Examination-Revised |
| AE | Adverse Event |
| ALLHAT | Anti Hypertensive and Lipid Lowering Treatment to Prevent Heart Attacks Trial |
| ALT | Alanine transaminase |
| ASCOT | Anglo-Scandinavian Cardiac Outcomes Trial |
| AVM | Arterio-venous malformation |
| BHS | British Hypertension Society |
| BMI | Body Mass Index |
| BP | Blood Pressure |
| CADASIL | Cerebral Autosomal Dominant Arteriopathy with Subacute Infarcts and Leukoencephalopathy |
| CI | Chief Investigator |
| COPD | Chronic Obstructive Pulmonary Disease |
| CLRN | Comprehensive Local Research Network |
| CRF | Case Report Form |
| CSP | Coordinated System for obtaining NHS Permissions |
| CT | Computer axial Tomography (scan) |
| DMC | Data Monitoring Committee |
| ENOS | Efficacy of Nitric Oxide in Stroke |
| EMEA | European Medicines Agency |
| GCP | Good Clinical Practice |
| GP | General Practitioner |
| HbA1c | Glycosylated haemoglobin test |
| HR | Heart rate |
| HOT | Hypertension Optimal Treatment Trial |
| IQCODE | Informant Questionnaire on Cognition Decline in the Elderly |
| ICC | International Coordinating Centre |
| IIS | Informant Information Sheet |
| HDL | High Density Lipoprotein |
| LDL/LDL-c | Low Density Lipoprotein-cholesterol |
| MI | Myocardial Infarction |
| MMSE | Mini mental status examination |
| MRI | Magnetic Resonance Imaging |
| MRC | Medical Research Council |
| NHS | National Health Service |
| NICE | National Institute of Health and Clinical Excellence |
| NIHR | National Institute for Health Research |
| NINDS | National Institute of Neurological Disorders and Stroke |
| OCSP | Oxford Community Stroke Project |
| Od | Once daily |
| On | At night |

This protocol is confidential and the property of the University of Nottingham. No part of it may be transmitted, reproduced, published, or used by others persons without prior written authorisation from the University of Nottingham

| | |
|---|---|
| OAST | Optimising Analysis of Stroke Trials collaboration |
| OA-Cog | Optimising the Analysis of Cognition collaboration |
| PCT | Primary Care Trust |
| PI | Principle Investigator |
| PICH | Primary Intracerebral Haemorrhage |
| PIN | Postal Index Number |
| PIS | Participant Information Sheet |
| PP | Pulse Pressure |
| PRoFESS | Prevention regime for effectively avoiding second strokes Study |
| PROGRESS | Perindopril pROtection aGainst REcurrent Stroke Study |
| PSD | Post-Stroke Dementia |
| QOF | Quality and Outcomes Framework |
| ReDa | Research Database |
| REC | Research Ethics Committee |
| R&D | Research and Development department |
| RR | Relative Risk |
| RRR | Relative Risk Reduction |
| SAE | Serious Adverse Event |
| SBP | Systolic Blood Pressure |
| SHEP | Systolic Hypertension in Elderly Program |
| SPARCL | Stroke Prevention by Aggressive Reduction in Cholesterol Levels |
| STU | Stroke Trials Unit |
| Syst-Eur | Systolic Hypertension in Europe Trial |
| t-MMSE | telephone mini mental status examination |
| TC | Total Cholesterol |
| TG | Triglycerides |
| TMC | Trial Management Committee |
| TOAST | Trial of Org 10172 in Acute Stroke Treatment Trial |
| TSC | Trial Steering Committee |
| UE | Urea and electrolytes |

This protocol is confidential and the property of the University of Nottingham. No part of it may be transmitted, reproduced, published, or used by others persons without prior written authorisation from the University of Nottingham

# TABLE OF CONTENTS


**PREVENTION OF DECLINE IN COGNITION AFTER STROKE TRIAL (PODCAST): A FACTORIAL RANDOMISED CONTROLLED TRIAL OF INTENSIVE VERSUS GUIDELINE LOWERING OF BLOOD PRESSURE AND LIPIDS .................. 1**
**TRIAL PERSONNEL AND CONTACT DETAILS .......................................... 2**
**SYNOPSIS ........................................................................................ 3**
**ABBREVIATIONS ................................................................................ 5**
**TABLE OF CONTENTS........................................................................ 7**
**1 TRIAL OBJECTIVES AND PURPOSE ................................................ 9**
1.1 PURPOSE 9
1.2 PRIMARY OBJECTIVE 9
1.3 SECONDARY OBJECTIVES 9
**2 TRIAL DESIGN.................................................................................. 9**
2.1 TRIAL CONFIGURATION 9
2.2 OUTCOME MEASURES 10
2.3 RANDOMISATION AND BLINDING 11
2.4 DURATION OF THE TRIAL AND PARTICIPANT INVOLVEMENT 12
2.5 SELECTION AND WITHDRAWAL OF PARTICIPANTS 14
2.6 TRIAL TREATMENT AND REGIMEN 21
**3 STATISTICS..................................................................................... 29**
3.1 MINIMISATION OF BIAS 29
3.2 METHODS OF ANALYSIS 29
3.3 SAMPLE SIZE AND JUSTIFICATION 31
3.4 DEFINITION OF POPULATIONS ANALYSED 32
3.5 HEALTH ECONOMIC ANALYSIS 32
3.6 POTENTIAL ANALYSIS ISSUES 32
**4 ADVERSE EVENTS........................................................................... 33**
4.1 DEFINITIONS 33
4.2 CAUSALITY 34
4.3 RECORDING AND SAFETY REPORTING 35
4.4 SERIOUS ADVERSE EVENT (SAE) ADJUDICATION 36
4.5 PARTICIPANT REMOVAL FROM THE TRIAL DUE TO ADVERSE EVENTS 36
**5 TRIAL MANAGEMENT..................................................................... 36**
5.1 SPONSOR 36
5.2 COORDINATING CENTRE 36
5.3 TRIAL STEERING COMMITTEE (TSC) 37
5.4 DATA MONITORING COMMITTEE (DMC) 37
5.5 OUTCOME AND EVENT ADJUDICATION COMMITTEES 37
**6 ETHICAL AND REGULATORY ASPECTS ......................................... 38**
6.1 ETHICS COMMITTEE AND REGULATORY APPROVALS 38
6.2 INFORMED CONSENT AND PARTICIPANT INFORMATION 38
6.3 RECORDS 39
6.4 DATA PROTECTION 40
6.5 QUALITY ASSURANCE AND AUDIT 41
6.6 DISCONTINUATION OF THE TRIAL BY THE SPONSOR 42
6.7 STATEMENT OF CONFIDENTIALITY 43
6.8 PUBLICATION AND DISSEMINATION POLICY 43
6.9 USER AND PUBLIC INVOLVEMENT 44
**7 TRIAL FINANCES............................................................................ 44**
7.1 FUNDING SOURCES 44


This protocol is confidential and the property of the University of Nottingham. No part of it may be transmitted, reproduced, published, or used by others persons without prior written authorisation from the University of Nottingham

7.2 PARTICIPANT STIPENDS AND PAYMENTS 45
**8 SIGNATURE PAGES........................................................................46**
**APPENDICES ..................................................................................47**
APPENDIX A. ADDENBROOKE'S COGNITIVE EXAMINATION-REVISED (ACE-R) 47
APPENDIX B. MINI MENTAL STATE EXAMINATION (MMSE) 54
APPENDIX C. TELEPHONE VERSION OF MMSE (T-MMSE) 55
APPENDIX D. TELEPHONE INSTRUMENT FOR COGNITION SCALE-M 56
APPENDIX E. TRAIL MAKING TEST (TMT) PARTS A &B 58
APPENDIX F. MODIFIED RANKIN SCALE (MRS) 61
APPENDIX G. BARTHEL INDEX (BI) 62
APPENDIX H. EUROQOL 63
APPENDIX I: INFORMANT QUESTIONNAIRE ON COGNITIVE DECLINE IN THE ELDERLY (IQCODE) 65
APPENDIX J. ZUNG DEPRESSION RATING SCALE (SHORT) 68
APPENDIX K DEFINITIONS 69
**REFERENCES ..................................................................................72**

This protocol is confidential and the property of the University of Nottingham. No part of it may be transmitted, reproduced, published, or used by others persons without prior written authorisation from the University of Nottingham

# 1 TRIAL OBJECTIVES AND PURPOSE

## 1.1 PURPOSE

Develop interventions to prevent cognitive decline and dementia after stroke.

## 1.2 PRIMARY OBJECTIVE

To determine if 'intensive' blood pressure lowering therapy, and/or 'intensive' lipid lowering therapy, after stroke reduces cognitive decline and dementia.

## 1.3 SECONDARY OBJECTIVES

To determine if 'intensive' blood pressure lowering therapy, and/or 'intensive' lipid lowering therapy, after stroke reduces poor quality of life, poor function, depression, stroke recurrence, vascular events, and death.

# 2 TRIAL DESIGN

## 2.1 TRIAL CONFIGURATION

PODCAST is a multi-centre, prospective, randomised, open-label, blinded end-point, controlled, partial-factorial, phase IV trial. It will be performed in two phases: start-up and main.

The start-up phase will recruit 600 participants from 30+ UK Stroke Research Network Centres in 3 years. Assuming a 'go' decision at 34 months based on start-up feasibility, as assessed by data collected from the start-up phase, the trial will seamlessly proceed into the main phase with the same design for a further 5 years. The main phase will aim to recruit a further 2,800 participants from across 100 sites internationally. Separate permission for funding from the appropriate bodies will be sought for the second phase (as done in the ENOS trial ISRCTN 99414122, with funding moving from BUPA Foundation to MRC).

The start-up phase will assess feasibility in the UK:

- Delivering the protocol
- Recruiting 30+ centres and 600 participants
- Achieving and maintaining differences in systolic BP (≧10 mmHg) and LDL-cholesterol (≧1 mmol/l) between the 'intensive' and 'guideline' treatment groups
- Performing clinic and telephone follow-up of outcome measures
- Assess the sensitivity of ACE-R to change
- Tolerability and safety of interventions

This protocol is confidential and the property of the University of Nottingham. No part of it may be transmitted, reproduced, published, or used by others persons without prior written authorisation from the University of Nottingham

The main phase will assess efficacy with recruitment from both UK and international centres. Participants enrolled in the start-up phase will continue to be followed during the main phase. The trial is being discussed with other countries (including those taking part in the ongoing ENOS trial,(1) as well as France). Separate ethical review and permission will be sought in each participating country.

If the overall trial is positive for one or both 'intensive' interventions, then they can be implemented readily and inexpensively in the UK since the treatments are available and will be off patent.

## 2.2 OUTCOME MEASURES

### 2.2.1 *Primary outcome measure*

For each of BP-lowering and lipid-lowering arms, comparison between 'intensive' and 'guideline' groups, of cognition, assessed using the Addenbrooke's Cognitive Examination- Revised (ACE-R)(2), (a superset of the Mini-Mental State Examination, MMSE(3)). Certain memorable items in the ACE-R will be cycled at each time point - see working practice document.

### 2.2.2 *Secondary outcome measures*

For each of BP-lowering and lipid-lowering arms, comparison between 'intensive' and 'guideline' groups:

1. Dementia
    a. Using AD - NINCDS/ADRDA (4), VaD - NINDS-AIREN (5) and Dementia-ICD-10
    b. With/without recurrent stroke
2. Cognition
    a. Global – MMSE, t-MMSE, TICS (6)
    b. Association – trail making A/B (7, 8)
    c. STROOP test (8)
    d. Cognitive decline with/without recurrent stroke
    e. Ordinal cognition (MMSE>28/23-28/10-22/<10/dementia/dead)
    f. IQCODE (by informant) (9)
3. Quality of life – EuroQoL(10)
4. Depression (Zung) (11, 12)
5. Dependency (modified Rankin Scale, mRs) (13, 14)
6. Disability (Barthel Index, BI) (14, 15)
7. Stroke recurrence
8. Myocardial infarction
9. Composite vascular events (non-fatal stroke, non-fatal MI, fatal vascular)
10. Stroke: fatal/severe non-fatal/mild/TIA/none(16)
11. Myocardial infarction: fatal/non-fatal/angina/none (16)
12. Vascular: fatal/non-fatal/none (16)
13. Revascularisation (heart, limb, visceral/renal) or amputation
14. New Diabetes
15. New atrial fibrillation
16. Residence (home, institution), care package, informal family support

This protocol is confidential and the property of the University of Nottingham. No part of it may be transmitted, reproduced, published, or used by others persons without prior written authorisation from the University of Nottingham

17. Blood pressure (systolic BP, diastolic BP, pulse pressure, rate-pressure product)
18. Lipids (TC, TG, HDL, calculated LDL)
19. Neuroimaging (in a subset of participants)

### 2.2.3 *Safety outcome measures*

Comparison between 'intensive' and 'guideline' BP/lipid lowering groups:

1. Death
2. Falls (leading to fracture or hospitalisation)
3. Symptomatic hypotension
4. Myositis and rhabdomyolysis
5. SAEs

## 2.3 RANDOMISATION AND BLINDING

### 2.3.1 *Randomisation*

All participants eligible for inclusion and for whom consent has been obtained will be randomised centrally using a secure internet site in real-time. Randomisation will be performed using:

1. Stratification on stroke type (ischaemic stroke/PICH) and country
2. Minimisation on key prognostic/logistical baseline factors:
    a. Age (<70/≥70 yrs)
    b. Sex (female/male)
    c. Dysphasia, mild (no/yes)
    d. ACE-R (>85/≤85)
    e. Mean systolic blood pressure, sitting (<150/≥150 mmHg)
    f. Total cholesterol (<4.0/≥4.0 mmol/L)
    g. Function/dependency (mRS<1/≥1)
    h. Brain region (subcortex/cortex)
    i. Evidence of periventricular white matter lucency (e.g.leukoaraiosis) (no/yes)
    j. Time since index stroke (<140/≥140 days)
    k. Number of antihypertensive drugs (<2/≥2)
    l. Already on a statin (no/yes)

This approach ensures concealment of allocation, minimises differences in key baseline variables, and slightly improves statistical power.(17)

In the event that the website cannot be accessed, participants may be randomised by telephoning one of a series of emergency telephone numbers. These participants will be randomised without stratification or minimisation.

This protocol is confidential and the property of the University of Nottingham. No part of it may be transmitted, reproduced, published, or used by others persons without prior written authorisation from the University of Nottingham

### 2.3.2 Blinding

PODCAST is a trial of BP and lipid management post-stroke. Hence, it is not placebo-controlled and neither participants nor investigators will be blinded to treatment. However, outcome assessment will be assessed blinded to treatment assignment.

## 2.4 DURATION OF THE TRIAL AND PARTICIPANT INVOLVEMENT

The start up phase will run for 3 - 4 years with participant recruitment from 30 UKSRN sites = 1 participant/site/month) with average follow-up 2 years (minimum 1 year). The main phase will then run for a further 4 - 5 years (total 8 years). Participant involvement in the whole trial will range from 1-8 years depending on the time of recruitment (**See tables 1,2,3**).

**Table 1: Trial timeline: Start-up phase**

| Time (months) | -6-0 | 0-2 | 3-6 | 7-18 | 19-24 | 25-30 | 31-36 |
|---|---|---|---|---|---|---|---|
| Protocol | <> | | | | | | |
| Approvals | <> | | | | | | |
| Trial materials | <> | | | | | | |
| Site identification | < | = | > | | | | |
| Funding, TSA/AS | | < | = | = | = | = | > |
| Recruit participants | | < | = | = | = | = | > |
| DMC reviews | | | < | = | = | = | > |
| Feasibility reviews | | | | < | = | = | > |
| Interim analysis (blinded) | | | | | | | <> |

**Table 2: Trial timeline: Main phase**

| Time (months) | 37-42 | 43-48 | 49-54 | 55-60 | 61-66 | 67-72 | 73-78 | 79-84 | 85-90 | 91-96 |
|---|---|---|---|---|---|---|---|---|---|---|
| Further site identification | < | = | = | = | > | | | | | |
| Funding (source to be identified | | < | = | = | = | = | > | | | |
| Recruit participants | < | = | = | = | > | | | | | |
| DMC reviews | < | = | = | = | = | = | = | = | > | |
| Final data cleaning | | | | | | | | < | = | > |
| Analysis | | | | | | | | | | <> |

Nb; Participants enrolled in the start-up phase will continue to be followed up in the main phase.

This protocol is confidential and the property of the University of Nottingham. No part of it may be transmitted, reproduced, published, or used by others persons without prior written authorisation from the University of Nottingham

**Table 3: Participant measures: Start-up and main phase**

| Time (months) | Pre-screen | Screen | 0 | 1 | 2 | 3 | 6 | 12 | 18 | 24 | 30 | 36 | 42 | 48 | 54 | 60 | 66 | 72 | 78 | 84 | 90 | 96 |
|---|---|---|---|---|---|---|---|---|---|---|---|---|---|---|---|---|---|---|---|---|---|---|
| Inclusion | + | | + | | | | | | | | | | | | | | | | | | | |
| Consent | + | | + | | | | | | | | | | | | | | | | | | | |
| Randomise | | | + | | | | | | | | | | | | | | | | | | | |
| CT/MR scan | †† | | | | | | | | | < | ‡ | > | | | | | | | | | | |
| ECG | ††† | | | | | | + | | + | | + | | + | | + | | + | | + | | + | |
| BP, lipids | † | | | | | | | | | | | | | | | | | | | | | |
| Clinic | | | | | | | | | | | | | | | | | | | | | | |
| BP | | + | + | (+) | (+) | (+) | + | + | + | + | + | + | + | + | + | + | + | + | + | + | + | + |
| ABPM ‡ | | | + | | | | + | | + | | + | | + | | + | | + | | + | | + | + |
| Lipids * | | | + | | | (+) | + | + | + | + | + | + | + | + | + | + | + | + | + | + | + | + |
| Glucose * | | | + | | | | + | + | + | + | + | + | + | + | + | + | + | + | + | + | + | + |
| HbA1c * | | | + | | | | + | + | + | + | + | + | + | + | + | + | + | + | + | + | + | + |
| UE * | | | + | (+) | (+) | (+) | + | + | + | + | + | + | + | + | + | + | + | + | + | + | + | + |
| Cognition | + | + | + | | | | + | | + | | + | | + | | + | | + | | + | | + | + |
| Stroke, MI | | | | | | | + | + | + | + | + | + | + | + | + | + | + | + | + | + | + | + |
| SAEs | | | + | (+) | (+) | (+) | + | + | + | + | + | + | + | + | + | + | + | + | + | + | + | + |
| Informant | | | + | | | | + | | + | | + | | + | | + | | + | | + | | + | + |
| Telephone | | | | | | | | | | | | | | | | | | | | | | |
| Cognition | | | | | | | | + | | + | | + | | + | | + | | + | | + | | + |
| Stroke, MI | | | | | | | | + | | + | | + | | + | | + | | + | | + | | |
| SAEs | | | | | | | | + | | + | | + | | + | | + | | + | | + | | |
| Informant | | | | | | | | + | | + | | + | | + | | + | | + | | + | | |

ABPM: Ambulatory Blood Pressure Monitoring; BP: blood pressure; MI: myocardial infarction; SAEs: serious adverse events †: BP, lipids from index event †† Clinical scan for index stroke; ‡ In participating centres; (+) In intensive groups only; ††† Clinical ECG for index stroke; perform ECG at Baseline if ECG from index event not available; Telephone cognition scores will also be used in clinic at Baseline and end of trial to calibrate them against clinic-only measures; T=telephone. * 1 week before

This protocol is confidential and the property of the University of Nottingham. No part of it may be transmitted, reproduced, published, or used by others persons without prior written authorisation from the University of Nottingham

## 2.5 SELECTION AND WITHDRAWAL OF PARTICIPANTS

### *2.5.1 Recruitment (see figure 4)*

Participants will be recruited from hospital-based stroke services. The initial approach will be from a member of the participant's usual care team (which may include the investigator and/or research nurses). The investigator or their nominee, e.g. from the usual care team (including research team), will inform the participant about the trial and a participant information sheet will be provided. Patient and GP contact details will be collected. Informed consent will be taken from participants at this point of contact to perform a face-to-face assessment of cognition ("telephone-mini mental status examination") and function (modified Rankin scale) at 8-26 weeks after the stroke.

On the basis of the assessments, if the participant is eligible and interested, a participant information sheet, a substudy information sheet (if taking part), and informant information sheet, will be given to the participant; a blood test request form for fasting lipids, glucose, urea and electrolytes, and HbA1c will also be provided. The participant's GP will be informed about the study and a 'GP practice briefing sheet' (with details of GP involvement in the trial) posted to them. Should the GP have concerns about their patient participating in the study, they will be asked to contact the local hospital research centre. It is important to note that GPs will not be involved in screening and recruiting patients and therefore will not require Good Clinical Practice (GCP) certification.

Participants will be contacted a few days later to assess their views about participation in the trial and to answer any questions. If they have agreed, participants will be asked to have the blood test (for fasting lipids, glucose, urea and electrolytes, and HbA1c) done at their GP practice (with the blood test form provided). All participants and their informant (see **Section 2.5.5**) will be booked to come to the local hospital research centre for further discussion, and if agreeable, enrolment and randomisation into the study. There should be a minimum of 1 week between the screening assessment and randomisation, so as to give time for the GPs to report any concerns they may have regarding their patient participating in the study. It is assumed that most GPs will want to support their patients if they elect to take part in clinical research; however, if GPs refuse, such patients will be withdrawn from the trial.

This protocol is confidential and the property of the University of Nottingham. No part of it may be transmitted, reproduced, published, or used by others persons without prior written authorisation from the University of Nottingham

**Figure 4:**Trial Flow Chart – actions prior to and at randomisation

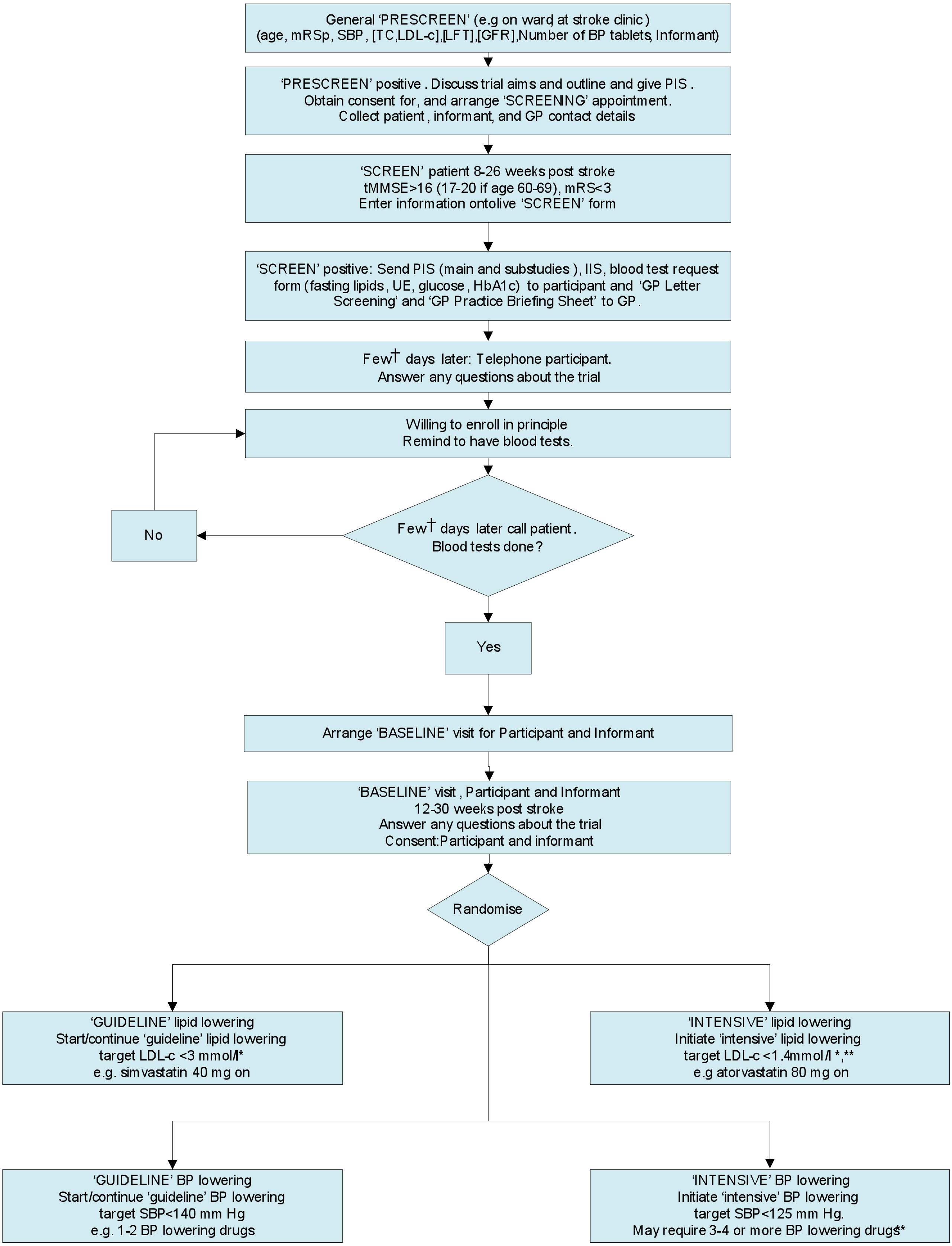


This protocol is confidential and the property of the University of Nottingham. No part of it may be transmitted, reproduced, published, or used by others persons without prior written authorisation from the University of Nottingham

† few - approximately 2-4 days

| Acronyms | Inclusion criteria | |
|---|---|---|
| BP | Blood pressure | - |
| GFR | glomerular filtration rate | ≥45 (eGFR≥37 in people of African/Afro-Caribbean origin) |
| LDL-c | LDL-cholesterol (fasting) | - |
| LFT | liver function test | ALT<u><3 times upper limit of normal, using local laboratories range</u> |
| mRs | modified Rankin Scale | <3 |
| mRsp | pre-morbid modified Rankin Scale | <3 |
| PIS | Patient Information Sheet | - |
| SBP | systolic blood pressure | 125-170 mmHg |
| TC | total cholesterol (fasting) | 3-8 mmol/l |
| t-MMSE | telephone Mini Mental State Examination | >16/22 if age >70<br>17-20/22 if age >60 |

| | |
|---|---|
| * | Only applies to patients with prior ischaemic stroke |
| ** | See management algorithms (intensive lipid and BP lowering working practice documents) |

### 2.5.2 *Inclusion criteria*

1. Age>70 years and "telephone-MMSE" >16; or age >60 years and "telephone-MMSE" 17-20/22
2. Functionally independent (mRS 0-2)
3. Ischaemic stroke .Strokes may be of any OCSP/TOAST type and in the anterior or posterior circulation.
4. 3-7 months post-event (to allow cognitive,(18) neurological, BP and lipid(19) stabilisation, but avoid attrition)
5. Systolic BP 125-170 mm Hg
6. Total cholesterol 3-8 mmol/l
7. Presence of an informant: partner, sibling, child, friend (for IQCODE)
8. Capacity and willingness to give consent

### 2.5.3 *Exclusion criteria*

1. Participants not meeting inclusion criteria
2. Subarachnoid haemorrhage
3. Secondary intracranial haemorrhage (trauma, AVM, cavernoma)
4. No CT/MRI within 10 days of index stroke
5. Inability to give consent or do study measures, e.g. severe dysphasia, weakness of dominant arm
6. Profound deafness
7. Severe hypertension (systolic BP>170 mmHg)

This protocol is confidential and the property of the University of Nottingham. No part of it may be transmitted, reproduced, published, or used by others persons without prior written authorisation from the University of Nottingham

8. Definite need for 'intensive' BP control
9. Severe hypercholesterolemia (TC>8 mmol/l)
10. Definite need for, or demonstrated intolerance of, 'high intensity' statin
11. Definite need for a cholinesterase inhibitor
12. Familial stroke associated with dementia, e.g. CADASIL
13. Chronic renal failure: eGFR<45 (or eGFR<37 in people of African/Afro-Caribbean origin)
14. Liver disease, ALT>3 times upper limit of normal, using local laboratories range
15. Ongoing participation in trials involving drug (including CTIMP trials) and/or devices. Participants already in another trial may be screened for PODCAST, provided the participation in the other trial is complete, prior to PODCAST randomisation.
16. Any serious medical co-morbidity (e.g. active malignancy) such that the life expectancy is <24 months
17. Clinically unstable at the time of enrolment
18. Dementia
19. NYHA classification of 3 or 4

### 2.5.4 *Informed consent*

All participants must have capacity, and be willing and able to provide written informed consent. Participants will be screened for potential recruitment during their initial presentation to the hospital stroke services (see **section 2.5.1**). A participant information sheet will be provided explaining the study. Informed consent for screening will be taken at this point of contact for conducting the following assessments, 8 to 26 weeks after their stroke:

(i) assessment of cognition ("telephone-mini mental status examination")
(ii) assessment of function (modified Rankin scale)
(iii) blood test for fasting lipids, glucose, urea and electrolytes, and HbA1c.

If participants are eligible and interested, a participant information sheet, substudy information sheet (if taking part), and informant information sheet, along with a blood test form for fasting lipids, glucose, urea and electrolytes, and HbA1c will be given to them. (See **figure 4** for trial flow chart, see **Section 2.5.1** for details about recruitment).

Participants will be contacted a few days later to assess their views and answer questions about the trial. All participants and their informant will be booked to come to the research clinic and, if agreeable, for enrolment and randomisation into the study. In the research clinic the investigator will further explain the details of the trial and answer any questions that the participant has concerning trial participation.

The principal investigators and trial doctors, will decide if participants have the capacity to give consent at baseline by asking them the following series of questions to assess their understanding of the trial before taking consent.

This protocol is confidential and the property of the University of Nottingham. No part of it may be transmitted, reproduced, published, or used by others persons without prior written authorisation from the University of Nottingham

1. What is the trial aiming to achieve? (Answer: if intensive treatment of high blood pressure and lipids will prevent cognitive decline)
2. What are the two groups of intervention? (Answer: intensive and guideline)
3. How long will treatment be continued? (Answer: 1-8 years)

Potential participants who answer all the 3 questions correctly will be enrolled into the study. A signed and dated informed consent will be taken before the participant is recruited into the trial.

Informed consent will be collected from each participant before they undergo any interventions (including physical examination and history taking) related to the trial. Signed consent forms will be kept by the Participant and Investigator, and in the participant's hospital records. The GP will be informed if the participant agrees to join the trial.

As assessment of cognitive impairment is one of the objectives of the trial, it is inevitable that some participants will lose the capacity to maintain consent for the duration of their participation. This will be explained to potential participants. Consent will be taken at enrolment, to continue in the trial, should participants lose the capacity to maintain consent during the trial. However, if a participant has lost capacity and the participant's informant feels that continuing in the trial is not in the participant's best interests, the informant can withdraw the participant from the trial.

If needed, the usual hospital interpreter and translator services may be used to assist with discussion of the trial, the participant information sheets, and consent forms. But consent forms and information sheets will not be available printed in other languages since it will not be possible to do telephone or clinic outcome assessments in other languages. It will be explained to the potential participant that entry into the trial is entirely voluntary and that routine treatment and care will not be affected by their decision. It will also be explained that they can withdraw at any time but attempts will be made to avoid this occurrence. Withdrawal may comprise either withdrawal from treatment but with continuing follow-up, or withdrawal from both treatment and follow-up. In the event of withdrawal, it will be explained that existing data cannot be erased; consent to use this data in the final analyses will be sought, where appropriate.

Should there be any major amendments to the protocol that might affect the continued participation in the trial by a participant and/or informant, consent will be obtained using an amended Consent form approved by the Research Ethics Committee, which will be signed by the participant and/or informant.

### 2.5.5 *Informant (Consultee)*

Availability of an informant (partner, sibling, child, friend) for the participant is a key inclusion criterion in the trial, as informant questionnaires (IQCODE) can give vital information about the participant's cognition. If an informant can no longer fulfil their role (e.g. through death, or loss of capacity), then another informant will need to be consented. For this reason, two or more potential informants should be identified at baseline. It will be the aim to continue with a single informant as far as possible (see **figure 5).**

This protocol is confidential and the property of the University of Nottingham. No part of it may be transmitted, reproduced, published, or used by others persons without prior written authorisation from the University of Nottingham

**Figure 5** Algorithm for seeking consent from the participant and original informant, from one or more further informants if the earlier ones are no longer available, and from the participant and/or informant for major protocol changes.

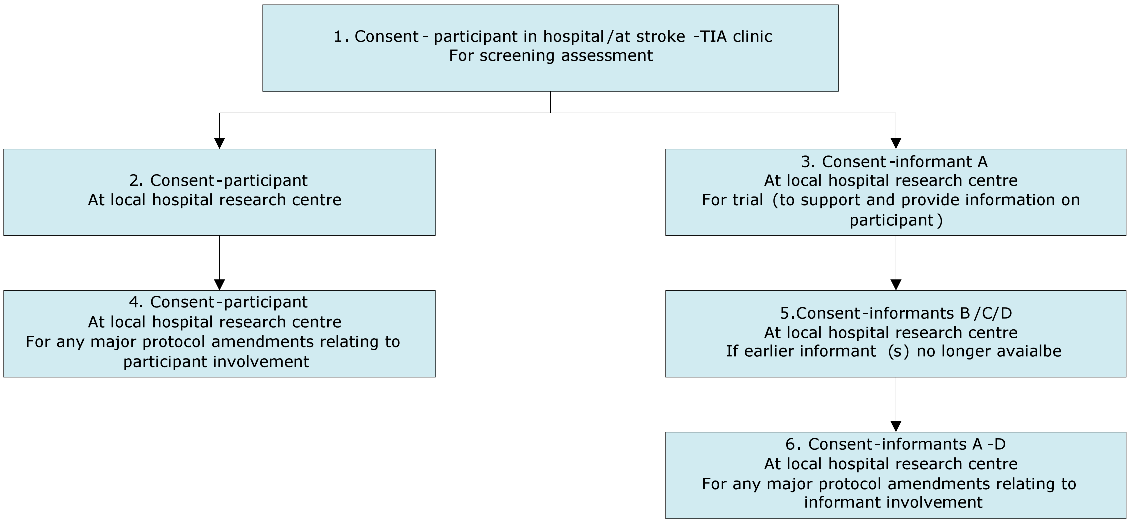


### 2.5.6 *Expected duration of participant participation*

Trial participation will range from 1- 8 years depending on the time of recruitment. Long follow-up is essential in trials of cognition since cognitive impairment may take many years to develop.

### 2.5.7 *Removal of participants from therapy or assessments*

Participants may leave the trial for a variety of reasons, as detailed below. It should be noted that abrupt termination of trial treatment could affect the participant's safety (e.g. hypertensive rebound) and administration of alternative treatment should be considered.

#### 2.5.7.1 *Withdrawal of consent*

Participation in the trial is voluntary and participants are free to withdraw from the trial at any stage without giving a reason. However, if a participant wishes to withdraw, they will be requested to at least permit primary outcome data to be collected, ideally at the end of the follow-up period, ensuring that enough data are recorded to support the planned analysis. Participants won't be accepted as lost to follow-up unless all attempted contacts have been fruitless, including: phone calls, letters, visits to their home, contact with their next of kin, and contact with their GP. Participants will be made aware (via the information sheet and consent form) that should they withdraw, the data collected up to the date of withdrawal cannot be erased and will be used in the final analysis. Participants who lose capacity during the trial may be withdrawn from the trial by their informant, if the informant feels that continued participation is not in the participant's best interests.

This protocol is confidential and the property of the University of Nottingham. No part of it may be transmitted, reproduced, published, or used by others persons without prior written authorisation from the University of Nottingham

### 2.5.7.2 ***Clinical need***

The participant's primary physician is not blinded to treatment allocation and may remove, change or add to treatment if they feel this is clinically indicated (e.g. for reasons of safety or new information becoming available on the trial medication or condition being treated).

### 2.5.7.3 ***Failure of participant to adhere to protocol requirements***

The Principal Investigator may remove the participant from the trial if they fail to adhere to the protocol through ***protocol violations*** and/or ***protocol deviations***, and will be reported to the Chief Investigator of the trial centre.

#### **2.5.7.3.1** ***Protocol Violation***

A **protocol violation** is a deviation from the trial protocol where a participant is enrolled in spite of not fulfilling all the inclusion and exclusion criteria, or where deviations from the protocol could affect the trial delivery or interpretation significantly.

The following baseline measures constitute a 'protocol violation':

- Participant<60 years of age
- "Telephone MMSE" score≤16
- "Telephone MMSE" score ≥21 if aged between 60-70
- No index stroke
- Randomisations <3 months or >7 months from onset of index stroke
- Failure to obtain consent of participant
- Participant with mRs >2
- Failure to identify haemorrhagic stroke
- Participant enrolled with *known* severe concomitant illness
- Participant enrolled with *known* intracranial pathology other than stroke
- Participant involved at time of randomisation in another medicinal and/or devices clinical trial
- No brain imaging during index stroke event
- No capacity to consent for the trial
- Failure to meet the systolic BP inclusion criteria
- Failure to meet the total cholesterol inclusion criteria
- Absence of an informant

The following practice during the trial constitutes a 'protocol violation':

- Participant never receives 'intensive' BP lowering therapy when randomised to do so.
- Participant never receives 'intensive' lipid lowering therapy when randomised to do so.
- Failure to complete SAEs where appropriate
- Annual clinic/telephone assessments are not performed.

These lists of protocol violations will be updated, as necessary, in a Working Practice Document which will be uploaded and available on the trial website.

This protocol is confidential and the property of the University of Nottingham. No part of it may be transmitted, reproduced, published, or used by others persons without prior written authorisation from the University of Nottingham

#### 2.5.7.3.2 *Protocol Deviation*

A ***protocol deviation*** is a minor deviation from the protocol that affects the conduct of the trial in a minor way. This includes any deviation from the trial protocol that is not listed as a protocol violation.

The following practice during the trial constitute a 'protocol deviation'

- Participant has no cranial imaging if they have another stroke.
- Clinic or telephone assessments done outside the specified time by more than 30 days. For the intensive BP group, clinic visits outside the specified time period by more than 14 days, or the time period between the visits is less than 2 weeks.
- Participant is not fully compliant with randomised treatment.

These lists of protocol deviations will be updated, as necessary, in a working practice document which will be uploaded and available on the trial website.

## 2.6 TRIAL TREATMENT AND REGIMEN

Study participants will be randomised to:

- Intensive or guideline BP lowering (all participants)
- Intensive or guideline lipid lowering (ischaemic stroke only)

As a result, patients can be randomised to one of 6 groups:

- Intensive BP lowering and intensive lipid lowering (ischaemic stroke only)
- Intensive BP lowering and guideline lipid lowering (ischaemic stroke only)
- Guideline BP lowering and intensive lipid lowering (ischaemic stroke only)
- Guideline BP lowering and guideline lipid lowering (ischaemic stroke only)
- Intensive BP lowering only (intracerebral haemorrhage only)
- Guideline BP lowering only (intracerebral haemorrhage only)

The trial will assess management strategies ('intensive' vs. 'guideline'), not particular drugs. All participants will receive lifestyle advice. Participants randomised to the guideline groups will be managed by their GP as per the current national/international guidelines and local practice. Participants in the intensive group will be managed by the local hospital research centre and medications initiated by either the local investigator or GP (following advice from the local investigator), and continued by the GP. The trial does not stipulate specific drugs but gives examples of these and relevant doses. The local hospital research centres and clinicians can use locally supported interventions as long as they fit with the overall design of the trial, i.e. intensive versus guideline BP and lipid lowering.

### *2.6.1 Follow up visits*

All participants will be followed up every six months at the local hospital research centre; a blood form for fasting lipids, glucose, urea and electrolytes, and HbA1c will be posted to the participants 2-3 weeks prior to each clinic visit. They will be advised to have the test done, at their GP practice, 1-2 weeks prior to the visit, to aid

This protocol is confidential and the property of the University of Nottingham. No part of it may be transmitted, reproduced, published, or used by others persons without prior written authorisation from the University of Nottingham

treatment decisions during the clinic visit. Cognition and other outcome data will be collected at the 6 month, 18 month, 30 month etc clinic visits (see **section 2.2, appendices A-J)**. Cognition data will not be collected at the 12 month, 24 month, 36 month clinic visits as all participants will also have telephone follow-up calls assessing cognition and dependency (see **section 2.2, appendices C,D,F,G,H,I,J,**) at 12 months and then annually. The index event ECG will be collected at the Baseline visit and an ECG will be taken at the 6 month, 18 month, 30 month etc clinic visits.

Participants in the intensive blood pressure group will have additional follow-up at one, two and three months after randomisation to monitor and modify treatment if necessary. These participants will be provided with a blood test form for U&E (urea and electrolytes) at: baseline, one month and two month visits, and advised to have the test at their local GP practice, 1-2 weeks prior to the next clinic visit. Rapid escalation and continuing intensive maintenance treatment is vital to ensure that a long-term difference in SBP of at least 10 mmHg is present between the treatment groups.

Participants in the intensive lipid-lowering group will have an additional follow-up at three months after randomisation to monitor and modify treatment if necessary. These participants will be provided with a blood test form for lipids at the baseline visit and advised to have the test done at their local GP practice, 1-2 weeks prior to their 3 month visit.

At formal research clinic appointments, if an intensive patient is found to have BP and/or lipid readings above the specified trial targets please bring them back to a 'floating' appointment. This should be at 1 month post clinic. The follow-up comprises assessment of the latest BP and/or lipid levels, current medications, any recent adverse events, and any new other medical history. Subject to these, treatment should be escalated.

The following data collected during clinic follow-up visits will be fed back to the GPs by the PODCAST ICC annually, as they also qualify as 'Quality and Outcomes Framework (QOF)' indicators: type of stroke, presence of myocardial infarction, angina, heart, failure, atrial fibrillation, dementia, depression, asthma or COPD (chronic obstructive pulmonary disease); BP, BMI (Body Mass Index), cholesterol levels, eGFR (estimated glomerular filtration rate); list of participant's medications such as antihypertensive medications, lipid lowering agents, antiplatelets and anticoagulants; smoking status, advice on smoking cessation and dietary changes. Prior consent will be taken from all participants to share this information with their GPs.

### *2.6.2 BP lowering arm*

The composition of antihypertensive agents will vary between participants since the drugs are often used for other indications (e.g. 'A'/'B' post MI) and have contraindications (e.g. avoid 'A' in bilateral renal artery stenosis, avoid 'B' in asthma). The aim is to maintain a difference in SBP >10 mmHg between the randomised treatment groups of 'intensive' versus 'guideline' BP management. All participants will receive advice on salt restriction.

This protocol is confidential and the property of the University of Nottingham. No part of it may be transmitted, reproduced, published, or used by others persons without prior written authorisation from the University of Nottingham

The following notes are only a guide and investigators may choose to differ, based on local policy, individual practice and patient specific characteristics.

Antihypertensive drugs will be chosen according to the NICE/BHS 'A (B)/CD' guideline (CG34) where:(20)

- A = angiotensin converting enzyme inhibitor (ACE-inhibitor, e.g. lisinopril 5-20 mg od, perindopril 2-8 mg od, ramipril 1.25-5 mg bd) or angiotensin receptor antagonist (ARA, e.g. losartan 25-100 mg od, candesartan 8-32 mg od)
- B = ß-receptor antagonist (e.g. atenolol 25-100 mg od, bisoprolol 5-20 mg od)
- C = calcium channel blocker (e.g. amlodipine 5-10 mg od, nifedipine LA 30-60 mg od, diltiazem, verapamil SR)
- D = diuretic (e.g. bendroflumethiazide 2.5 mg od, hydrochlorothiazide 12.5 mg od)

Participants should be started on either (provided there are no contraindications):

- An 'A' drug, with subsequent addition of a 'C' then 'D' drug (as required); or
- A 'C' drug, with subsequent addition of an 'A' then 'D' drug (as required)

Additional drugs may be added from other classes:

- Potassium sparing diuretics (e.g. spironolactone 12.5-100 mg od,(21) amiloride 5-20 mg od)
- α-receptor antagonists (e.g. doxazosin 4-16 mg od)
- Centrally acting drugs (e.g. moxonidine 200-600 μg daily in divided doses)
- 'B' drugs (e.g. atenolol 25-100 mg od)

Investigators may choose to increase the dose of existing drugs (although this can be associated with adverse events and only moderate further reductions in BP) or add drugs from additional classes. 'Long acting' drugs should be chosen in preference to those which need twice/thrice daily dosing.

The following advice will be updated as a 'Working Practice Document', on the trial website.

- Start drugs at medium, not high, dose. The dose should be increased 2-4 weeks later for additional BP effect although side effects become more prominent as doses tend to the maximum.
- Start with the lowest dose in very elderly patients or those with heart failure.
- Alternatives to the suggested drugs listed above may be used according to local practice and formulary availability.
- Consider escalating drug doses in between trial visits so as to accelerate control of blood pressure, i.e. write prescriptions with 2-4 weeks of one dose then with 2-4 weeks at the next dose up.
- Always treat clinical dehydration/hypovolaemia before adding drugs or increasing doses so as to avoid significant hypotension.
- If 'A' or 'K' drugs are added, check renal function (U&E/BUN) after 1 week.

This protocol is confidential and the property of the University of Nottingham. No part of it may be transmitted, reproduced, published, or used by others persons without prior written authorisation from the University of Nottingham

- If eGFR <45 (<37 in people of African/Afro-Caribbean origin) after addition of 'A', stop 'A' and use alternative strategy.
- If potassium >5.5 mmol/l after addition of 'A' or 'K', stop this and use alternative strategy.
- If sodium <130 mmol/l after addition of 'D', stop it and use alternative strategy.
- Specific drug classes may be indicated according to the presence of co-morbidities:
- Post myocardial infarction – consider 'A' and/or 'B'
- Diabetes mellitus – consider 'A'
- Specific drug classes are contra-indicated in the presence of known co-morbidities:
    - Asthma – avoid 'B'
    - Renal artery stenosis (bilateral if 2 kidneys, unilateral if 1 kidney) – avoid 'A'
    - Consider referring compliant patients with uncontrolled/partially controlled high BP (i.e. SBP>160 on 3 or more BP lowering agents) to a specialist Hypertension clinic for specific investigation of secondary causes.
    - If cough or angioedema develops on ACE-I, switch to angiotensin receptor antagonist (ARA), e.g. losartan.
    - If bronchospasm develops on 'B', switch to another drug class as per management algorithm.
    - Significant postural hypotension, which may be symptomatic, may occur if adding 'A' to 'D'.
    - Do not use rate limiting 'C' (verapamil) with 'B' (ß-RA).
- Only wean down drugs/doses because of symptoms, not because of BP levels.
- If uncertain, always check in the hospital/community/national drugs formulary regarding doses, indications and contra-indications.

#### 2.6.2.1 *'Intensive' BP treatment group*

The target is a systolic BP (SBP) of <125 mmHg. The intensive BP treatment algorithm (see **working practice document**), taking account of NICE guidelines relating to Stroke (CG68), Hypertension (CG34) and type 2 diabetes (CG66), will be provided to aid investigators in treatment decision-making so that target SBP of <125 mmHg may be achieved. The algorithm is only a guide and investigators can choose other medications depending on local policy and practice. It will be updated, as new information becomes available on BP management, as a working practice document and mounted on the trial website. Following on from the NICE/BHS A(B)/CD rule, it is likely that participants randomised to the intensive group will receive 3 or more drugs and that additional agents will include agents such as doxazosin, spironolactone etc. Drugs will be weaned down if participants develop symptomatic hypotension.

#### 2.6.2.2 *'Guideline' BP treatment group*

The target systolic BP for the 'guideline' BP group is <140 mmHg (NICE CG 34). Drug therapy will typically include an 'A' and/or 'D' agent.(22) Monitoring and treatment for this group will occur in general practice to reflect current community-based practice based on national/international guidelines.

#### 2.6.2.3 *Blood pressure measurement*

As a central aim of this trial is to ascertain the effect of lowering blood pressure immediately post stroke, it is vital that BP is measured in an accurate, reproducible,

This protocol is confidential and the property of the University of Nottingham. No part of it may be transmitted, reproduced, published, or used by others persons without prior written authorisation from the University of Nottingham

unbiased, and validated manner. Measurements made using routine ward/clinic mercury or aneroid sphygmomanometers, or most semi-automatic devices, are not sufficient in these respects.

All BP measurements should be performed using a validated automated blood pressure monitor, e.g. Omron 705CP or 705CP II. These devices have been validated by the British Hypertension Society,(23) in contrast to some other automated devices which have not been found to be accurate or reliable, and were used in the recent positive ASCOT hypertension trial involving 20,000 patients.(24) Baseline and follow-up systolic and diastolic blood pressure and heart rate data are taken (4 measurements taken in rapid succession) in the non-paretic arm with the participant sitting (3 readings) and standing (1 reading) entered on the baseline form. BP and heart rate readings should be printed out using the monitor printer and attached to the BP 'print-out' sheet. The times of last antihypertensive drug ingestion and BP measurement will be recorded on the clinic forms. Two BP monitors will be supplied to each centre and should only be used for participants in the PODCAST trial. BP monitors will be checked by staff from the PODCAST ICC during site visits; if broken or inaccurate, the monitor will be recalibrated or replaced.

Further information on intensive blood pressure management is given in a working practice document.

#### 2.6.2.4 *Ambulatory blood pressure monitoring (ABPM)*

In centres with the necessary ambulatory blood pressure monitoring equipment (e.g. SpaceLabs 90207), participants will have 24 hour ABPM (25) performed at recruitment and at all future scheduled clinic appointments. ABPM data will provide detailed information on:

- BP and heart rate (HR) levels on treatment
- BP and HR profile over 24 hours (peak and trough effects)
- BP and HR variation (standard deviation)

ABPM data will be printed out and faxed to the PODCAST International Coordinating Centre. Other haemodynamic variables are also related to stroke and recurrence and these will be derived from BP and HR:(26, 27)

- Pulse pressure (PP) = Systolic BP – diastolic BP
- Mean arterial pressure (MAP) = Diastolic BP + (PP / 3)
- Pulse pressure index (PPI) = PP / MAP
- Rate-pressure product (RPP) = Systolic BP x HR

Data will be analysed with adjustment for baseline measurements.

#### 2.6.2.5 *Treatment of sustained severe high BP*

If participants develop severe high BP (systolic BP >160 mmHg), treatment should be increased as per the BP algorithm.

This protocol is confidential and the property of the University of Nottingham. No part of it may be transmitted, reproduced, published, or used by others persons without prior written authorisation from the University of Nottingham

2.6.2.6 ***Treatment of sustained low/low normal BP***

If participants develop symptomatic hypotension, treatment should be weaned down as per the BP algorithm. This will normally involve stopping the last added drug (i.e. 'last in/first out').

### 2.6.3 *Lipid lowering arm (ischaemic stroke only)*

Lipid lowering agents will include statins and ezetimibe, e.g. as per UK NICE guidelines.(28-30) Only participants with an ischaemic stroke will be included in the lipid lowering arm since statins may be associated with intracerebral haemorrhage (31) due to mild antiplatelet properties. The aim is to maintain a difference in LDL-cholesterol >1.0 mmol/l between the treatment groups.

2.6.3.1 ***'Intensive' lipid treatment group***

The target is a LDL-cholesterol (LDL-c) of <1.4 mmol/l (or total cholesterol <3.1 mmol/l if LDL-cholesterol cannot be calculated, e.g. because of high triglyceride levels). Participants will receive repeat advice to take a plant stanol/sterol (as a spread or drink) as part of meals. The research clinic staff will monitor and prescribe medications using the intensive lipid treatment algorithm (see **working practice document**) as a guide and recommend to the general practitioner to continue treatment unless there is a medical reason to change it.

At the baseline research clinic, and unless the LDL-cholesterol is <1.4 mmol/l, participants should, ideally, be started on, or switched to, a 'high intensity' statin (e.g. atorvastatin ≥40 mg,(28, 32)). Ezetimibe (10 mg od (29)) may be added at subsequent clinics if the LDL-cholesterol >1.4 (or total cholesterol >3.1 mmol/l if LDL-cholesterol cannot be calculated). The algorithm will be updated, as new information becomes available on lipid management, as a working practice document and mounted on the trial website.

Rapid escalation and continuing intensive maintenance treatment is vital to ensure that a long-term difference in LDL-c of at least 1.0 mmol/l (or TC of at least 1.0 mmol/l) is present between the treatment groups. Drugs will be weaned down if participants develop symptoms.

2.6.3.2 ***'Guideline' lipid treatment group***

The target LDL-cholesterol for the 'guideline' lipid group is <3.0 mmol/l (or total cholesterol <5.0 mmol/l if LDL-cholesterol cannot be calculated). Participants will receive advice to take a plant stanol/sterol spread on bread at baseline. Drug therapy will typically comprise a 'guideline' statin, e.g. simvastatin range 10-40 mg on,(33) pravastatin 10-40 mg on, fluvastatin 10-80mg on - see NICE lipid guideline CG 67, 2008.(28) Monitoring and treatment for this group will occur in general practice to reflect current community-based practice based on national/international guidelines.

2.6.3.3 ***Lipid measurement***

Fasting lipids will be measured at an (provisionally) accredited Clinical Biochemistry laboratory proximal to the recruiting hospital and GP. Fasting should be performed overnight and measurements should be made at least 1 month after the last change

This protocol is confidential and the property of the University of Nottingham. No part of it may be transmitted, reproduced, published, or used by others persons without prior written authorisation from the University of Nottingham

in lipid lowering therapy. Lipid measurement will utilise standard techniques and comprise:

- Total cholesterol
- Triglyceride
- HDL cholesterol
- LDL cholesterol (calculated)

Further information on intensive lipid lowering is given in a working practice document.

### 2.6.4 *Monitoring interventions*

A member of the PODCAST ICC staff will monitor recorded BP and lipids in individual participants, unblinded to therapy, and suggest dose/drug escalation/weaning based on the BP/lipid algorithms to the local investigator/GP for the intensive BP and lipid groups. Their aim will be to ensure that BP/lipid levels are appropriate for the participant's randomisation. In addition, all participants randomised to the intensive BP and lipid groups will have regular central telephone reminders to reinforce treatment assignment.

The Trial Management Committee will monitor BP and lipid levels, and treatment crossovers, for each treatment group, i.e. unblinded to therapy. The TMC will report to the Trial Steering Committee at least 4 monthly on the magnitude of separation in BP and lipid levels between the treatment groups. The DMC will also report to TSC on their observations of separation in BP and lipid levels between the treatment groups. [Note: It is acceptable for trialists to un-blind themselves to surrogate outcomes such as BP to ensure that trial protocols are working, as done in HOT (34, 35)and MRC ENOS.(1)]

### *2.6.5 Other secondary vascular prophylaxis*

All participants with stroke should receive standard life style advice and rehabilitation (as per NICE CG 68, 2008),(36) including:

- Diet – calorie, salt, alcohol
- Exercise
- Smoking cessation
- Rehabilitation (e.g. physiotherapy, occupational therapy, speech & language therapy, as required
- Psychological assessment and therapy
- All participants with ischaemic stroke should receive standard secondary prophylaxis (as per NICE CG 68, 2008),(36) including:
- Oral anticoagulation, if a cardioembolic source of stroke is suspected
- Antiplatelet agents (e.g. combined aspirin 50-81 mg od and dipyridamole MR 200 mg bd)
- Carotid endarterectomy for ipsilateral severe internal carotid artery stenosis

This protocol is confidential and the property of the University of Nottingham. No part of it may be transmitted, reproduced, published, or used by others persons without prior written authorisation from the University of Nottingham

All concomitant treatments will be documented on the Case Report Form (CRF) and also in the participant's medical record, including any changes to these treatments.

### *2.6.6 Blood Biomarkers and Pharmacogenetics Substudy*

Tertiary questions in PODCAST include assessing the effects of the interventions on blood biomarkers, and by participant’s genotype. These blood measures are optional. Centres who wish to participate in the blood biomarker study should have appropriate storage facilities including access to a centrifuge and freezer.

Blood samples will be taken at baseline (4 ml into EDTA, 8 ml clotted). If it is not possible to take a blood sample at enrolment, both clotted (8 ml) and EDTA (4 ml) samples will be taken at the next feasible follow-up clinic visit. Clotted (serum) samples should be centrifuged prior to freezing; the EDTA samples should be frozen without centrifugation. Blood samples should be anonymised (identifiable by the centre number, participant trial number, participant initials, and date of sample) and stored locally in a freezer at -20$^{o}$C (or lower if possible at -60$^{o}$C to -80$^{o}$C) and accounted for using the Blood Sample Freezer Log. The PODCAST ICC at Nottingham will arrange transfer of blood samples to Nottingham UK, for analysis. Blood samples will be destroyed once analysis is completed, this being dependent on the trial’s completion date. Samples will not be sold to third parties.

#### 2.6.6.1 *Soluble markers of outcome and efficacy*

The exact identity of blood biomarkers will depend on developing knowledge on what may most usefully be measured. Examples include markers of vasomotor activity, lipid metabolism, thrombosis and inflammation.

#### 2.6.6.2 *Genetic studies*

The exact identity of genetic markers will depend on developing knowledge of what may most usefully be measured. Examples include genes related to Apo-E, mechanism of action of drugs, lipid metabolism, thrombosis and inflammation. However, genetic methodology is evolving rapidly and it is not possible presently to say what approaches will be sued.

The consent form will allow the participant to opt-in to the genetic substudy. Participants may continue in the overall trial, even if they elect not to consent to the genetics substudy. The participant may request destruction of the genetic samples at any time after consent and prior to creation of an anonymised database.

### 2.6.7 *Neuroimaging Substudy*

Cerebral white matter lesions (WML) have been associated with cognitive impairment in demented and non-demented elderly subjects. Whether lesion progression parallels this decline over time and whether treatment can modify this is less clear.

Separate funding is being sought to perform systematic neuro-imaging in a subset of participants. All participants will be invited to take part in the imaging sub study. All participants will have a base line scan (done as part of routine clinical care at or soon after the index stroke), and is an inclusion criteria for the study. Participants will have an additional scan, as part of the imaging substudy at the end of 3 years. An MRI scan

This protocol is confidential and the property of the University of Nottingham. No part of it may be transmitted, reproduced, published, or used by others persons without prior written authorisation from the University of Nottingham

of the brain will be the preferred imaging method for the additional scan, as it is more informative of cognitive change. However, where MRI cannot be performed, a CT scan of the brain will be done. A typical x-ray dose for a CT brain scan is 1.5 mSv, but due to variation in protocols, machines and patient size, this may reach 5mSv per scan.

The consent form will allow the participant to opt-in to the neuro-imaging substudy. Participants may continue in the overall trial, even if they elect not to consent to the neuro-imaging substudy.

# 3 STATISTICS

A medical statistician will support the TSC with analyses. An interim analysis will be done during the start-up phase to demonstrate feasibility of the trial, recruitment of centres and participants, whether sufficient on-treatment differences in BP and lipids are obtained and maintained, and whether cognition is being assessed satisfactorily. Interim analysis of cognitive measures and vascular events during the start-up phase will be blinded to treatment assignment.

## 3.1 Minimisation of bias

As the trial is based on management strategies, placebo-control is not relevant. Sources of bias will be minimised with:

- Central randomisation/concealment of allocation/data registration with real-time validation using an internet-based database
- Blinded telephone/clinic assessment of cognitive/vascular outcomes
- Blinded central adjudication of cognition/dementia and vascular events
- Assessment of participant recall of treatment groups ('intensive', 'standard') at end of trial
- Exclusion of participants enrolled in other drug trials
- Analysis by intention-to-treat with adjustment for stratification/minimisation factors, number of BP-lowering treatments and use of ezetimibe

## 3.2 Methods of analysis

### *3.2.1 Primary outcome*

Comparison of cognition (ACE-R extended to include death) between 'intensive' and 'guideline' BP/lipid lowering groups. The proportion of participants with cognitive impairment or who have died will be compared between the treatment groups, as done previously for MMSE (a subset of ACE-R).(33, 37)

Analyses will be adjusted for baseline stratification variables (see **section 2.3.1**) and minimisation variables (see **section 2.3.1**)

### *3.2.2 Analysis of cognition data*

Analyses based on binary outcomes are likely to be sub-optimal since dichotomisation of ordered categorical or continuous data is statistically inefficient, as seen in the

This protocol is confidential and the property of the University of Nottingham. No part of it may be transmitted, reproduced, published, or used by others persons without prior written authorisation from the University of Nottingham

'Optimising Analysis of Stroke Trials' collaboration for functional outcome after stroke.(38-40)

As a result, we are comparing, in the 'Optimising the analysis of cognition' collaboration (OA-Cog), ordinal and binary approaches using individual patient data from existing dementia and vascular trials where cognition was recorded; if this shows that ordinal approaches are statistically more efficient, we will change the analysis of cognition to use such an approach (see **figure 8**) illustrates how an ordered categorical scale may be created from cognition data.

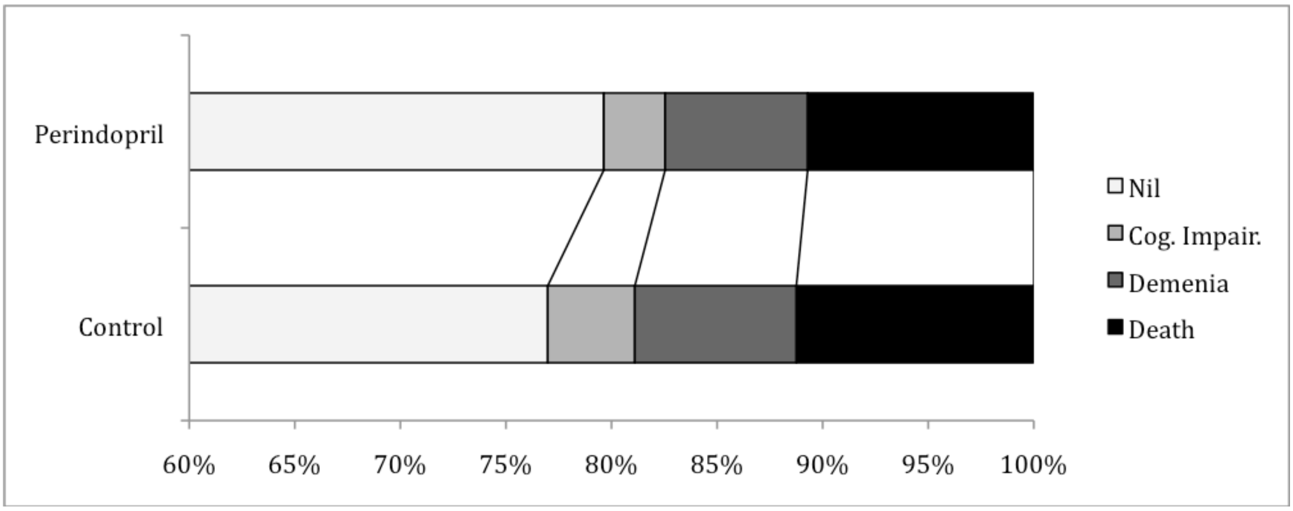


**Figure 8** Ordinal cognition scale using data from PROGRESS.(41, 42) 2000 patients without cognitive impairment (of the total ~3,300 patients) have been removed from each treatment group to make the illustration of cognition more clear. Perindopril-based BP lowering shifted patients from dementia/dead to no or some cognitive dysfunction (Mann-Whitney U, p=0.021, Bath P, unpublished).

Methods of analysing cognition vary considerably. The OA-Cog project will use existing BP/cholesterol-cognition trial data to optimise statistical approaches (as we did with stroke (38-40)) with comparison of:

- Gradient (42)
- Mean cognition (41, 43, 44)
- Median cognition
- Mean change in cognition (43, 45-48)
- Ordinal cognitive score (see **figure 8**)

Analysis of the primary outcome will use the optimum approach once this has been identified. Additionally, techniques will be compared for dealing with participants who die:

- Assign ACE-R score=-1
- Use last cognition score carried forward
- Calculate gradient of cognition scores,(42) assuming both linear and curvilinear models
- Create an ordered categorical scale from data on cognition, dementia and death (see **figure 8**)

This protocol is confidential and the property of the University of Nottingham. No part of it may be transmitted, reproduced, published, or used by others persons without prior written authorisation from the University of Nottingham

Dementia will be analysed as:

- Proportions (37)
- As part of an ordered categorical scale (see **figure 8**)

Differential dropouts will also be assessed.(49)
The final analysis will be described in detail in a statistical analysis plan, available on the trial website.

### 3.2.3 *Other outcomes*

Secondary and safety outcomes will be analysed using multiple regression, ordinal logistic regression or binary logistic regression, depending on the type of data. Where possible, dichotomous outcomes will be converted into ordinal outcomes (as in **figure 8**) Analyses will be adjusted for the covariates as listed in **section 2.3.1** since this approach increases statistical power (40) and is recommended by EMEA ('Points to consider').(50)

## 3.3 Sample size and justification

### 3.3.1 *Start-up phase*

Recruitment of 600 participants (300/BP group, ~270/statin group) will be sufficient to demonstrate adequacy in recruitment of centres and participants, whether sufficient on-treatment differences in BP and lipids can be obtained and maintained, and whether cognition can be assessed satisfactorily. No formal sample size calculation is relevant to this part of the trial.

### 3.3.2 *Main phase*

Currently, ACE-R will be analysed as combined cognitive impairment or death using logistic regression; however the intention is to change this to an approach which optimises statistical power, depending on the results of the OA-Cog study (as discussed in section **3.2.2**). The whole trial (start-up + main phases) will need a sample size of 3,400 (1,700 per group) post-stroke participants, assuming:

- Significance, α = 5%
- Power (1-ß) = 90%
- Rate of cognitive impairment or death in guideline' BP group = 25% at 5 years (main trial, average length of follow-up 4 years) [34]
- Rate of cognitive impairment or death in 'intensive' BP group = 20%, i.e. absolute risk reduction (ARR) = 5% (number-needed-to-treat = 20), relative risk reduction (RRR) = 20%
- Losses to follow-up = 3%

Hence, 765 participants (0.225 x 3,400) will need to develop cognitive impairment or die. The sample size allows a smaller but clinically worthwhile decline in cognitive decline to be identified with 80% power, i.e. ARR = 4.5% (RRR 18%). Since there are less existing data on the effect of cholesterol lowering on cognition, the statin factor

This protocol is confidential and the property of the University of Nottingham. No part of it may be transmitted, reproduced, published, or used by others persons without prior written authorisation from the University of Nottingham

will assume the same RRR (20%) but have less power (~86%) since it will only involve participants with ischaemic stroke (~3,060).

Changing from a binary to ordinal analysis of the primary outcome may allow for a reduction in sample size of up to 30%, as seen in the 'Optimising Analysis of Stroke Trials' collaboration for functional outcome after stroke.(38-40) Providing, ordinal analysis appears to be more efficient than binary analysis for cognition data, the trial will be re-sized according to the method of Whitehead.(51) Any such change will be performed prior to database lock, blinded to treatment, and defined explicitly in the Statistical Analysis Plan.

## 3.4 Definition of populations analysed

### *3.4.1 Safety Set*

All randomised participants.

### 3.4.2 *Full Analysis Set (FAS)*

All participants in the Safety Set, and who took at least one treatment dose, and for whom at least one post-baseline assessment of the primary endpoint (ACE-R and vital status) is available. Participants in the FAS will be defined prior to database lock.

### 3.4.3 *Per Protocol Set (PPS)*

All participants in the Full Analysis Set, and who are deemed to have no ***protocol violations*** (i.e. no severe deviations that might have interfered with the objectives of the trial). Participants in the PPS will be defined prior to database lock.

### 3.4.4 *Analyses*

Efficacy will be assessed using the ***Full Analysis Set***; secondary analyses will also assess efficacy in the ***Per Protocol Set***. Safety summaries will be performed on the ***Safety Set***. Major protocol deviations will lead to exclusion of a participant from the ***Per Protocol Set***.

## 3.5 Health economic analysis

The impact of 'intensive' BP and lipid lowering on quality of life will be assessed using the EuroQoL. A full health-economic analysis will be performed as part of the trial and will cover measurement of service use, including costs of dementia/cognitive impairment, costs of excess treatment, cost/event (cognitive decline) prevented and cost/QALY.

## 3.6 Potential analysis issues

### *3.6.1 Falling event rates*

Event rates are often seen to be falling and lower than expected in vascular prevention trials, this often requiring recruitment of more participants and/or

This protocol is confidential and the property of the University of Nottingham. No part of it may be transmitted, reproduced, published, or used by others persons without prior written authorisation from the University of Nottingham

prolongation of follow-up. The main issue in cognition/dementia studies is to ensure adequate length of follow-up, i.e. 5 years or more, so that cognitive impairment has time to develop. These issues will be monitored during the trial.

### *3.6.2 Adequate BP/lipid effects*

The only large intensity BP trial (HOT (34, 35)) did not achieve its target BP differences. The start-up phase will check that differences in BP/lipids can be maintained; Participants in the intensive BP/lipid lowering groups will receive reminders about treatment during each clinic and telephone follow-up. Secondary observational analyses will assess the relationship between individual changes in BP/lipids and cognition.

### *3.6.3 Guideline drift*

Guidelines may change over the life of the trial such that guideline BP and lipid targets could be reduced with time. In contrast, cost and participant resistance to taking multiple interventions may oppose this trend. The trial will monitor and adapt to such changes if detected.

### *3.6.4 Analysis of cognition*

Methods for analysing cognition vary considerably and those using binary approaches may be sub-optimal. We have set up an international collaboration using existing BP/cholesterol-cognition trial data to optimise statistical approaches, as discussed in section 3.2.2, which will improve statistical efficiency thereby allowing a reduction in sample size.

# 4 ADVERSE EVENTS

## 4.1 Definitions

### *4.1.1 Adverse Event*

An adverse event (AE) is defined as any unfavourable and unintended sign including an abnormal laboratory finding, symptom or disease associated with the use of a medical treatment or procedure, regardless of whether it is considered related to the medical treatment or procedure, that occurs during the course of the study.

### *4.1.2 Adverse reaction*

An adverse reaction (AR) is any untoward and unintended response in a participant to a drug, which is related to any dose administered to that participant. Serious Adverse Event (SAE) or Serious Adverse Reaction (SAR)

### *4.1.3 Serious Adverse Event (SAE) or Serious Adverse Reaction (SAR)*

Any adverse event or reaction occurring following trial-mandated procedures, having received BP and/or lipid lowering therapy, that results in any of the following outcomes:

This protocol is confidential and the property of the University of Nottingham. No part of it may be transmitted, reproduced, published, or used by others persons without prior written authorisation from the University of Nottingham

1. Death
2. A life-threatening adverse event
3. Inpatient hospitalisation or prolongation of existing hospitalisation
4. A disability / incapacity
5. A congenital anomaly in the offspring of a participant
6. Important medical events – these are events which are not fatal, life-threatening, or require hospitalisation, but nevertheless may jeopardise the participant and may require medical or surgical intervention to prevent one of the other outcomes listed above

#### *4.1.4 Suspected Unexpected Serious Adverse Reactions (SUSAR)*

SUSARs are serious adverse reactions, which are serious (as defined for SAEs), and unexpected (i.e. they are not recognised reactions for the trial medications).

#### 4.1.5 *Serious versus severe adverse events*

A distinction is drawn between serious and ***severe adverse events***. Severity is a measure of intensity whereas seriousness is defined using the criteria above. Hence, a severe adverse event need not necessarily be serious (e.g. most severe headaches are not serious).

### 4.2 Causality

The relationship between clinical events, including laboratory test abnormalities, and treatment will be assigned by the Investigator as follows:

#### 4.2.1 *Not related or improbable*

Clinical event, including laboratory test abnormality, with a temporal relationship to trial treatments which makes a causal relationship incompatible or for which other treatments, chemicals or disease provide a plausible explanation. This will be counted as 'unrelated' for analysis purposes.

#### 4.2.2 *Improbable*

Clinical events, including laboratory test abnormalities, with a temporal relationship to trial treatments which makes a causal relationship unlikely, or for which other treatments, chemicals or disease provide a plausible explanation. This will be counted as 'unrelated' for analysis purposes.

#### 4.2.3 *Possible*

Clinical events, including laboratory test abnormalities, with a temporal relationship to trial treatments which makes a causal relationship a reasonable possibility, but which could also be explained by other treatments, chemicals or concurrent disease. This will be counted as 'unrelated' for analysis purposes.

This protocol is confidential and the property of the University of Nottingham. No part of it may be transmitted, reproduced, published, or used by others persons without prior written authorisation from the University of Nottingham

#### 4.2.4 *Probable*

Clinical events, including laboratory test abnormalities, with a temporal relationship to trial treatments, which makes a causal relationship a reasonable possibility, and is unlikely to be due to other treatments, chemicals or concurrent disease. This will be counted as 'related' for analysis purposes.

#### 4.2.5 *Definite*

Clinical events, including laboratory test abnormalities, with a temporal relationship to trial treatment administration which makes a causal relationship a reasonable possibility, and which can definitely not be attributed to other causes. This will be counted as 'related' for analysis purposes.

### 4.3 Recording and Safety Reporting

#### *4.3.1 Adverse events*

AEs will not be recorded or reported due to their high incidence in stroke patients.

#### *4.3.2 Adverse Reactions*

Medically important ARs listed in the British National Formulary for antihypertensive and lipid lowering drugs will be recorded in the trial database, but not reported to regulatory authorities. It is important to record these ARs, since they will influence blood pressure and/or lipid management strategies as per the guiding algorithms.

#### *4.3.3 Serious Adverse Events (SAEs) related to Stroke*

Stroke and developing cognitive impairment are conditions with high morbidity and mortality, and several adverse events may occur during a patient's participation in the trial. SAE'S that can be expected after stroke will be recorded in the trial database but not reported to regulatory authorities. A list is provided in a working practice document on the trial website. This list is a guide, and will be updated through the working practice document on the trial website.. Since most medical conditions can be described using a variety of descriptors, investigators should try, where possible, to match up SAE titles with the list below.

#### *4.3.4 Serious Adverse Reactions (SARs)*

As the trial is testing management strategies, not individual drugs, adverse reactions that are serious will be recorded on the trial database, but not reported to the regulatory authorities.

#### *4.3.5 Suspected Unexpected Serious Adverse Reactions (SUSAR)*

As the trial is testing management strategies, not individual drugs, and due to the long established nature of these drugs, SUSARs are not collected and recorded specifically, except as part of the recording of serious adverse reactions. However investigators are free to report adverse reactions/serious adverse reactions to national

This protocol is confidential and the property of the University of Nottingham. No part of it may be transmitted, reproduced, published, or used by others persons without prior written authorisation from the University of Nottingham

agencies as they wish, e.g. through the Commission of Human Medicines Yellow Card pathway (www.yellowcard.gov.uk) in the UK.

### 4.4 Serious Adverse Event (SAE) adjudication

All SAEs will be recorded and monitored until resolution, stabilisation, or until it has been shown that the trial treatment is not the cause. Such SAEs should be completed within one week of investigators being aware of the event. Likely causality will be entered.

For SAEs, the Chief Investigator and SAE adjudicator(s) shall:

- Assess the event for seriousness, expectedness and relatedness to the trial treatment
- Take appropriate medical action, which may include halting the trial and inform the Sponsor of such action
- Make any amendments as required to the trial protocol and inform the REC as required

### 4.5 Participant removal from the trial due to adverse events

Any participant who experiences an AR or SAR may be withdrawn from treatment at the discretion of the Principal Investigator, or at the request of the participant. However there are usually alternative treatments for reducing blood pressure and lipids, which may be used instead of a particular drug causing an AR/SAR. Hence it should usually be possible to avoid withdrawing a participant from treatment. If patients do withdraw from treatment, ideally they should stay in the trial for the purposes of follow up.

## 5 TRIAL MANAGEMENT

### 5.1 Sponsor

The University of Nottingham is the trial sponsor in the UK and will delegate responsibility for design and conduct of the trial to the Chief Investigator via our Sponsor/Chief Investigator agreement. The sponsor contact details are

Mr Paul Cartledge
Head of Research Grants and Contracts
Research Innovation Services
King's Meadow Campus, Lenton Lane
Nottingham, NG7 2NR
UK

### 5.2 Coordinating Centre

The Stroke Trials Unit (STU), part of the University of Nottingham's Clinical Trials Unit (which has provisional registration), will co-ordinate the trial. STU will have overall

This protocol is confidential and the property of the University of Nottingham. No part of it may be transmitted, reproduced, published, or used by others persons without prior written authorisation from the University of Nottingham

responsibility for the conduct of the trial and will be responsible for provision of trial materials, collation and analysis of data and reporting of the final results. They will act as the International Coordinating Centre, UK National Coordinating Centre, the primary point of contact for UK centres, and the secondary point of contact for non-UK centres.

Stroke Trials Unit
Division of Stroke Medicine
University of Nottingham
Clinical Science Building
City Hospital campus
Nottingham, NG5 1PBUK
Tel: +44 115 8231671
Fax: +44 115 8230273

## 5.3 Trial Steering Committee (TSC)

The TSC will provide overall supervision, as per their charter, and ensure that the trial is conducted in accordance with the principles of the ICH GCP and the relevant regulations. Any amendments to the trial will be agreed by the TSC. The TSC will provide advice to the investigators on all aspects of the trial. The composition of the TSC is given on the Trial website.

## 5.4 Data Monitoring Committee (DMC)

The Data Monitoring Committee (DMC) will monitor efficacy and safety as per their charter. As well as outcome measures, the DMC will also review recruitment, baseline data, balance in baseline factors between the treatment group, completeness of data, compliance to treatment, co-administered treatments, and outcome by sub groups. They will also review all serious adverse events (both adjudicated and unadjudicated) and protocol violations. The DMC will usually meet at least yearly by teleconference; the chairman will receive 6 monthly updates from the statistician. The composition of the DMC is given on the Trial website.

The Data Monitoring Committee charter will use similar stopping rules to those agreed and used in the MRC ENOS trial. (see **section 6.6**))

## 5.5 Outcome and event adjudication committees

There will be 3 adjudication committees:

- For cognitive decline and dementia
- For stroke and other vascular events
- For SAEs which do not relate to cognition of vascular events

The committees will follow their respective charters.

This protocol is confidential and the property of the University of Nottingham. No part of it may be transmitted, reproduced, published, or used by others persons without prior written authorisation from the University of Nottingham

# 6 ETHICAL AND REGULATORY ASPECTS

## 6.1 Ethics Committee and regulatory approvals

The trial will not be initiated before the protocol, informed consent forms, and participant and GP information sheets have received approval / favourable opinion from the UK Research Ethics Committee (REC), and the respective National Health Service (NHS) Research & Development (R&D) department. Should a protocol amendment be made that requires REC approval, the changes in the protocol will not be instituted until the amendment and revised informed consent forms and participant information sheets have been reviewed and received approval/favourable opinion from the REC and R&D departments. A protocol amendment intended to eliminate an apparent immediate hazard to participants may be implemented immediately providing that the REC are notified as soon as possible and an approval is requested. Minor protocol amendments only for logistical or administrative changes may be implemented immediately; and the REC will be informed.

The trial will be conducted in accordance with the ethical principles that have their origin in the Declaration of Helsinki, 1996; the principles of Good Clinical Practice (GCP), and the UK Department of Health Research Governance Framework for Health and Social care, 2005.

The trial is supported by NIHR (National Institute of Health Research) Stroke Research Network, NIHR Primary Care Research Network and NIHR Dementia and Neurodegenerative Diseases Research Network.

## 6.2 Informed consent and participant information

The process for obtaining participant informed consent will be in accordance with REC guidance, GCP, and any other regulatory requirements that might be introduced. The investigator or their nominee and the participant shall both sign and date the Informed Consent Form before the person can participate in the trial.

The participant will receive a copy of the signed and dated forms and the original will be retained in the Trial Master File. A second copy will be filed in the participant's medical notes and a signed and dated note made in the hospital notes that informed consent was obtained for the trial.

The decision regarding participation in the trial is entirely voluntary. The investigator or their nominee shall emphasise to them that consent regarding trial participation may be withdrawn at any time without penalty or affecting the quality or quantity of their future medical care, or loss of benefits to which the participant is otherwise entitled. No trial-specific interventions will be done before informed consent has been obtained.

If the Informed Consent Form is amended during the trial, the investigator shall follow all applicable regulatory requirements pertaining to approval of the amended Informed Consent Form by the REC and use of the amended form (including for ongoing participants).

This protocol is confidential and the property of the University of Nottingham. No part of it may be transmitted, reproduced, published, or used by others persons without prior written authorisation from the University of Nottingham

## 6.3 Records

### *6.3.1 Case Report Form (CRF)*

Each participant will be assigned a trial identity code number, allocated at randomisation, for use on CRFs, other trial documents, and the electronic database. The documents and database will also use their initials (of first and last names separated by a hyphen or middle name initial when available) and age.

CRFs will be treated as confidential documents and held securely in accordance with regulations. The investigator will make a separate confidential record, in a separate participant database, of the: participant's name, date of birth, local hospital number or NHS number, address, telephone number, relative/friend's contact details, and Participant Trial Number, to permit identification of all participants enrolled in the trial, so that follow-up may be performed. CRF access shall be restricted to those personnel approved by the Chief or local Principal Investigator and recorded on the 'Trial Delegation Log'.

All paper forms shall be filled in using black ballpoint pen. Errors shall be lined out, but not obliterated with correction fluid, and the correction inserted, initialled and dated. The Chief or Principal Investigator, or designate, shall sign a declaration ensuring accuracy of data recorded in the electronic-CRF through signing off database forms by the use of their Postal Index Number (PIN) code.

### 6.3.2 *Source documents*

Source documents shall be filed at the investigator's site and may include, but are not limited to, consent forms, current medical records, laboratory results, and pharmacy records. A CRF may also completely serve as its own source data. Only trial staff as listed on the Delegation Log shall have access to trial documentation other than the regulatory requirements listed below.

### 6.3.3 *Scan Transfer and Storage*

- Baseline and subsequent clinical or research CT and/or MR brain scans should be sent electronically (ideally) using the secure internet webload facility provided on the PODCAST website (www.podcast-trial.org/). Scans should not be anonymised prior to upload as certain fields such as study date, birth date and sex are essential to ensure that the scan is matched to the patient. The upload facility will transfer data using RC4-MD5 (128 bit) cipher encryption and anonymise the DICOM header of the images automatically. The DICOM header attributes that are anonymised are a subset of those specified in the 'Basic Application Level Confidentiality Profile' of the DICOM standard 3.15; namely the institution name, institution address, referring physician, referring physician's address, patient name, patient identifier, date of birth, other patient id, other patient names and patient's address attributes.
- If centres are unable to use the web upload facility, non anonymised scans can be copied on a CD/DVD with the data encrypted. The encrypted CD/DVD should be sent via recorded delivery to the PODCAST ICC. The password should be

This protocol is confidential and the property of the University of Nottingham. No part of it may be transmitted, reproduced, published, or used by others persons without prior written authorisation from the University of Nottingham

communicated separately via email. The data will be unencrypted at the PODCAST ICC and uploaded to the database as described previously (see above)

- If centres are unable to send the scans by the above methods, they will be advised to contact the PODCAST ICC, who will help them with the process.
- Under exceptional circumstances, for centres where the only method of transferring images is by films/hardcopies, centres will be advised to send non anonysmised films (this is essential as the co-ordinating centre can ensure that the scans can be checked against patient details) via recorded delivery. These will be digitised and the resulting data anonymised.
- All digital brain image data will be stored on secure computer servers owned and maintained by the Information Services, University of Nottingham, with access restricted both physically (locked server rooms) and by password. Access for adjudication, analysis and archiving will be by password.
- Anonymised imaging data shall be adjudicated by trained neuroradiologists who may be based at the Coordinating Centre or elsewhere.
- The systems have been designed to ensure the highest levels of data security and participant confidentiality, and will be further enhanced if future technological advances permit it. The enhancements to the current system may include the use of e-Science and Grid technologies (e.g. NeuroGrid, www.neurogrid.ac.uk/) if they prove to be superior to current systems.

### 6.3.4 *Direct access to source data and documents*

The CRF and all source documents, including progress notes and copies of laboratory and medical test results, shall made be available at all times for review by the Chief Investigator, PODCAST staff, Sponsor's designee and inspection by relevant regulatory authorities.

## 6.4 Data protection

All trial staff and investigators will endeavour to protect the rights of the trial's participants to privacy and informed consent, and will adhere to the UK Data Protection Act (1998). The CRF will only collect the minimum required information for the purposes of the trial. CRFs will be held securely, in a locked room, or locked cupboard or cabinet. Access to the information will be limited to the trial staff and investigators and relevant regulatory authorities (see above). Computer held data including the trial database will be held securely and password protected. All data will be stored on a secure dedicated web server. Access will be restricted by user identifiers, passwords and PINs (encrypted using a one way encryption method).

Personal information (e.g. name and address of participants and secondary contacts) about trial participants will be held at local centres and will be passed onto the National Coordinating Centre and International Coordinating Centre (Nottingham UK). Participant information will be held on a database at the ICC but will be separated from all clinical information; the latter remain anonymous (identifiable only by initials, trial number and age). Computer data will be backed up regularly to an offsite secure repository (to enable disaster recovery). Personal participant information will be used only for the purposes of the PODCAST trial and will not be passed on to third parties. The personal participant information will be deleted within 12 months of the end of the trial.

This protocol is confidential and the property of the University of Nottingham. No part of it may be transmitted, reproduced, published, or used by others persons without prior written authorisation from the University of Nottingham

Where permissible, the PODCAST ICC may use central databases to obtain additional follow-up information on participants enrolled into the trial. In the UK, this will involve use of the NHS Medical Research Information Service, Office of National Statistics (ONS) database. When information will be gathered on participants in this way, it will be clearly stated in the country specific patient/informant information sheets.

Information about the trial in the participant's medical records / hospital notes will be treated confidentially in the same way as all other confidential medical information.

## 6.5 Quality assurance and audit

### 6.5.1 *Insurance and indemnity*

Insurance and indemnity for trial participants and local trial staff is covered within the UK NHS Indemnity Arrangements for clinical negligence claims in the NHS, issued under cover of HSG (96) 48.(52) There are no special compensation arrangements, but trial participants may have recourse through the NHS complaints procedures.

The University of Nottingham has taken out an insurance policy to provide indemnity in the event of a successful litigious claim for proven non-negligent harm.

### 6.5.2 *Trial conduct*

Trial conduct will be subject to systems audit of the Trial Master File for inclusion of essential documents:

- Permissions to conduct the trial
- Trial Delegation Log
- CVs of trial staff and training received
- Local document control procedures
- Consent procedures and recruitment logs
- Adherence to procedures defined in the protocol (e.g. inclusion / exclusion criteria, correct randomisation, timeliness of visits)
- Serious Adverse Event recording and reporting; accountability of trial materials and equipment calibration logs

The Trial Coordinator, or where required, a nominated designee of the Sponsor, shall carry out a site systems audit, at least yearly, and an audit report shall be made to the Chief Investigator.

### 6.5.3 *Trial data*

Monitoring of trial data shall include:

- Confirmation of informed consent – for all participants
- Source data verification – use ROUNDUP SQR for calculating number of participants whose documents need to be monitored at centre (since last monitoring)
- Data storage and data transfer procedures
- Local quality control checks and procedures

This protocol is confidential and the property of the University of Nottingham. No part of it may be transmitted, reproduced, published, or used by others persons without prior written authorisation from the University of Nottingham

- Back-up and disaster recovery of any local databases and validation of data manipulation

The Trial Coordinator, or where required, a nominated designee of the Sponsor, shall carry out monitoring of trial data as an ongoing activity.

Entries on CRFs will be verified by inspection against the source data. A sample of CRFs [ROUNDUP SQR (number of participants at centre since last monitoring)] will be checked on a regular basis for verification of all entries made. In addition, the subsequent capture of data on the trial database will be checked. Where corrections are required these will carry a full audit trail and justification.

Trial data and evidence of monitoring and systems audits will be made available for inspection by REC as required.

### 6.5.4 *Record retention and archiving*

In compliance with the ICH/GCP guidelines, regulations and in accordance with the University of Nottingham's Research Code of Conduct, the Chief or local Principal Investigator will maintain all records and documents regarding the conduct of the trial. These will be retained for at least 7 years after the end of the trial, or for longer if required. If the responsible investigator is no longer able to maintain the trial records, a second person will be nominated to take over this responsibility.

The Trial Master File and trial documents held by the Chief Investigator on behalf of the Sponsor shall be finally archived at secure archive facilities at the University of Nottingham. This archive shall include all trial databases and associated meta-data encryption codes.

## 6.6 Discontinuation of the trial by the sponsor

The Sponsor reserves the right to discontinue this trial at any time for failure to meet expected enrolment goals, for safety or any other administrative reasons. The Sponsor shall take advice from the Trial Steering Committee, Data Monitoring Committee, and funder(s) as appropriate in making this decision.

We will use a similar Data Monitoring Committee charter for electively stopping the trial that is agreed for the MRC ENOS trial. This states that:

"During the period of recruitment into the study, the trial statistician will perform interim analyses on major outcome events and supply these, in strict confidence, to the members of the Data Monitoring Committee, along with any other analyses that the committee may request. In the light of these analyses, the Data Monitoring Committee will advise the Chairman of the Steering Committee and Chief Investigator if, in their view, the randomised comparisons in the trial have provided both:

a. "Proof beyond reasonable doubt"† that for all, or for some, specific types of patient, treatment is clearly indicated or clearly contraindicated in terms of the primary outcome measure, and

This protocol is confidential and the property of the University of Nottingham. No part of it may be transmitted, reproduced, published, or used by others persons without prior written authorisation from the University of Nottingham

b. Evidence that might reasonably be expected to influence materially the patient management of the many clinicians who are already aware of the results of any other relevant trials.

The Steering Committee can then decide whether to modify intake to the trial (or to seek extra data). Unless this happens, however, the Steering Committee, the collaborators, and the central administrative staff (except those who produce the confidential analyses) will remain ignorant of the interim results.

Collaborators, and all others associated with the trial, may write through the PODCAST office, Nottingham to the Chairman of the Data Monitoring Committee, drawing attention to any worries they may have about particular categories of patient requiring special consideration, or about any other matters that may be relevant.

†Appropriate criteria of proof beyond reasonable doubt cannot be specified precisely, but a common view is that a difference of at least 3 standard deviations in an interim analysis of a major outcome event may be needed to justify halting, or modifying, such a study prematurely. If this criterion were to be adopted, it would have the practical advantage that the exact number of interim analyses would be of little importance, and so no fixed schedule is proposed.

If a trial is discontinued for any of the above reasons, participants will go back to receiving standard care from their GPs.

## 6.7 Statement of confidentiality

Individual participant medical information obtained as a result of this trial is considered confidential and disclosure to third parties is prohibited with the exceptions noted above.

Participant confidentiality will be further ensured by utilising identification code numbers to correspond to treatment data in the computer files.

Such medical information may be given to the participant's medical team and all appropriate medical personnel responsible for the participant's welfare.

Data generated as a result of this trial will be available for inspection on request by the participating physicians, the University of Nottingham representatives, the REC, local R&D Departments and the regulatory authorities.

## 6.8 Publication and dissemination policy

Data and results will be shared as follows:

### 6.8.1 *Presentation*

The main trial results will be presented to the investigators, and to funding bodies, and at major international and national scientific meetings, in the name of the trial and investigators i.e. 'PODCAST Investigators'.

This protocol is confidential and the property of the University of Nottingham. No part of it may be transmitted, reproduced, published, or used by others persons without prior written authorisation from the University of Nottingham

#### 6.8.2 *Publication*

The main results from the trial will be written by a 'Writing Committee' and published in quality peer-reviewed journal(s) in the name of the investigators, i.e. PODCAST Investigators.

Secondary publications will be published as 'Person(s), for the PODCAST Investigators', where the person(s) are those who conceived, designed, or wrote the paper, or analysed and/or interpreted the data for the publication.

Abstracts will be presented as 'PODCAST Investigators, person(s)', where the person(s) act as a contact point for the trial.

Local investigators may present or publish data relating to their centre once the main trial findings have been published and following agreement by the Trial Steering Committee.

#### 6.8.3 *Sharing of data*

Anonymised subsets of data may be shared with other research groups and projects (e.g. Cochrane Collaboration, OA-Cog) once the main trial findings have been published, and following agreement by the Trial Steering Committee.

#### 6.8.4 *Management of post-trial BP and lipids*

Widespread presentation and publication of the results will allow participants and their general practitioners to discuss the most appropriate management for future control of BP and lipids.

### 6.9 User and public involvement

The trial has been reviewed, and is supported, by:

- Alzheimer's Society Quality Research in Dementia Consumer Advisory Network
- UK Stroke Research Network Prevention Clinical Studies Group
- Trent Stroke Consumer Group

Several Participants/Carer Public Involvement (PCPI) representatives are on the Trial Steering Committee (see www.podcast-trial.org/).

## 7 TRIAL FINANCES

### 7.1 Funding sources

The start-up phase is jointly funded by The Stroke Association UK and Alzheimer's Society UK. Funding for the main phase will be sought mid-way through the start-up phase subject to the trial being considered feasible by the Trial Steering Committee and the Data Monitoring Committee.

This protocol is confidential and the property of the University of Nottingham. No part of it may be transmitted, reproduced, published, or used by others persons without prior written authorisation from the University of Nottingham

The excess treatment costs and service support costs related to prescriptions and blood tests have been derived by a multidisciplinary team (including a finance officer) involving representatives from the Trent CLRN (Comprehensive Local Research Network), Nottingham University Hospitals NHS Trust, Nottingham PCTs, The University of Nottingham, NIHR Stroke Research Network (through the Trent Local Research Network) and NIHR Primary Care Reserch Network. These were then submitted to the Department of Health for confirmation. The costing template is available to participating sites on the document repository of the NIHR CSP ReDa (National Institute for Health Research Coordinated System for obtaining NHS Permission Research Database)

The excess treatment costs are part of government given PCT budgets and will be funded by the local Primary Care Trusts. The service support costs will be available through local CRLNs.

## 7.2 Participant stipends and payments

Participants will not be paid to participate in the trial. Travel or mileage/parking expenses will be offered for hospital visits.

This protocol is confidential and the property of the University of Nottingham. No part of it may be transmitted, reproduced, published, or used by others persons without prior written authorisation from the University of Nottingham

# 8 SIGNATURE PAGES

Signatories to Protocol:

Chief Investigator: Professor Philip Bath

Signature: ___________________________________

Date: ___________

This protocol is confidential and the property of the University of Nottingham. No part of it may be transmitted, reproduced, published, or used by others persons without prior written authorisation from the University of Nottingham

# APPENDICES

## Appendix A. Addenbrooke's Cognitive Examination-Revised (ACE-R)

The ACE-R will be modified to include death (thereby mimicking modification of functional outcome, e.g. Rankin Scale, to include death); participants who die will be assigned an ACE-R score of -1.

ADDENBROOKE'S COGNITIVE EXAMINATION – ACE-R

| | |
|---|---|
| Name : <br> Date of birth : <br> Hospital no. : <br><br> Addressograph | Date of testing: ____/___/___ <br> Tester's name:__________________________ <br> Age at leaving full-time education:________ <br> Occupation:____________________________ <br> Handedness:___________________________ |

ORIENTATION

| | | | | | | |
|---|---|---|---|---|---|---|
| Ask: What is the | Day ________ | Date ________ | Month ________ | Year ________ | Season ________ | [Score0-5] |
| Ask: Which | Building ________ | Floor ________ | Town ________ | County ________ | Country ________ | [Score0-5] <br> A + O |

REGISTRATION

Tell: 'I'm going to give you three words and I'd like you to repeat after me: lemon, key and ball'. After subject repeats, say ' Try to remember them because I'm going to ask you later'. Score only the first trial (repeat 3 times if necessary).
Register number of trials ____

[Score0-3]
A + O

ATTENTION & CONCENTRATION

Ask the subject: 'could you take seven away from a hundred? After the subject responds, ask him or her to take away another 7 to a total of 5 subtractions. If subject makes mistake, carry on and check subsequent answerS (i.e 93,84,77,70,63- score 4)
Stop after five subtractions (93, 86, 79, 72, 65). ____ ____ ____ ____ ____
Ask: 'could you please spell WORLD for me? Then ask him/her to spell it backwards:
____ ____ ____ ____ ____

[Score0-5]
(for best performed task)
A + O

MEMORY- Recall

Ask: 'Which 3 words did I ask you to repeat and remember?'
__________ ________ ___________

[Score0-3]
Memory

MEMORY– Anterograde Memory

Tell: ' I'm going to give you a name and address and I'd like you to repeat after me. We'll be doing that 3 times, so you have a chance to learn it because I'll be asking you later' Score only the third trial

[Score0 7]
Memory

| | 1st Trial | 2nd Trial | 3rd Trial |
|---|---|---|---|
| Harry Barnes | _ _ | _ _ | _ _ |
| 73 Orchard Close | _ _ _ | _ _ _ | _ _ _ |
| Kingsbridge | _ | _ | _ |
| Devon | _ | _ | _ |

MEMORY Retrograde Memory

This protocol is confidential and the property of the University of Nottingham. No part of it may be transmitted, reproduced, published, or used by others persons without prior written authorisation from the University of Nottingham

| | |
|---|---|
| Name of current Prime Minister<br>Name of the woman who was Prime Minister<br>Name of the USA president<br>Name of the USA president who was assassinated in the 1960's | [Score0-4]<br>☐<br>Memory |

| VERBAL FLUENCY - Letter 'P' and animals | | | | | |
|---|---|---|---|---|---|
| Letters<br>Say: 'I'm going to give you a letter of the alphabet and I'd like you to generate as many words as you can beginning with that letter, but not names of people or places. Are you ready? You've got a minute for that and the letter is letter P' | | | | [Score0 7]<br>☐Fluen cy | |
| | | | | >17 | 7 |
| | | | | 14-17 | 6 |
| | | | | 11-13 | 5 |
| | | | | 8-10 | 4 |
| | | | | 6-7 | 3 |
| | | | | 4-5 | 2 |
| | | | | 3-4 | 1 |
| | | | | <3 | 0 |
| | | | | total | |
| Animals<br>Say: 'Now let's change. I'd like you to generate as many animals as possible, any kind of animal, beginning with any letter, it doesn't matter'. | | | | [Score0 7]<br>☐<br>Fluency | |
| | | | | >21 | 7 |
| | | | | 17-21 | 6 |
| | | | | 14-16 | 5 |
| | | | | 11-13 | 4 |
| | | | | 9-10 | 3 |
| | | | | 7-8 | 2 |
| | | | | 5-6 | 1 |
| | | | | <5 | 0 |
| | | | | total | |

| L A N G U A G E - Comprehension | |
|---|---|
| Show written instruction: | [Score0-1]<br>☐<br>Language |

# Close your eyes

| | |
|---|---|
| 3 stage command:<br>'Take the paper in your right hand. Fold the paper in half. Put the paper on the floor' | [Score0-3]☐<br>Language |

| L A N G U A G E - Writing | |
|---|---|
| Ask the subject to make up a sentence and write it in the space below:<br>Score 1 if sentence contains a subject and a verb (see guide for examples) | [Score0-1]<br>☐<br>Language |

This protocol is confidential and the property of the University of Nottingham. No part of it may be transmitted, reproduced, published, or used by others persons without prior written authorisation from the University of Nottingham

| | |
|---|---|
| L A N G U A G E - Repetition | |
| Ask the subject to repeat:' hippopotamus'; 'eccentricity; 'unintelligible'; 'statistician'<br>Score 2 if all correct; 1 if 3 correct; 0 if 2 or less. | [Score0-2] |
| Ask the subject to repeat: 'Above, beyond and below' | [Score 0-1] |
| Ask the subject to repeat: 'No ifs, ands or buts' | [Score 0-1]<br>Language |
| L A N G U A G E - Naming | |
| Ask the subject to name the following pictures<br>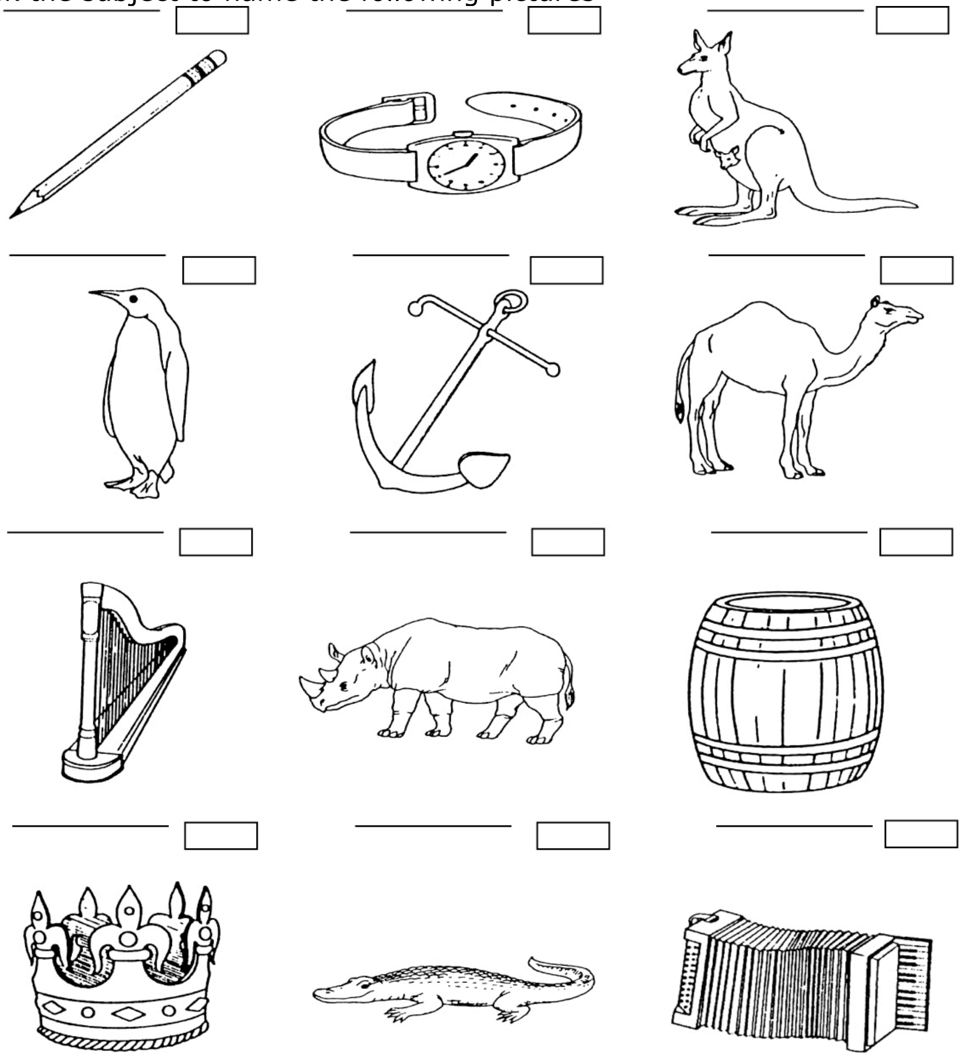<br>: | [Score 0-2]<br>pencil +<br>watch<br>Language<br><br>[Score 0-10]<br>Language |

This protocol is confidential and the property of the University of Nottingham. No part of it may be transmitted, reproduced, published, or used by others persons without prior written authorisation from the University of Nottingham

| | |
|---|---|
| L A N G U A G E - Comprehension | |
| Using the pictures above, ask the subject to:<br>Point to the one which is associated with the monarchy<br>Point to the one which is a marsupial<br>Point to the one which is found in the Antarctic<br>Point to the one which has a nautical connection | [Score0-4]<br>Language |
| LANGUAGE- Reading | |
| Ask the subject to read the following words:<br>**Sew**<br>**Pint**<br>**Soot**<br>**Dough**<br>**height** | [Score 0-1]<br>Language |
| VISUOSPATIAL ABILITIES | |
| Overlapping pentagons: Ask the subject to copy this diagram: | [Score0-1]<br>Visuospatial |

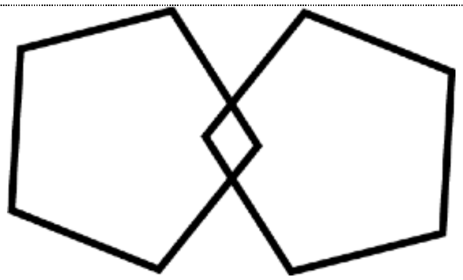

| | |
|---|---|
| Wire cube: Ask the subject to copy this drawing (for scoring, see instructions guide) | [Score 0-2]<br>Visuospatial |

This protocol is confidential and the property of the University of Nottingham. No part of it may be transmitted, reproduced, published, or used by others persons without prior written authorisation from the University of Nottingham

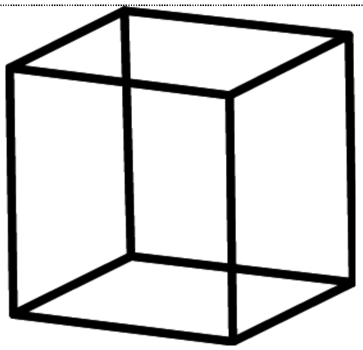

| | |
|---|---|
| Clock: Ask the subject to draw a clock face with numbers and the hands at ten past five. | [Score 0-5]<br>Visuospatial |

| PERCEPTUAL ABILITIES | |
|---|---|
| Ask the subject to count the dots without pointing them | Score 0-4]<br>Visuospatial |

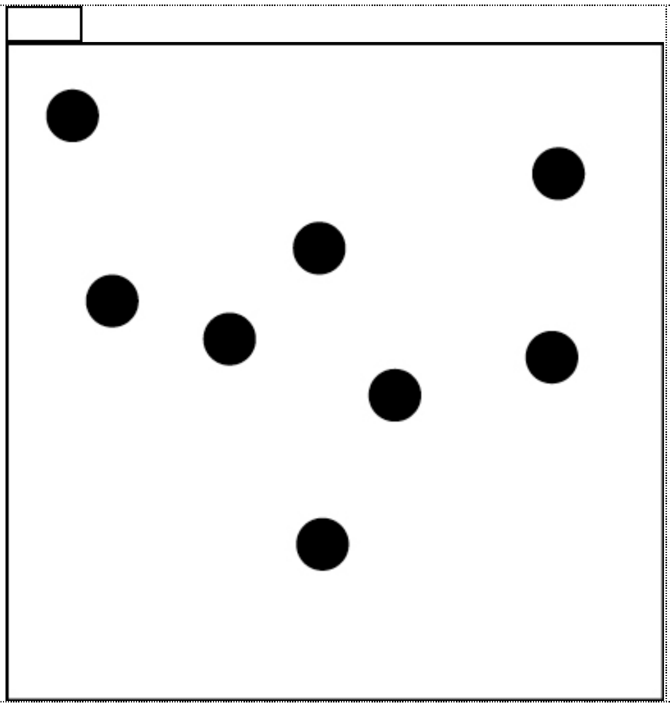

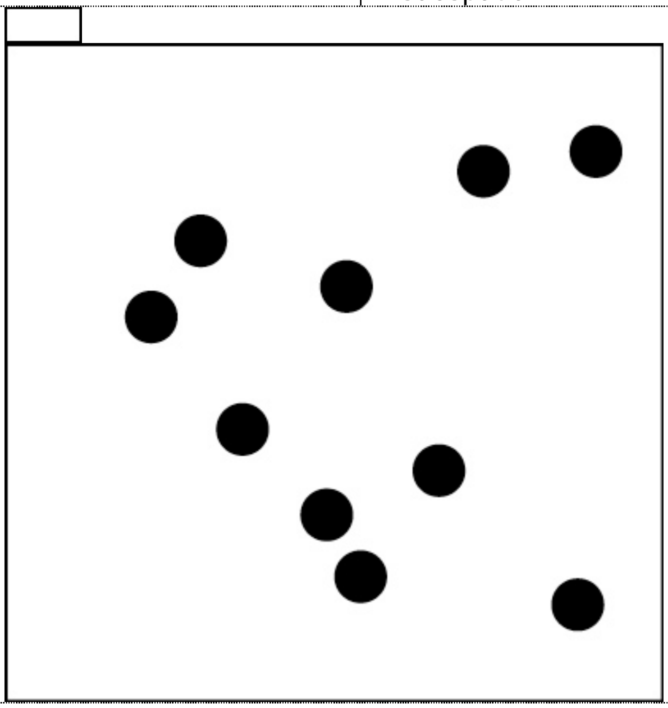

This protocol is confidential and the property of the University of Nottingham. No part of it may be transmitted, reproduced, published, or used by others persons without prior written authorisation from the University of Nottingham

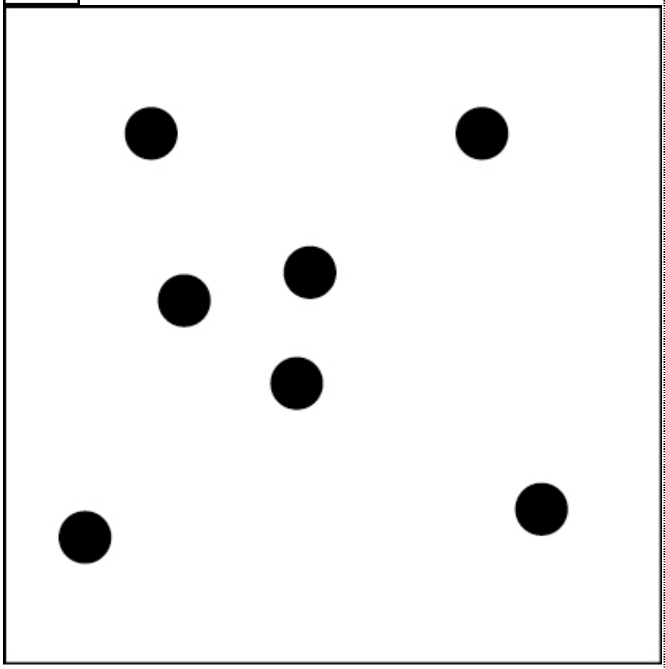

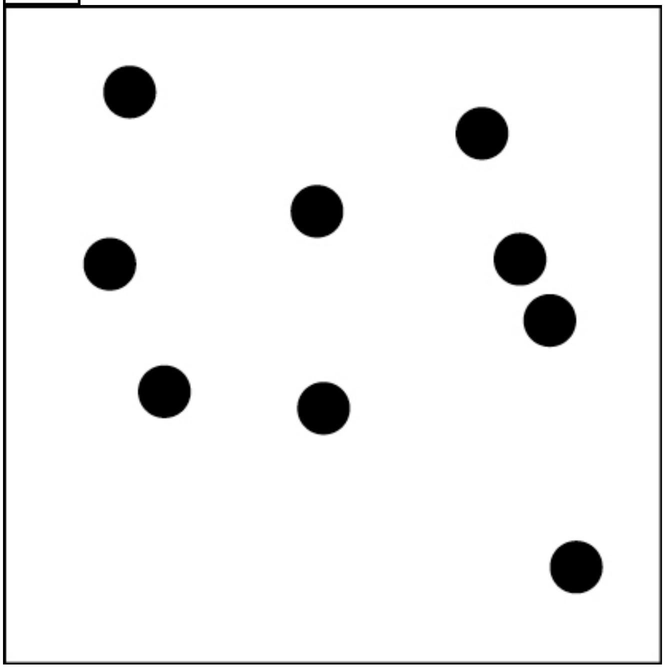

PERCEPTUAL ABILITIES

| | |
|---|---|
| Ask the subject to identify the letters | [Score 0-4] Visuospatial |

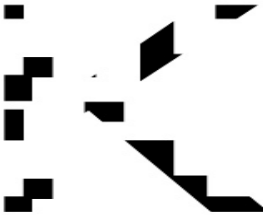

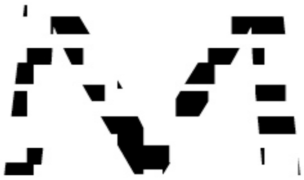

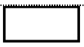

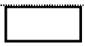

This protocol is confidential and the property of the University of Nottingham. No part of it may be transmitted, reproduced, published, or used by others persons without prior written authorisation from the University of Nottingham

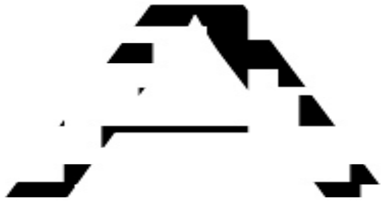

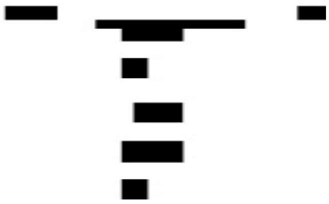

| RECALL | | |
|---|---|---|
| Ask "Now tell me what you remember of that name and address we were repeating at the beginning". | | |
| Harry Barnes<br>73 Orchard Close<br>Kingsbridge<br>Devon | _ _<br>_ _ _<br>_<br>- | [Score 0-7]<br>☐ Memory |

| RECOGNITION | | | | | | | |
|---|---|---|---|---|---|---|---|
| This test should be done if subject failed to recall one or more items. If all items were recalled,skip the test and score 5. If only part is recalled start by ticking items recalled in the shadowed column on the right hand side. Then test not recalled items by telling 'OK, I'll give you some hints: was the name X,Y or Z?" and so on. Each recognised item scores one point which is added to the point gained by recalling. | | | | | | [Score 0-5]<br>☐<br>Memory | |
| Jerry Barnes | | Harry Barnes | | Harry Bradford | | recalled | |
| 37 | | 73 | | 76 | | recalled | |
| Orchard Place | | Oak Close | | Orchard Close | | recalled | |
| Oakhampton | | Kingsbridge | | Dartington | | recalled | |
| Devon | | Dorset | | Somerset | | recalled | |

| General Scores | |
|---|---|
| MMSE | /30 |
| ACE-R | /100 |

| Subscores | |
|---|---|
| Attention and Orientation | /18 |
| Memory | /26 |
| Fluency | /14 |
| Language | /26 |
| Visuospatial | /16 |

This protocol is confidential and the property of the University of Nottingham. No part of it may be transmitted, reproduced, published, or used by others persons without prior written authorisation from the University of Nottingham

## Appendix B. Mini Mental state Examination (MMSE)

The MMSE will be modified to include death (thereby mimicking modification of functional outcome, e.g. Rankin Scale, to include death); participants who die will be assigned a MMSE score of -1.

| No | Question and Instructions | Maximum Score | Patient's Score |
|---|---|---|---|
| 1 | "What is the year? Season? Date? Day of the week? Month?" | 5 | |
| 2 | "Where are we now: State? County? Town/city? Hospital? Floor?" | 5 | |
| 3 | The examiner names three unrelated objects clearly and slowly, then asks the patient to name all three of them. The patient's response is used for scoring. The examiner repeats them until patient learns all of them, if possible. Number of trials: ___________ | 3 | |
| 4 | "I would like you to count backward from 100 by sevens." (93, 86, 79, 72, 65, …) Stop after five answers.<br>Alternative: "Spell WORLD backwards." (D-L-R-O-W) | 5 | |
| 5 | "Earlier I told you the names of three things. Can you tell me what were they?" | 3 | |
| 6 | Show the patient two simple objects, such as a wristwatch and a pencil, and ask the patient to name them. | 2 | |
| 7 | "Repeat the phrase: 'No ifs, ands, or buts.'" | 1 | |
| 8 | "Take the paper in your right hand, fold it in half, and put it on the floor." (The examiner gives the patient a piece of blank paper.) | 3 | |
| 9 | "Please read this and do what it says." (Written instruction is "Close your eyes.") | 1 | |
| 10 | "Make up and write a sentence about anything." (This sentence must contain a noun and a verb.) | 1 | |
| 11 | "Please copy this picture." (The examiner draws a picture of intersecting pentagons and gives the patient a blank piece of paper and asks him/her to copy the picture. All 10 angles must be present and the two pentagons must intersect.) | 1 | |
| 12 | Total Score | 30 | |

See (3)

This protocol is confidential and the property of the University of Nottingham. No part of it may be transmitted, reproduced, published, or used by others persons without prior written authorisation from the University of Nottingham

**Appendix C. telephone version of MMSE (t-MMSE)**

| QUESTIONS | Maximum score | Patient's score |
|---|---|---|
| What is the year/ season/date/day/month? | 5 | |
| Where are we now- building/city/county/country? | 4 | |
| I am going to name three objects and I want you to repeat it after me. They are apple, table and coin. Please repeat them | 3 | |
| Can you subtract 7 from 100 (93,86,79,72,65) | 5 | |
| Can you recall the three words I asked you to remember | 3 | |
| Can you repeat "No ifs, ands or buts" | 1 | |
| Tell me what is the thing called that you are speaking into as you talk to me | 1 | |
| Total score | 22 | |

This protocol is confidential and the property of the University of Nottingham. No part of it may be transmitted, reproduced, published, or used by others persons without prior written authorisation from the University of Nottingham

## Appendix D. Telephone Instrument for Cognition Scale-M

Please note that this test is designed for telephone use. In the event follow up is done in person the entire test must be completed verbally, i.e. the memory words must not be shown to the participant. Score 1 point for each correct answer.

| Question and Instructions | | Score |
|---|---|---|
| **Orientation:** Please ask them what day, date etc it is | | **7** |
| Day | | ☐ |
| Date | | ☐ |
| Month | | ☐ |
| Season | | ☐ |
| Year | | ☐ |
| Age | | ☐ |
| Telephone Number (code+number) | | |
| **Registration** | | **10** |
| I am going to read you a list of 10 words. Please listen carefully and try to remember them. When I am done, tell me as many as you can in any order. Ready? | | |
| Cabin | | ☐ |
| Pipe | | ☐ |
| Elephant | | ☐ |
| Chest | | ☐ |
| Silk | | ☐ |
| Theatre | | ☐ |
| Watch | | ☐ |
| Whip | | ☐ |
| Pillow | | ☐ |
| Giant | | ☐ |
| **Attention and Calculation** | | **6** |
| Please take away 7 from 100. Now continue to take 7 away from what you have left over until I ask you to stop | | |
| 93 | | ☐ |
| 86 | | ☐ |
| 79 | | ☐ |
| 72 | | ☐ |
| 65 | | ☐ |
| Count backwards Please count back 20-1 | | |
| No mistakes | | ☐ |
| **Comprehension, Semantic and Recent Memory** | | **5** |
| What do people use to cut paper? | Scissors | ☐ |
| What is the prickly green plant found in the desert? | Cactus | ☐ |
| Who is the Prime Minister? | Correct surname | ☐ |
| Who is the reigning monarch? | E,QE,QE2 | ☐ |
| What is the opposite direction to east? t | West | ☐ |
| **Language/Repetition** | | **1** |
| Please listen carefully and repeat No ifs ands or buts' | | ☐ |

This protocol is confidential and the property of the University of Nottingham. No part of it may be transmitted, reproduced, published, or used by others persons without prior written authorisation from the University of Nottingham

Score only if exactly right

**Delayed Recall** **10**

Please repeat as many of the 10 words I asked you to remember earlier

Cabin ☐
Pipe ☐
Elephant ☐
Chest ☐
Silk ☐
Theatre ☐
Watch ☐
Whip ☐
Pillow ☐
Giant ☐

**Total Score** (1 point for each correct answer) **/39**

See (53)

This protocol is confidential and the property of the University of Nottingham. No part of it may be transmitted, reproduced, published, or used by others persons without prior written authorisation from the University of Nottingham

## Appendix E. Trail Making Test (TMT) Parts A &B

Instructions

Both parts of the Trail Making Test consist of 25 circles distributed over a sheet of paper. In Part A, the circles are numbered 1 – 25, and the participant should draw lines to connect the numbers in ascending order. In Part B, the circles include both numbers (1 – 13) and letters (A – L); as in Part A, the participant draws lines to connect the circles in an ascending pattern, but with the added task of alternating between the numbers and letters (i.e., 1-A-2-B-3-C, etc.). The participant should be instructed to connect the circles as quickly as possible, without lifting the pen or pencil from the paper. Time the participant as he or she connects the "trail." If the participant makes an error, point it out immediately and allow the participant to correct it. Errors affect the participant's score only in that the correction of errors is included in the completion time for the task. It is unnecessary to continue the test if the participant has not completed both parts after five minutes has elapsed.

Step 1: Give the participant a copy of the Trail Making Test Part A worksheet and a pen or pencil.

Step 2: Time the participant as he or she follows the “trail” made by the numbers on the test.

Step 3: Record the time.

Step 4: Repeat the procedure for Trail Making Test Part B.

*Scoring:*

Results for both TMT A and B are reported as the number of seconds required to complete the task; therefore, higher scores reveal greater impairment.

Average Deficient Rule of Thumb

Trail A 29 seconds > 78 seconds Most in 90 seconds

Trail B 75 seconds > 273 seconds Most in 3 minutes

This protocol is confidential and the property of the University of Nottingham. No part of it may be transmitted, reproduced, published, or used by others persons without prior written authorisation from the University of Nottingham

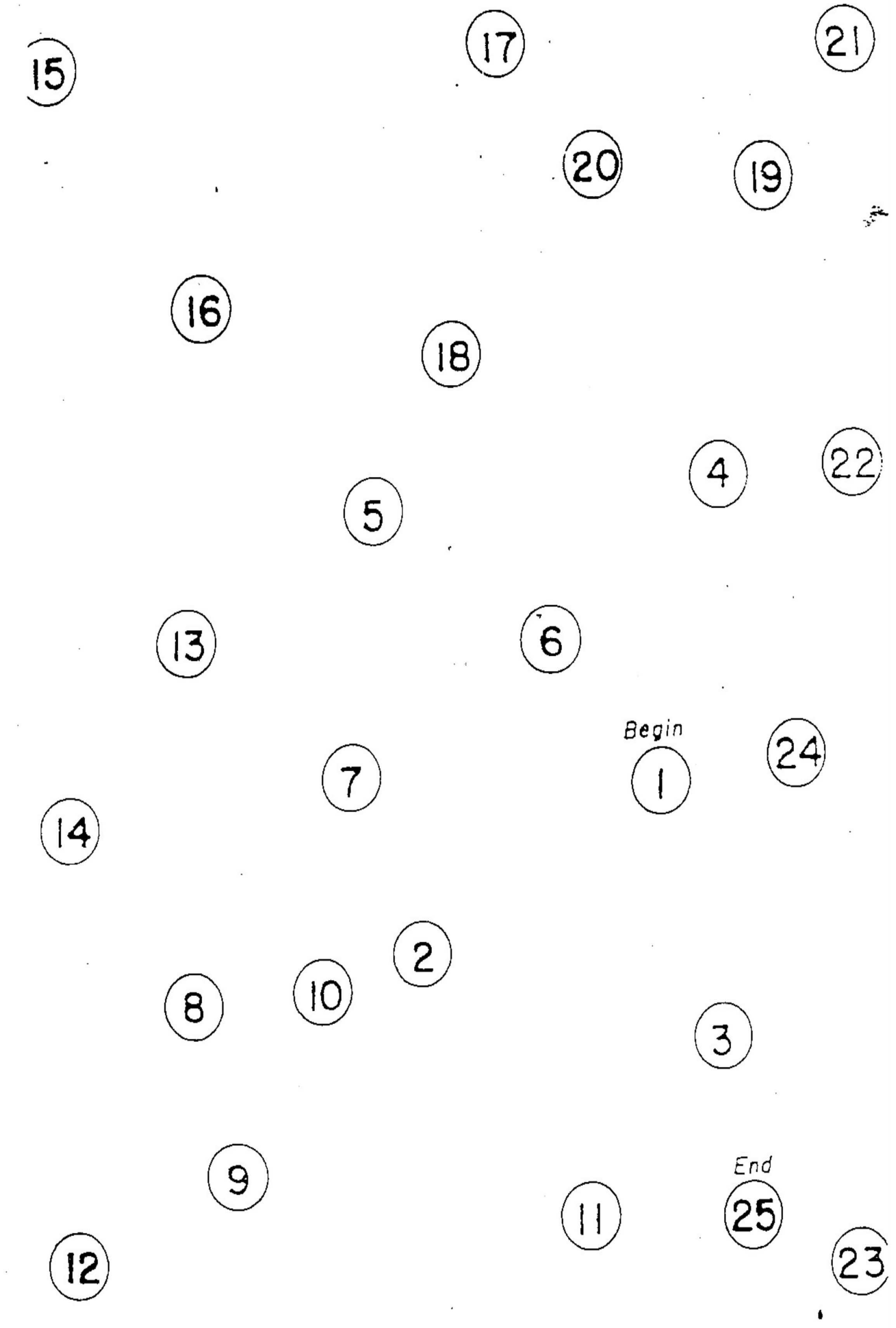


This protocol is confidential and the property of the University of Nottingham. No part of it may be transmitted, reproduced, published, or used by others persons without prior written authorisation from the University of Nottingham

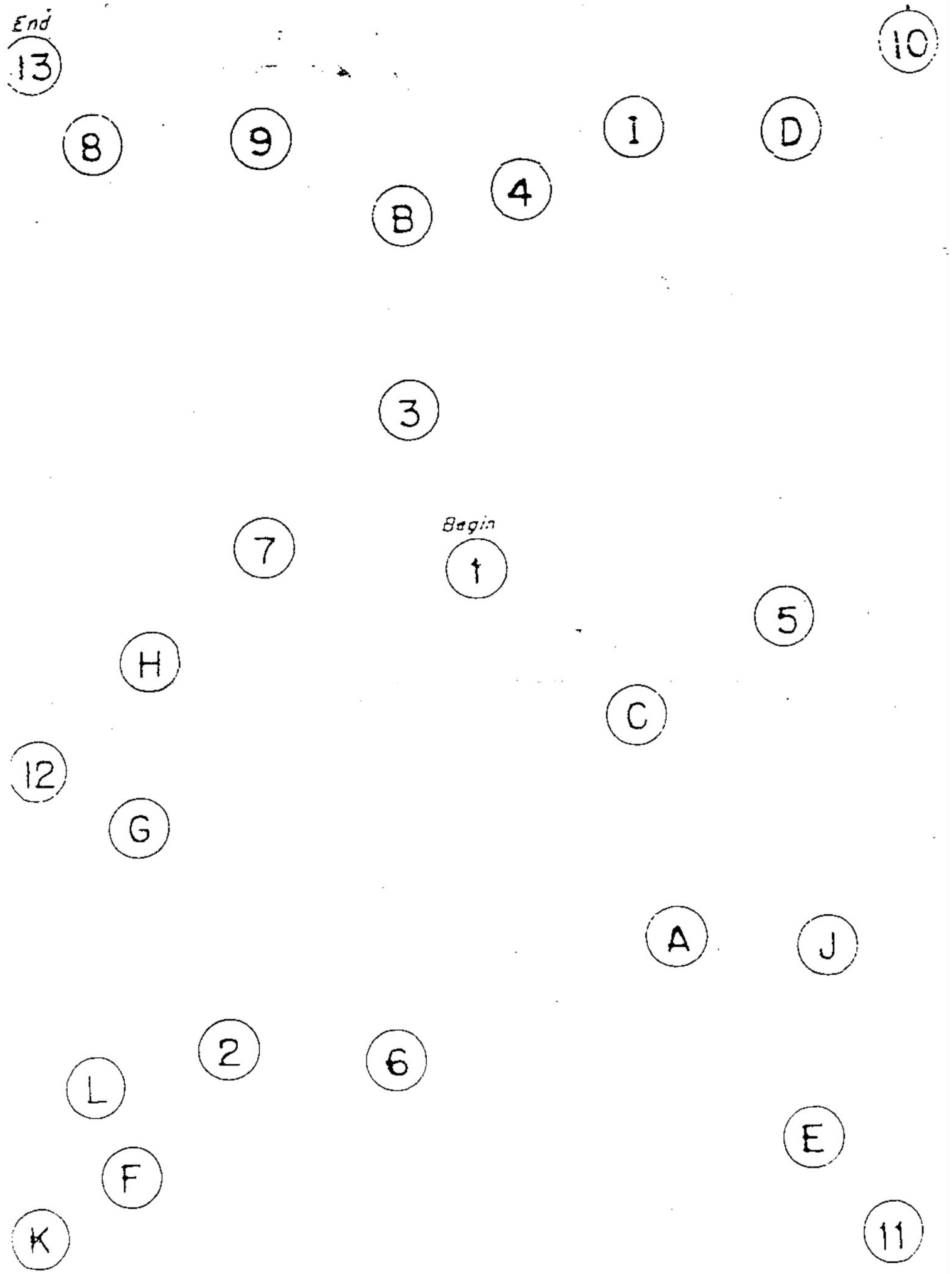


This protocol is confidential and the property of the University of Nottingham. No part of it may be transmitted, reproduced, published, or used by others persons without prior written authorisation from the University of Nottingham

## Appendix F. Modified Rankin Scale (mRS)

| | |
|---|---|
| 0 | No symptoms at all |
| 1 | No significant disability, despite symptoms; able to carry out all usual duties and activities |
| 2 | Slight disability; unable to carry out all previous activities but able to look after own affairs without assistance |
| 3 | Moderate disability; requiring some help, but able to walk without assistance |
| 4 | Moderately severe disability; unable to walk without assistance and unable to attend to own bodily needs without assistance |
| 5 | Severe disability; bedridden, incontinent and requiring constant nursing care and attention |
| 6 | Dead |

Score out of 6 (range 0-6)

See (13, 14)

This protocol is confidential and the property of the University of Nottingham. No part of it may be transmitted, reproduced, published, or used by others persons without prior written authorisation from the University of Nottingham

## Appendix G. Barthel Index (BI)

| Task | Criteria | Score |
|---|---|---|
| Bowels | Incontinent | 0 |
| | Occasional accident (once per week) | 5 |
| | Continent | 10 |
| Bladder | Incontinent, or catheterised and unable to manage alone | 0 |
| | Occasional accident (maximum once per 24 hours) | 5 |
| | Continent | 10 |
| Grooming | Needs help with personal care | 0 |
| | Independent face/hair/teeth/shaving (implements provided) | 5 |
| Toilet use | Dependent | 0 |
| | Needs some help, but can do something alone | 5 |
| | Independent (on and off, dressing, wiping) | 10 |
| Feeding | Unable | 0 |
| | Needs help cutting, spreading butter, etc. | 5 |
| | Independent | 10 |
| Transfer (bed to chair and back) | Unable, no sitting balance | 0 |
| | Major help (one or two people, physical), cab sit | 5 |
| | Minor help (verbal or physical) | 10 |
| | Independent | 15 |
| Mobility | Immobile | 0 |
| | Wheelchair independent, including corners | 5 |
| | Walks with help of one person (verbal or physical) | 10 |
| | Independent (but may use any aid: for example stick) | 15 |
| Dressing | Dependent | 0 |
| | Needs help but can do about half unaided | 5 |
| | Independent (including buttons, zips, laces, etc.) | 10 |
| Stairs | Unable | 0 |
| | Needs help (verbal, physical, carrying aid) | 5 |
| | Independent | 10 |
| Bathing | Dependent | 0 |
| | Independent (or in shower) | 5 |
| Total Score | | /100 |

See (14, 15)

This protocol is confidential and the property of the University of Nottingham. No part of it may be transmitted, reproduced, published, or used by others persons without prior written authorisation from the University of Nottingham

## Appendix H. EuroQoL

By placing a tick in one box in each group below, please indicate which statements best describes your own health state today.

| **Mobility** | **Tick appropriate box** |
|---|---|
| I have no problems in walking about | ☐ |
| I have some problems in walking about | ☐ |
| I am confined to bed | ☐ |
| **Self-Care** | |
| I have no problems with self care | ☐ |
| I have some problems with washing or dressing | ☐ |
| I am unable to wash or dress myself | ☐ |
| **Usual Activities (e.g. work, study, housework, family or leisure activities)** | |
| I have no problems performing my usual activities | ☐ |
| I have some problems performing usual activities | ☐ |
| I am unable to perform my usual activities | ☐ |
| **Pain/Discomfort** | |
| I have no pain or discomfort | ☐ |
| I have moderate pain or discomfort | ☐ |
| I have extreme pain or discomfort | ☐ |
| **Anxiety/Depression** | |
| I am not anxious or depressed | ☐ |
| I am moderately anxious or depressed | ☐ |
| I am extremely anxious or depressed | ☐ |

This protocol is confidential and the property of the University of Nottingham. No part of it may be transmitted, reproduced, published, or used by others persons without prior written authorisation from the University of Nottingham

## EUROQOL-VAS

We would like you to indicate on this scale how good or bad your own health is today, in your opinion. Please do this by drawing a line from the box below to whichever point on the scale indicates how good or bad your health state today is.

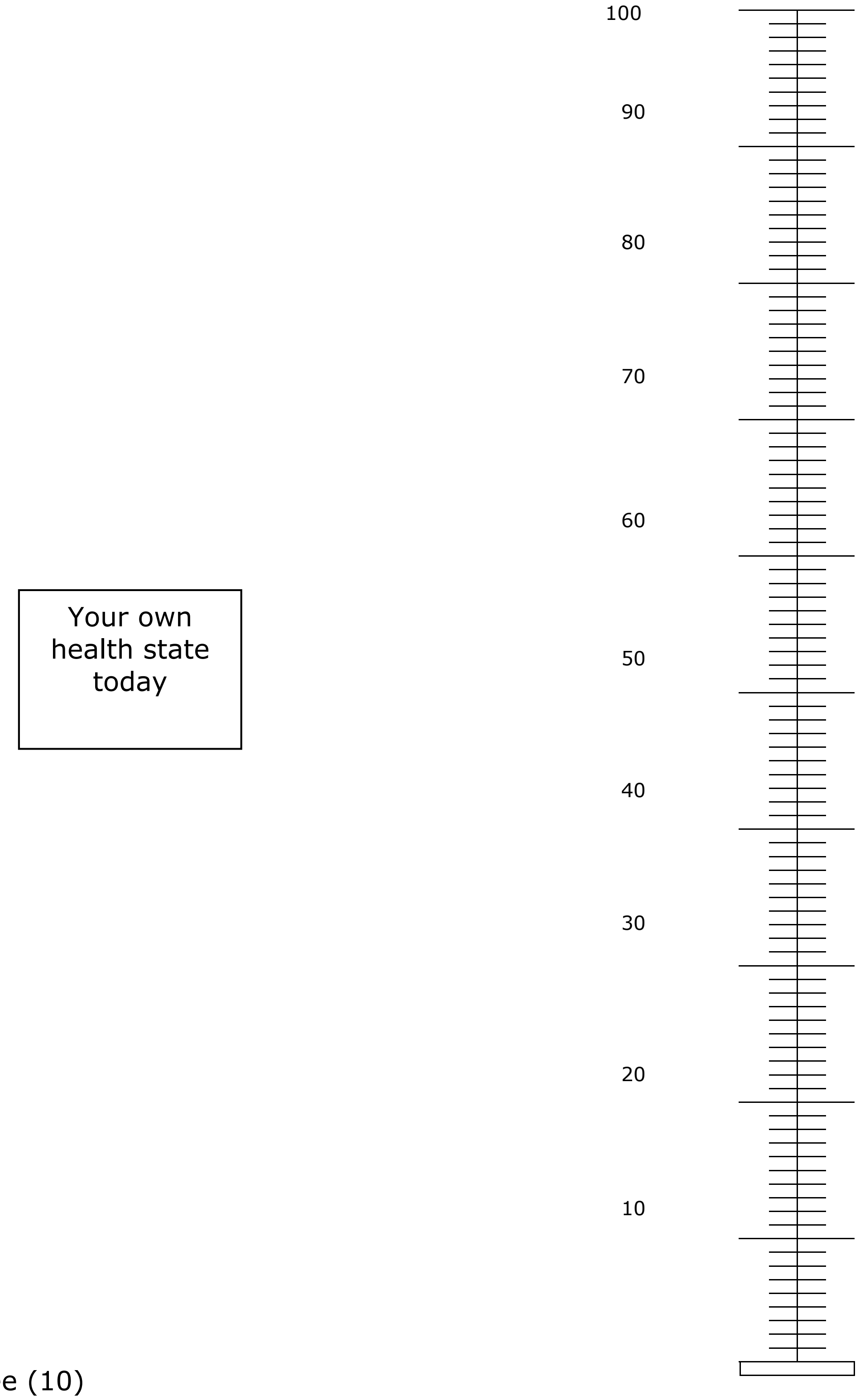


See (10)

This protocol is confidential and the property of the University of Nottingham. No part of it may be transmitted, reproduced, published, or used by others persons without prior written authorisation from the University of Nottingham

## Appendix I: Informant Questionnaire on Cognitive Decline in the Elderly (IQCODE)

We want you to remember what your friend or relative was like during the last follow-up and to compare it with what he/she is like now. The last follow-up was in 20__. Below are situations where this person has to use his/her memory or intelligence and we want you to indicate whether this has improved, stayed the same, or got worse in that situation over the past 1 year. Note the importance of comparing his/her present performance *with the last follow-up.* So if during the last follow-up this person always forgot where he/she had left things, and he/she still does, then this would be considered 'Hasn't changed much'. Please indicate the changes you have observed by *circling the appropriate answer.*

*Compared with the last follow-up* how is this person at:

| | 1 | 2 | 3 | 4 | 5 |
|---|---|---|---|---|---|
| 1. Recognizing the faces of family and friends | Much improved | A bit improved | Not much change | A bit worse | Much worse |
| 2. Remembering the names of family and friends | Much improved | A bit improved | Not much change | A bit worse | Much worse |
| 3. Remembering things about family and friends e.g. occupations, birthdays, addresses | Much improved | A bit improved | Not much change | A bit worse | Much worse |
| 4. Remembering things that have happened recently | Much improved | A bit improved | Not much change | A bit worse | Much worse |
| 5. Recalling conversations a few days later | Much improved | A bit improved | Not much change | A bit worse | Much worse |
| 6. Forgetting what he/she wanted to say in the middle of a conversation | Much improved | A bit improved | Not much change | A bit worse | Much worse |
| 7. Remembering his/her address and telephone number | Much improved | A bit improved | Not much change | A bit worse | Much worse |
| 8. Remembering what day and month it is | Much improved | A bit improved | Not much change | A bit worse | Much worse |
| 9. Remembering where | Much | A bit | Not | A bit | Much worse |

This protocol is confidential and the property of the University of Nottingham. No part of it may be transmitted, reproduced, published, or used by others persons without prior written authorisation from the University of Nottingham

| | | | | | |
|---|---|---|---|---|---|
| things are usually kept | improved | improved | much change | worse | |
| 10. Remembering where to find things which have been put in a different place from usual | Much improved | A bit improved | Not much change | A bit worse | Much worse |
| 11. Adjusting to any change in his/her day-to-day routine | Much improved | A bit improved | Not much change | A bit worse | Much worse |
| 12. Knowing how to work familiar machines around the house | Much improved | A bit improved | Not much change | A bit worse | Much worse |
| 13. Learning to use a new gadget or machine around the house | Much improved | A bit improved | Not much change | A bit worse | Much worse |
| 14. Learning new things in general | Much improved | A bit improved | Not much change | A bit worse | Much worse |
| 15. Remembering things that happened to him/her when he/she was young | Much improved | A bit improved | Not much change | A bit worse | Much worse |
| 16. Remembering things he/she learned when he/she was young | Much improved | A bit improved | Not much change | A bit worse | Much worse |
| 17.Understanding the meaning of unusual words | Much improved | A bit improved | Not much change | A bit worse | Much worse |
| 18.Understanding magazine or newspaper articles | Much improved | A bit improved | Not much change | A bit worse | Much worse |
| 19.Following a story in a book or on TV | Much improved | A bit improved | Not much change | A bit worse | Much worse |
| 20. Composing a letter to friends or for business purposes | Much improved | A bit improved | Not much change | A bit worse | Much worse |
| 21. Knowing about important historical events of the past | Much improved | A bit improved | Not much change | A bit worse | Much worse |
| 22. Making decisions on | Much | A bit | Not | A bit | Much worse |

This protocol is confidential and the property of the University of Nottingham. No part of it may be transmitted, reproduced, published, or used by others persons without prior written authorisation from the University of Nottingham

| | | | | | |
|---|---|---|---|---|---|
| everyday matters | improved | improved | much change | worse | |
| 23. Handling money for shopping | Much improved | A bit improved | Not much change | A bit worse | Much worse |
| 24. Handling financial matters, e.g. the pension, dealing with the bank | Much improved | A bit improved | Not much change | A bit worse | Much worse |
| 25. Handling other everyday arithmetic problems, e.g. knowing how much food to buy, knowing how long between visits from family or friends | Much improved | A bit improved | Not much change | A bit worse | Much worse |
| 26. Using his/her intelligence to understand what's going on and to reason things through | Much improved | A bit improved | Not much change | A bit worse | Much worse |

This protocol is confidential and the property of the University of Nottingham. No part of it may be transmitted, reproduced, published, or used by others persons without prior written authorisation from the University of Nottingham

**Appendix J. Zung Depression rating Scale (short)**

The next set of questions is asking about your mood and how you feel in yourself. Answer these questions by placing a tick in each group below. Please indicate which mood describes you best today.

| | Seldom or never | Some of the time | Good part of the time | Most of the time |
|---|---|---|---|---|
| I feel down-hearted and blue | ☐ | ☐ | ☐ | ☐ |
| Morning is when I feel best | ☐ | ☐ | ☐ | ☐ |
| I have trouble sleeping at night | ☐ | ☐ | ☐ | ☐ |
| I can eat as much as I used to | ☐ | ☐ | ☐ | ☐ |
| I get tired for no reason | ☐ | ☐ | ☐ | ☐ |
| I find it difficult to make decisions | ☐ | ☐ | ☐ | ☐ |
| I feel hopeful about the future | ☐ | ☐ | ☐ | ☐ |
| I feel that I am useful and needed | ☐ | ☐ | ☐ | ☐ |
| My life is some what empty | ☐ | ☐ | ☐ | ☐ |
| I still enjoy the things I used to do | ☐ | ☐ | ☐ | ☐ |

Short Zung IDS Index = 100 x Total / 40

Depression $\geq$ 70

See (12, 14, 54)

This protocol is confidential and the property of the University of Nottingham. No part of it may be transmitted, reproduced, published, or used by others persons without prior written authorisation from the University of Nottingham

## Appendix K Definitions

### Acute Stroke Unit

A high-dependency nursing unit (or area) caring only/mainly for participants with acute stroke and providing close monitoring of neurological and vascular signs.

### Bleeding

#### *Major bleed*

These will constitute a serious adverse event.

Fatal bleeding, and/or

Symptomatic bleeding in a critical area or organ, such as intracranial, intraspinal, intraocular, retroperitoneal, intraarticular or pericardial, or intramuscular with compartment syndrome, and/or

Bleeding causing fall in haemoglobin of 2 g/l (1.24 mmol/l) or more, or leading to transfusion of 2 or more units of whole blood or red cells.

#### *Moderate bleed*

Not major, and

Bleeding causing fall in haemoglobin of 1-2 g/l, and leading to no transfusion, or transfusion of only 1 unit of whole blood or red cells.

#### *Minor bleed*

Not major or moderate, and

Comprising bruising, ecchymoses, gingival bleed or similar other type bleeding.

### Bleeding on CT/MRI head scans:

#### *Haemorrhagic Infarct (HI)*

Petechial infarction without space occupying effect.

HI1 - small petechiae

HI2 - more confluent petechiae

#### *Parenchymal Haemorrhage (PH)*

Haemorrhage with mass effect.

PH1 - <30% of the infarcted area with mild space occupying effect

This protocol is confidential and the property of the University of Nottingham. No part of it may be transmitted, reproduced, published, or used by others persons without prior written authorisation from the University of Nottingham

PH2 - >30% of the infarcted area with significant space occupying effect

**Cognitive decline**

A reduction in the ACE-R of <10 points or to <70(2).

**Cognitive impairment**

ACE-R score 70 points or lower.

**Dementia**

As defined by DSM IV

1. Impairment of two or more of the following areas of cognition, sufficient to interfere with work, social function, or relationships:
   Memory
   Language
   Abstract thinking and judgement
   Praxis
   Visuospatial or perceptual skills
   Personality
   Social conduct
2. The absence of the features of delirium
3. The exclusion of non-organic psychiatric disorders, for example major depression or schizophrenia.

See (55)

**Disposition**

Home, institution (e.g. warden controlled; nursing home), dead

**Muscle Problems related to statins**

We will define muscle problems related to statins as per the ACC/AHA/NHLBI advisory on the use and safety of statins(56).

***Myalgia*** : muscle ache or weakness without creatine kinase (CK) elevation.

***Myositis*** : muscle symptoms with increased CK levels.

***Rhabdomyolysis*** : muscle symptoms with marked CK elevation (typically >10 times upper limit of normal) and creatinine elevation (usually with brown urine and urine myoglobin).

**Neurological deterioration**

A reduction in NIHSS of $\geq$ 4 points, or decrease in consciousness level by $\geq$ 3 points, as compared with baseline.

This protocol is confidential and the property of the University of Nottingham. No part of it may be transmitted, reproduced, published, or used by others persons without prior written authorisation from the University of Nottingham

***Informant (consultee)***

A partner, sibling, child, or friend who is willing and able to attend clinics with the participant and who will provide structured information about the participant.

**Recurrent stroke**

Classified as haemorrhagic or ischaemic (if documented by CT scan or autopsy), or of unknown type. The time from stroke onset and side will be noted.

**Significant hypotension**

A symptomatic fall in blood pressure of >20% as compared with baseline necessitating intervention with cessation or weaning of BP drugs.

**Statin Classification ('guideline' statins and 'intensive' statins)**

***'Guideline' statins***: Simvastatin ≤ 40 mg, any dose of Pravastatin or Fluvastatin, Atorvastatin ≤20 mg,

***'Intensive' statins:*** Atorvastatin >20 mg, Rosuvastatin

**Stroke Rehabilitation Unit**

A dedicated rehabilitation unit (or area) caring only/mainly for participants with recent stroke and providing multi-disciplinary therapy (e.g. physiotherapy, occupational therapy, speech & language therapy).

**Stroke**

A clinical syndrome characterised by rapidly developing clinical symptoms and/or signs of focal (and at times global) loss of cerebral function with symptoms lasting for more than 24 hours or leading to death, with no apparent cause other than that of vascular origin'.(57)

**Transient Ischaemic Attack (TIA)**

A sudden focal neurological deficit of the brain or eye, presumed to be of vascular origin and lasts less than 24 hours.

**Symptomatic intracranial haemorrhage**

Neurological deterioration (see above), or death, and intracranial haemorrhage (of PH type) found on CT scan or autopsy. See (58)

This protocol is confidential and the property of the University of Nottingham. No part of it may be transmitted, reproduced, published, or used by others persons without prior written authorisation from the University of Nottingham

## REFERENCES

1. The ENOS Trial Investigators. Glyceryl trinitrate vs. control, and continuing vs. stopping temporarily prior antihypertensive therapy, in acute stroke: rationale and design of the Efficacy of Nitric Oxide in Stroke (ENOS) trial (ISRCTN99414122). International Journal of Stroke. 2006 November;1(4):245-9.
2. Mathuranath PS, Nestor PJ, Berrios GE, Rakowicz W, Hodges JR. A brief cognitive test battery to differentiate Alzheimer's disease and frontotemporal dementia. Neurology. 2000 Dec 12;55(11):1613-20.
3. Folstein M, Folstein S, McHugh P, 1646. "Mini-mental state"- a practical method for grading the cognitive state of patients for the clinician. Journal Psychiatric Research. 1975;12:189-98.
4. McKhann G, Drachman D, Folstein M, Katzman R, Price D, Stadlan EM. Clinical disagnosis of Alzheimer's disease: report of the NINCDS-ADRDA Work Group under the auspeices of Department of Health and Human Services Task Force on Alzheimer's Disease. Neurology. 1984;34(7):939-44.
5. Roman GC, T.K. T, T. E, Cummings JL, Masdeu JC, Garcia JH, et al. Vascular dementia: diagnostic criteria for research studies. Report of the NINDS-AIREN International Workshop. Neurology. 1993;43(2):250-60.
6. Desmond DW, Tatemichi TK, Hanzawa L. The telephone interview for cognitive status (TICS): Reliability and validity in a stroke sample. International journal of geriatric psychiatry. 1994;9:803-7.
7. Partington j, Leiter R. Partington's Pathway Test. The Psychological Service Center Bulletin 1. 1949:9-20.
8. Lezak M, DB H, DW L. Neurosyhological Assesssment. 4 ed. New York: Oxford University Press; 2004.
9. Jorm AF, Jacomb PA. The informant questionnaire on cognitive decline in the elderly (IQCODE): socio-demographic correlates, reliability, validity and some norms. Psychological medicine. 1989;19:1015-22.
10. Dorman PJ, Slattery J, Farrell B, Dennis MS, Sandercock PAG, United Kingdom Collaborators in the International Stroke Trial. A randomised comparison of the EuroQol and short form-36 after stroke. BrMedJ. 1997;315:461.
11. Zung WWK. A self-rating depression scale. Archives of General Psychiatry. 1965;12:63-70.
12. Tucker MA, Ogle SJ, Davison JG, Eilenberg MD, 1740. Validation of a brief screening test for depression in the elderly. Age and Ageing. 1987;16:139-44.
13. Rankin J. Cerebral vascular accidents in patients over the age of 60. 2. Prognosis. Scottish Medical Journal. 1957;2:200-15.
14. Wade DT. Measurement in neurological rehabilitation. Oxford: Oxford University Press; 1992.
15. Mahoney FI, Barthel DW. Functional evaluation: The Barthel Index. Maryland State Medical Journal. 1965:61-5.
16. Bath PM, Geeganage C, Gray LJ, Collier T, Pocock S. Use of ordinal outcomes in vascular prevention trials. Comparison with binary outcomes in published trials. Stroke. 2008;39(10):2817-23.



This protocol is confidential and the property of the University of Nottingham. No part of it may be transmitted, reproduced, published, or used by others persons without prior written authorisation from the University of Nottingham

17. Weir CJ, Lees KR. Comparison of stratification and adaptive methods for treatment allocation in an acute stroke clinical trial. StatMed. 2003;22(5):705-26.
18. Lenzi GL, Altieri M. Short-term evolution as a marker of vascular dementia versus Alzheimer's disease. Journal of the Neurological Sciences. 2007;257:182-4.
19. Butterworth RJ, Marshall WJ, Bath PMW. Changes in serum lipid measurements following acute ischaemic stroke. Cerebrovascular Diseases. 1997;7:10-3.
20. National Institute for Health and Clinical Excellence N. Hypertension: management of hypertension in adults in primary care. In: Health, editor.; 2006.
21. Chapman N, Dobson J, Wilson S, Dahlof B, Sever PS, Wedel H, et al. Effect of spironolactone on blood pressure in subjects with resistant hypertension. Hypertension. 2007;49:839-45.
22. PROGRESS Collaborative Group. Randomised trial of a perindopril-based blood-pressure-lowering regimen among 6105 individuals with previous stroke or transient ischaemic attack. Lancet. 2001;358:1033-41.
23. O'Brien E. unknown. Journal of Hypertension. 1994;12.
24. Dahlof B, Sever PS, Poulter NR, Wedel H, Beevers GD, Caulfield M, et al. Prevention of cardiovascular events with an antihypertensive regimen of amlodipine adding perindopril as required versus atenol adding bendroflumethiazide as required, in the Anglo-Scandinavian Cardiac Outcomes Trial-Blood Pressure Lowering Arm (ASCOT-BPLA): a multicentre randomised controlled trial. Lancet. 2005;366:895-906.
25. Rashid P, Weaver C, Leonardi-Bee JA, Fletcher S, Bath FJ, Bath PMW. The effects of transdermal glyceryl trinitrate, a nitric oxide donor on blood pressure, cerebral and cardiac haemodynamics and plasma nitric oxide levels in acute stroke. J Stroke Cerebrovasc Dis. 2003;13:143-51.
26. Gray LJ, Sprigg N, Rashid PA, Willmot M, Bath PMW. Effect of nitric oxide donors on blood pressure and pulse pressure in acute and sub-actue stroke. Journal of Stroke and Cerebrovascular Diseases. 2006;15(6):245-9.
27. Geeganage C, Sare GM, Bath PMW. Pulse pressure as a predictor of stroke. Expert Rev Neuroptherapeutics. 2008;8(2):165-67.
28. National Institute for Health and Clinical Excellence N. Cardiovascular risk assessment and the modification of blood lipids for the primary and secondary prevention of cardiovascular disease. In: Health, editor.; 2008.
29. National Institute for Health and Clinical Excellence N. Ezetimibe for the treatment of primary ( heterozygous-familial and non-familial) hypercholesterolaemia. 2007.
30. National Institute for Health and Clinical Excellence N. Cardiovascular disease-statins: guidance. In: Health, editor.; 2006.
31. Vergouwen MDI, de Haan RJ, Vermeulen M, Roos YBWEM. Statin treatment and occurrence of hemorrhagic stroke in patient with a history of cerebrovascular disease. Stroke. 2008;39:497-502.
32. Investigators. TSPbARiCLS. High-dose atorvastatin after stroke or transient ischemic attack. The New England Journal of Medicine. 2006;355(6):549-59.
33. Heart Protection Study Collaborative Group. MRC/BHF Heart Protection Study of cholesterol lowering with simvastatin in 20536 high-risk individuals: a randomised placebo-controlled trial. Lancet. 2002;360:7-22.
34. Hansson L, Zanchetti A. The hypertension optimal treatment (HOT) study: 24 month data on blood pressure and tolerability. Blood Pressure. 1997;6(5):313-7.



This protocol is confidential and the property of the University of Nottingham. No part of it may be transmitted, reproduced, published, or used by others persons without prior written authorisation from the University of Nottingham

35. Hansson L, Zanchetti A. The hypertension optimal treatment (HOT) study--patient characteristics: randomization, risk profiles, and early blood pressure results. Blood Pressure. 1994;3(5):322-7.
36. National Institute for Health and Clinical Excellence N. The diagnosis and acute management of stroke and transient ischaemic attacks. In: Health, editor.; 2008.
37. Tzourio C, Anderson C, Chapman N, Woodward M, Neal B, MacMahon S, et al. Effects of blood pressure lowering with perindopril and indapamide therapy on dementia and cognitive decline in patients with cerebrovascular disease. Arch Intern Med. 2003;163(9):1069-75.
38. The Optimising Analysis of Stroke Trials (OAST) Collaboration. Can we improve the statistical analysis of stroke trials? Statistical re-analysis of functional outcomes in stroke trials. Stroke 2007;38(6):1911-5.
39. The Optimising Analysis of Stroke Trials (OAST) Collaboration. Calculation of sample size for stroke trials assessing functional outcome: comparison of binary and ordinal approaches. International Journal of Stroke. 2008;3:78-84.
40. The Optimising Analysis of Stroke Trials (OAST) Collaboration. Should stroke trials adjust functional outcome for baseline prognostic factors? Stroke. 2009;40:888-94.
41. Sparks DL, Sabbagh MN, Connor DJ, Lopez J, Launer LJ, Brown P, et al. Atorvastatin for the treatment of mild to moderate Alzheimer disease. Arch Neurol. 2005;62:753-7.
42. Saxby BK, Harrington F, Wesnes KA, McKeith IG, Ford GA. Candesartan and cognitive decline in older patients with hypertension: a substudy of the SCOPE trial. Neurology. 2008;70(19 pt 2):1858-66.
43. Prince MJ, Bird AS, Blizard RA, Mann AH. Is the cognitive function of older patients affected by antihypertensive treatment? Reuslts from 54 months of the Medical Research Council's treatment trial of hypertension in older adults. British Medical Journal. 1996;312:801-5.
44. Simons M, Schwarzler F, Lutjohann D, von Bergmann K, Beyreuther K, Dichgans J, et al. Treatment with simvastatin in normocholesterolemic patients with Alzheimer's disease: A 26-week randomized, placebo-controlled, double-blind trial. Ann Neurol. 2002;52:346-50.
45. Applegate WB, Pressel S, Wittes J, Luhr J, Shekell RB, Camel GH, et al. Impact of the treatment of isolated systolic hypertension on behavioral variables. Arch Intern Med. 1994;154.
46. Skoog I, Lithell H, Hansson L, Elmfeldt D, Hofman A, Olofsson B, et al. Effect of baseline cognitive function and antihypertensive treatment on cognitive and cardiovascular outcomes: study on COgnition and Prognosis in the Elderly (SCOPE). American Journal of Hypertension. 2005;18:1052-59.
47. Shepherd J, Blauw GJ, Murphy MB, Bollen ELEM, Buckley BM, Cobbe SM, et al. Pravastatin in elderly individuals at risk of vascular disease (PROSPER): a randomised controlled trial. Lancet. 2002;360:1623-30.
48. Muldoon MF, Ryan CM, Sereika SM, Flory JD, Manuck SB. Randomized trial of the effects of simvastatin on cognitive functioning in hypercholesterolemic adults. The American Journal of Medicine. 2004;117(11):823-9.
49. Di Bari M, Pahor M, Franse LV, Shorr RI, Wan JY, Ferrucci L, et al. Dementia and disability outcomes in large hypertension trials: lessons learned from the systolic



This protocol is confidential and the property of the University of Nottingham. No part of it may be transmitted, reproduced, published, or used by others persons without prior written authorisation from the University of Nottingham

hypertension in the elderly program (SHEP) trial. American Journal of Epidemiology. 2001;153(1):72-8.
50. Committee for Proprietary Medicinal Products. Points to consider on clinical investigation of medicinal poducts for the treatment of acute stroke. London: The European Agency for the Evaluation of Medicinal Products; 2001 20 September 2001. Report No.: CPMP/EWP/560/98.
51. Whitehead J. Sample-Size Calculations for Ordered Categorical-Data Statistics in Medicine. 1993 Apr 30;12(24):2257-71.
52. Department of Health. HSG (96)48: NHS indemnity arrangements for handling clinical negligence claims against NHS staff. In: Health, editor.; 1996. p. 2.
53. de Jager CA, Budge MM, Clarke R. Utility of TICS-M for the assessment of cognitive function in older adults. International journal of geriatric psychiatry. 2003 Apr;18(4):318-24.
54. Korner BN, Wood DS, Siemiatycki J, Shapiro S, Becker R, 1479. Health-related information post discharge: telephone versus face-to-face interviewing. ArchPhysMedRehabil. 1994;75:1287-96.
55. American Psychiatric Association. Diagnostic and Statistical Manual of mental disorders IV. 2000.
56. Pasternak R, SMITH SJ, Bairey-Merz CN et al. ACC/AHA/NHLBI Clinical Advisory on the use and safety of statins. Journal of American College of Cardiology. 2002 August 7 2002;40(3):568-73.
57. Hatano S. Experience from a multicentre stroke register: a preliminary report. Bulletin of the World Health Organisation. 1976;54:541-53.
58. Bath PMW, on behalf of the TAIST Investigators and Advisory Committee. Tinzaparin in acute ischaemic stroke trial (TAIST). Copenhagen: EFNS; 2000.



This protocol is confidential and the property of the University of Nottingham. No part of it may be transmitted, reproduced, published, or used by others persons without prior written authorisation from the University of Nottingham
