# Supplementary material for: Baseline characteristics, analysis plan and report on feasibility for the Prevention Of Decline in Cognition After Stroke Trial (PODCAST)
Source: Trials. 2015 Nov 7;16:509. doi: 10.1186/s13063-015-1033-2 (PMC4636808; doi:10.1186/s13063-015-1033-2)
Supplement: Additional file 2: — Plan for reporting the main results. (DOCX 117 kb) [file 13063_2015_1033_MOESM2_ESM.docx]

**APPENDIX B. Planned data tables and figures in primary paper**

**Table 1.** Clinical characteristics at randomisation. Data are number (%), median [interquartile range] or mean (standard deviation).

|  | All | Intensive BP | Guideline BP | Intensive lipids | Guideline lipids |
| --- | --- | --- | --- | --- | --- |
| Number |  |  |  |  |  |
| Age (years) † |  |  |  |  |  |
| Sex, male (%) |  |  |  |  |  |
| Time to randomisation [months] † |  |  |  |  |  |
| Medical history (%) |  |  |  |  |  |
| Memory problem |  |  |  |  |  |
| Hypertension, treated |  |  |  |  |  |
| Hyperlipidaemia |  |  |  |  |  |
| Diabetes mellitus |  |  |  |  |  |
| Atrial fibrillation |  |  |  |  |  |
| Stroke |  |  |  |  |  |
| IHD |  |  |  |  |  |
| PAD |  |  |  |  |  |
| Smoking, ever (%) |  |  |  |  |  |
| Alcohol, >21 [upw] |  |  |  |  |  |
| Index stroke |  |  |  |  |  |
| Side, right weakness (%) |  |  |  |  |  |
| Dysphasia (%) † |  |  |  |  |  |
| BP drugs, number † |  |  |  |  |  |
| Mode |  |  |  |  |  |
| Median |  |  |  |  |  |
| Mean |  |  |  |  |  |
| >0 (%) |  |  |  |  |  |
| BP drug classes (%) |  |  |  |  |  |
| ACE-I or ARB |  |  |  |  |  |
| ß-receptor antagonist |  |  |  |  |  |
| Calcium channel blocker |  |  |  |  |  |
| Diuretic |  |  |  |  |  |
| Other |  |  |  |  |  |
| Lipid tablets (%) |  |  |  |  |  |
| Any statin † |  |  |  |  |  |
| Fluvastatin |  |  |  |  |  |
| Simvastatin |  |  |  |  |  |
| Atorvastatin |  |  |  |  |  |
| Rosuvastatin |  |  |  |  |  |
| Ezetimibe |  |  |  |  |  |
| Pre-morbid mRS 0/1 (%) † |  |  |  |  |  |
| ACE-R (/100) † |  |  |  |  |  |
| NIHSS, mean (/42) † |  |  |  |  |  |
| Median |  |  |  |  |  |
| OCSP Total anterior † |  |  |  |  |  |
| Systolic BP (mmHg) † |  |  |  |  |  |
| <140 (%) |  |  |  |  |  |
| <125 (%) |  |  |  |  |  |
| Diastolic BP (mmHg) |  |  |  |  |  |
| Heart rate (bpm) |  |  |  |  |  |
| Lipids (mmol/l) |  |  |  |  |  |
| Total cholesterol † |  |  |  |  |  |
| Triglycerides |  |  |  |  |  |
| HDL-cholesterol |  |  |  |  |  |
| LDL-cholesterol |  |  |  |  |  |
| Non-HDL-cholesterol |  |  |  |  |  |

† Minimisation variable – from June 2013 limited to age, ACE-R, systolic blood pressure and total cholesterol. ‡ Stratification variable; ∫ May exceed 100%; ¶ Protocol violation

Non-HDL-cholesterol = total cholesterol – HDL-cholesterol

ACE-R: Addenbrooke’s Cognitive Examination-revised; BP: blood pressure; bpm: beats per minute; HDL: high density lipoprotein; IHD: ischaemic heart disease; LDL: low density lipoprotein; mRS: modified Rankin Scale; NIHSS: National Institutes of Health Stroke Scale; OCSP: Oxfordshire Community Stroke Project; PAD: peripheral arterial disease; IHD: current angina or previous angina or myocardial infarction

**Table 2**. Adherence to BP and lipid lowering therapy through the trial, by intensive versus guideline blood pressure lowering, and by intensive versus guideline lipid lowering. Data are number of tablets, and number adjusted for dose as proportion of maximum dose.

| Month |  |  | 0 | 1 | 2 | 3 | 6 | 12 | 18 | 24 | 30 | 36 |
| --- | --- | --- | --- | --- | --- | --- | --- | --- | --- | --- | --- | --- |
| BP | Patients |  |  |  |  |  |  |  |  |  |  |  |
|  | Intensive | Tablets |  |  |  |  |  |  |  |  |  |  |
|  |  | Dose-adjusted |  |  |  |  |  |  |  |  |  |  |
|  | Guideline | Tablets |  |  |  |  |  |  |  |  |  |  |
|  |  | Dose-adjusted |  |  |  |  |  |  |  |  |  |  |
| Lipids | Patients |  |  |  |  |  |  |  |  |  |  |  |
|  | Intensive | Tablets |  |  |  |  |  |  |  |  |  |  |
|  |  | Dose-adjusted |  |  |  |  |  |  |  |  |  |  |
|  | Guideline | Tablets |  |  |  |  |  |  |  |  |  |  |
|  |  | Dose-adjusted |  |  |  |  |  |  |  |  |  |  |

Dose adjusted is sum of tablet dose / maximum licensed dose (e.g. amlodipine 5mg is 0.5)

**Table 3**. Blood pressure and lipids levels at baseline and months 1, 2, 3 and 6, by treatment group: intensive (BP: n=37, Lipid: n=35) vs guideline (BP: n=40, Lipid: n=32). Data are mean (standard deviation) at 0-6 months; comparison by ANCOVA with mean difference adjusted for baseline.

| Outcome | Grp | Baseline | Month 1 | Month 2 | Month 3 | Month 6 | Change | Mean difference  (95% CI) | 2p |
| --- | --- | --- | --- | --- | --- | --- | --- | --- | --- |
| SBP | I |  |  |  |  |  |  |  |  |
|  | G |  |  |  |  |  |  |  |  |
| DBP | I |  |  |  |  |  |  |  |  |
|  | G |  |  |  |  |  |  |  |  |
| HR | I |  |  |  |  |  |  |  |  |
|  | G |  |  |  |  |  |  |  |  |
| TC | I |  |  |  |  |  |  |  |  |
|  | G |  |  |  |  |  |  |  |  |
| TG | I |  |  |  |  |  |  |  |  |
|  | G |  |  |  |  |  |  |  |  |
| LDL-c | I |  |  |  |  |  |  |  |  |
|  | G |  |  |  |  |  |  |  |  |
| HDL-c | I |  |  |  |  |  |  |  |  |
|  | G |  |  |  |  |  |  |  |  |
| Non-HDL | I |  |  |  |  |  |  |  |  |
|  | G |  |  |  |  |  |  |  |  |

DBP: diastolic blood pressure; G: guideline; Grp: treatment group; HDL: high density; HR: heart rate; I: intensive; LDL: low density; SBP: systolic blood pressure; TC: total cholesterol; TG: triglycerides

**Table 4**. 24 hours ambulatory systolic and diastolic blood pressure and heart rate at baseline and on treatment (usually at 6 months), by treatment group: intensive (n=6) vs guideline (n=12). Data are mean (standard deviation); comparison by ANCOVA with mean difference on treatment adjusted for baseline.

| Month |  | Baseline | On-treatment | Mean difference | 2p |
| --- | --- | --- | --- | --- | --- |
| Systolic BP (mmHg) | Intensive |  |  |  |  |
|  | Guideline |  |  |  |  |
| Diastolic BP (mmHg) | Intensive |  |  |  |  |
|  | Guideline |  |  |  |  |
| Heart rate (bpm) | Intensive |  |  |  |  |
|  | Guideline |  |  |  |  |

**Table 5**. Addenbrooke’s Cognitive Examination-Revised, by treatment group: intensive versus guideline blood pressure lowering, and intensive versus guideline lipid lowering. Data are mean (standard deviation). Comparisons by t-test with Bonferroni adjustment for multiple comparisons.

| Group |  | Month 0 | Month 6 | Month 18 | Month 30 |
| --- | --- | --- | --- | --- | --- |
| BP | Patients |  |  |  |  |
|  | Intensive |  |  |  |  |
|  | Guideline |  |  |  |  |
|  | t-test |  |  |  |  |
| Lipids | Patients |  |  |  |  |
|  | Intensive |  |  |  |  |
|  | Guideline |  |  |  |  |
|  | t-test | - |  |  |  |

**Table 6**. Primary and secondary cognition and other functional measures by treatment group: intensive vs guideline blood pressure lowering. Comparison by multiple regression of on-treatment score with adjustment for baseline value and age, systolic blood pressure, total cholesterol, time since stroke, treatment assignment (intensive vs lipid guideline lowering vs none).

|  | Baseline | Intensive | Guideline | Difference | 2p |
| --- | --- | --- | --- | --- | --- |
| Follow-up (months) |  |  |  |  |  |
| Clinic |  |  |  |  |  |
| ACE-R |  |  |  |  |  |
| MMSE |  |  |  |  |  |
| MoCA |  |  |  |  |  |
| TICS |  |  |  |  |  |
| Trail |  |  |  |  |  |
| A time |  |  |  |  |  |
| A mistakes |  |  |  |  |  |
| B time |  |  |  |  |  |
| B mistakes |  |  |  |  |  |
| Stroop |  |  |  |  |  |
| 1 accuracy |  |  |  |  |  |
| 1 time |  |  |  |  |  |
| 2 accuracy |  |  |  |  |  |
| 2 time |  |  |  |  |  |
| 3 accuracy |  |  |  |  |  |
| 3 time |  |  |  |  |  |
| Interference accuracy |  |  |  |  |  |
| Interference time |  |  |  |  |  |
| Informant (IQ-code) |  |  |  |  |  |
| Knafelc ^17^ |  |  |  |  |  |
| Animal naming |  |  |  |  |  |
| Telephone |  |  |  |  |  |
| MMSE |  |  |  |  |  |
| TICS |  |  |  |  |  |
| Dementia |  |  |  |  |  |
| All (%) |  |  |  |  |  |
| Vascular |  |  |  |  |  |
| Mixed |  |  |  |  |  |
| Vascular |  |  |  |  |  |
| Stroke recurrence |  |  |  |  |  |
| Ischaemic |  |  |  |  |  |
| Haemorrhagic |  |  |  |  |  |
| Fatal |  |  |  |  |  |
| Non-fatal |  |  |  |  |  |
| MI |  |  |  |  |  |
| Vascular composite |  |  |  |  |  |
| Function |  |  |  |  |  |
| mRS |  |  |  |  |  |
| Barthel Index |  |  |  |  |  |
| HUS (EQ-5D) |  |  |  |  |  |
| EQ-VAS |  |  |  |  |  |
| ZDS |  |  |  |  |  |

ACE-R: Addenbrooke’s Cognitive Examination-R; EQ-VAS: EuroQoL-Visual Analogue Scale; HUS: Health Utility Status (from EuroQoL 5-dimensions); MMSE: Mini-Mental State Examination; MoCA: Montreal Cognitive Assessment; mRS: modified Rankin Scale; TICS: Telephone Interview Cognition Scale; t-MMSE: telephone-Mini-Mental State Examination; ZDS: Zung Depression Scale.

Patients who died were assigned the following scores: BI -5, ACE-R -1, EQ-VAS -1, MMSE -1, MoCA -1, Stroop accuracy -1, Stroop time, TICS -1, Trail making A & B -1, verbal fluency -1, HUS (from EQ-5D) 0, mRS 6, ZDS 102.5

**Table 7**. Primary and secondary cognition and other functional measures by treatment group: intensive vs guideline lipid lowering. Comparison by multiple regression of on-treatment score with adjustment for baseline value, age, systolic blood pressure, total cholesterol, time since stroke and treatment assignment (intensive vs guideline BP lowering).

As for table 6.

**Table 8**. Number of patients with one or more serious adverse events during treatment by organ class (plus selected events within organ classes) by treatment group: intensive vs guideline blood pressure lowering, and intensive vs guideline lipid lowering. Data are number (%). Comparison by Chi-square test

|  | BP |  | Lipid |  |
| --- | --- | --- | --- | --- |
| Cause | Intensive | Guideline | Intensive | Guideline |
| Cardiovascular |  |  |  |  |
| … |  |  |  |  |
| Total |  |  |  |  |

† p<0.05

**Table 9**. Fatal events during treatment, by treatment group. Data are number.

| Cause of death | BP |  | Lipid |  |
| --- | --- | --- | --- | --- |
|  | Intensive | Guideline | Intensive | Guideline |
| Dementia |  |  |  |  |
| Stroke |  |  |  |  |
| … |  |  |  |  |
| Total |  |  |  |  |

**Table 10**. Dementia and vascular events during treatment, by treatment group. Data are number of events.

| Event |  |  |  |  | BP |  | Lipid |  |
| --- | --- | --- | --- | --- | --- | --- | --- | --- |
|  |  |  |  |  | Intensive | Guideline | Intensive | Guideline |
| Dementia |  |  |  |  |  |  |  |  |
| All (%) |  |  |  |  |  |  |  |  |
| Vascular |  |  |  |  |  |  |  |  |
| Mixed |  |  |  |  |  |  |  |  |
| Vascular |  |  |  |  |  |  |  |  |
| Stroke recurrence |  |  |  |  |  |  |  |  |
| Ischaemic |  |  |  |  |  |  |  |  |
| Haemorrhagic |  |  |  |  |  |  |  |  |
| Fatal |  |  |  |  |  |  |  |  |
| Non-fatal |  |  |  |  |  |  |  |  |
| MI |  |  |  |  |  |  |  |  |
| Vascular composite |  |  |  |  |  |  |  |  |

**Table 11**. Protocol violations, by treatment group. Data are number (%).

|  | All | BP | lowering | Lipid | lowering |
| --- | --- | --- | --- | --- | --- |
|  |  | Intensive | Guideline | Intensive | Guideline |
| Inclusion criteria |  |  |  |  |  |
| … |  |  |  |  |  |
| On treatment |  |  |  |  |  |
| … |  |  |  |  |  |
| Treatment |  |  |  |  |  |
| … |  |  |  |  |  |
| Total number of patients |  |  |  |  |  |

**Figure 1**. CONSORT flow diagram of patient randomisation, outcome, and withdrawals. Screening for eligibility was not collected routinely. Data are number/Number (%).

| Randomised | BP |  |  | Lipid |  |  |
| --- | --- | --- | --- | --- | --- | --- |
|  | 🡿 |  | 🡾 | 🡿 |  | 🡾 |
| Treatment allocation | Intensive |  | Guideline | Intensive |  | Guideline |
| Number allocated (safety population) |  |  |  |  |  |  |
| Baseline data completed |  |  |  |  |  |  |
| Month 1 intensive follow-up |  |  |  |  |  |  |
| Died, withdrawn, not done |  |  |  |  |  |  |
| Month 2 intensive follow-up |  |  |  |  |  |  |
| Died, withdrawn, not done |  |  |  |  |  |  |
| Month 3 intensive follow-up |  |  |  |  |  |  |
| Died, withdrawn, not done |  |  |  |  |  |  |
| Month 6 clinic follow-up |  |  |  |  |  |  |
| Died, withdrawn, not done |  |  |  |  |  |  |
| Month 12 clinic follow-up |  |  |  |  |  |  |
| Died, withdrawn, not done |  |  |  |  |  |  |
| Month 12 telephone follow-up |  |  |  |  |  |  |
| Died, withdrawn, not done |  |  |  |  |  |  |
| Month 18 clinic follow-up |  |  |  |  |  |  |
| Died, withdrawn, not done |  |  |  |  |  |  |
| Month 24 clinic follow-up |  |  |  |  |  |  |
| Died, withdrawn, not done |  |  |  |  |  |  |
| Month 24 telephone follow-up |  |  |  |  |  |  |
| Died, withdrawn, not done |  |  |  |  |  |  |
| … |  |  |  |  |  |  |
| Final telephone follow-up |  |  |  |  |  |  |
| Died, withdrawn, not done |  |  |  |  |  |  |
|  |  |  |  |  |  |  |

**Figure 2**. Systolic and diastolic blood pressure throughout the trial, by intensive vs guideline BP lowering. Mean and standard error of mean.

**Figure 3**. Total cholesterol and LDL-cholesterol throughout the trial, by intensive vs guideline lipid lowering. Mean and standard error of mean.

**Figure 4**. Addenbrooke’s Cognitive Examination-Revised score during follow-up, by treatment group: (a) intensive vs guideline blood pressure lowering; and (b) intensive vs guideline lipid lowering. Mean and standard error of mean.
